# Exploring Rhenium Arene Piano-Stool Chemistry with [Re(η^6^-C_6_H_6_)(NCCH_3_)_3_]^+^: A Powerful Semi-Solvated Precursor

**DOI:** 10.1021/acs.inorgchem.2c04346

**Published:** 2023-02-28

**Authors:** Robin Bolliger, Lukas Siebenmann, Emily Wolf, Megan Ross, Giuseppe Meola, Olivier Blacque, Henrik Braband, Roger Alberto

**Affiliations:** Department of Chemistry, University of Zurich, Winterthurerstrasse 190, 8057 Zürich, Switzerland

## Abstract

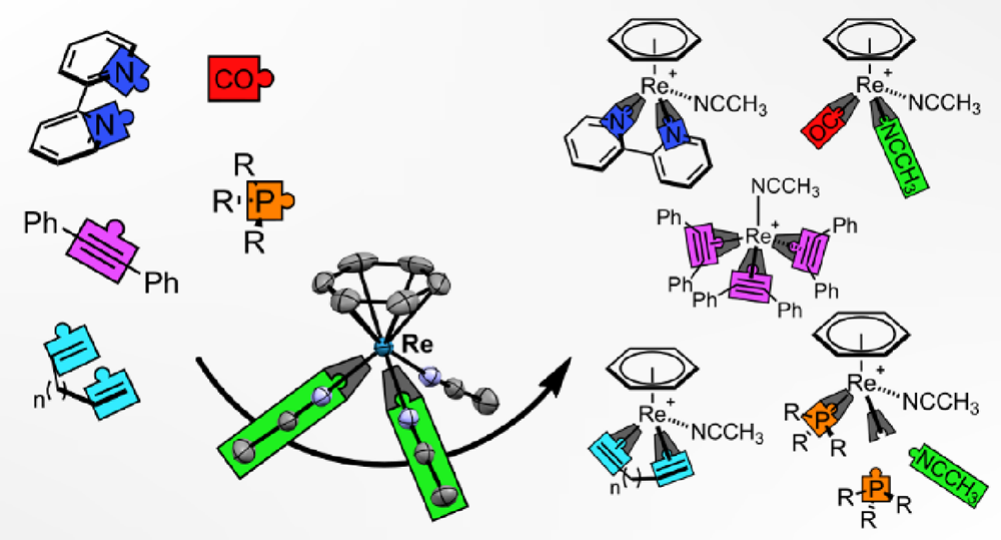

Thermal treatment of the Re^III^ hydride complex [ReH(η^5^-C_6_H_7_)(η^6^-C_6_H_6_)]^+^ in CH_3_CN results in the formation of [Re(η^6^-C_6_H_6_)(NCCH_3_)_3_]^+^. This semi-solvated complex is remarkably stable under an ambient atmosphere and exhibits a fast CH_3_CN self-exchange, which facilitates substitution reactions. The CH_3_CN ligands are replaced by σ-donating phosphines such as trimethyl phosphine (PMe_3_), triphenyl phosphine (PPh_3_), or the bidentate 1,2-bis(diphenylphosphino)ethane (dppe) to afford [Re(η^6^-C_6_H_6_)(NCCH_3_)_3–*x*_(PR_3_)_*x*_]^+^ (if R = Me, then *x* = 2; if R = Ph, then *x* = 1 or 2) or [Re(η^6^-C_6_H_6_)(dppe)(NCCH_3_)]^+^, respectively. [Re(η^6^-C_6_H_6_)(NCCH_3_)_3_]^+^ also reacts with π-acceptors such as 2,2′-bipyridine (bipy), 1,10-phenanthroline (phen), or CO (1 atm) to give [Re(η^6^-C_6_H_6_)(L)(NCCH_3_)]^+^ (L = bipy or phen) and [Re(η^6^-C_6_H_6_)(CO)(NCCH_3_)_2_]^+^, respectively. The latter does not show any signs of decomposition after being exposed to an ambient atmosphere for multiple days. Additionally, [Re(η^6^-C_6_H_6_)(NCCH_3_)_3_]^+^ reacts with π-donors such as the dienes 2,3-dimethyl-1,3-butadiene (DMBD), norbornadiene (NBD), or 1,5-cyclooctadiene (COD) to give [Re(η^6^-C_6_H_6_)(η^4^-diene)(NCCH_3_)]^+^ (diene = DMBD, NBD, and COD). All three complexes are extremely stable and do not decompose during purification by preparative high-performance liquid chromatography (aqueous acidic gradient). In the presence of 18-crown-6, [Re(η^6^-C_6_H_6_)(NCCH_3_)_3_]^+^ reacts with lithium cyclopentadienyl to give the sandwich complex [Re(η^5^-C_5_H_5_)(η^6^-C_6_H_6_)]. Loss of the coordinated benzene was observed when treating [Re(η^6^-C_6_H_6_)(NCCH_3_)_3_]^+^ with diphenylacetylene (PhC≡CPh), yielding the tetra-coordinated [Re(NCCH_3_)(η^2^-PhC≡CPh)_3_]^+^.

## Abstract 2

[Re(η^6^-C_6_H_6_)(NCCH_3_)_3_]^+^ is a convenient precursor for Re^I^ arene piano-stool chemistry. The fast acetonitrile exchange facilitates substitution reactions with various ligands such as σ-donating phosphines, π-donating dienes, or π-acceptors such as CO or polypyridines. Loss of the benzene ligand is observed for substitution reactions with diphenylacetylene (PhC≡CPh), yielding the tetra-coordinated [Re(NCCH_3_)(η^2^-PhC≡CPh)_3_]^+^.

## Introduction

Piano-stool complexes, also known as half-sandwich complexes, are one of the core classes of organometallic chemistry. One of the most representative examples is [Co(η^5^-C_5_H_5_)(CO)_2_] as applied in Vollhardt cyclization reactions of alkynes.^1^ Piano-stool complexes have at least generated interest in various other fields including bioorganometallic chemistry. Exemplarily are [Ru(η^6^-arene)L_3_]^2+^ or [Re(η^5^-C_5_R_5_)(CO)_3_] complexes incorporating biologically active lead structures in the cyclopentadienyl framework.^2−6^

From a synthetic chemist’s point of view, semi-solvated piano-stool complexes are attractive as powerful synthons for the syntheses of sandwich or half-sandwich complexes. For example, [Co(η^4^-C_4_Me_4_)(NCCH_3_)_3_]^+^ exchanges its acetonitrile ligands with a variety of ligands, including arenes and cyclopentadienyl or indenyl derivatives.^7,8^ Due to the easy replacement of the CH_3_CN ligands, [Ru(η^5^-C_5_H_5_)(NCCH_3_)_3_]^+^ as well as its pentamethylcyclopentadienyl derivative possess versatile coordination chemistries and have also been applied in catalytic processes, e.g., triene cyclization or hydrogenation of alkynes.^9−13^ Reports discussing the reactivity of their benzene analogue [Ru(η^6^-C_6_H_6_)(NCCH_3_)_3_]^2+^ are scarce. Reported substitution reactions are mainly limited to phosphines and polypyridines.^14,15^ Even rarer are reports of substitution reactions with the Os analogue [Os(η^6^-C_6_H_6_)(NCCH_3_)_3_]^2+^.^16,17^

There is only a limited number of known Re arene piano-stool precursors. One of the first was [ReI(η^6^-C_6_H_6_)(PMe_3_)_2_] obtained from the dimer [Re_2_(η^6^-C_6_H_6_)_2_(PMe_3_)_4_], which is produced by metal vapor synthesis.^18^ Some substitution and addition reactions have been reported for [Re(η^6^-C_6_H_6_)(CO)_3_]^+^, the benzene ligand of which is easily displaced by CH_3_CN to yield *fac*-[Re(CO)_3_(NCCH_3_)_3_]^+^.^19−21^ More recently, we showed that the naphthalene ligand in [Re(η^6^-C_6_H_6_)(η^6^-C_10_H_8_)]^+^ is replaced to yield piano-stool complexes [Re(η^6^-C_6_H_6_)R_3_]^+^ (R = isonitriles or phosphites). [Re(η^6^-C_6_H_6_)(η^6^-C_10_H_8_)]^+^ is formed by a modified Fischer–Hafner synthesis, starting from ReO_4_^–^, elemental Zn, and AlCl_3_ in a benzene/naphthalene solvent mixture. Hence, formation of the mixed arene product is paralleled by the formation of the homoleptic complexes [Re(η^6^-C_6_H_6_)_2_]^+^ and [Re(η^6^-C_10_H_8_)_2_]^+^. Chromatographic separation of the products by preparative high-performance liquid chromatography (HPLC) is required, which is not always available and hence limits the availability of this precursor.^22^

Only recently, we reported the synthesis of the semi-solvated [Re(η^6^-C_6_H_6_)(NCCH_3_)_3_]^+^ ([**2**]^+^) by the reaction of the Re^III^ hydride [ReH(η^5^-C_6_H_7_)(η^6^-C_6_H_6_)]^+^ ([**1**]^+^) with CH_3_CN via the cyclohexadiene complex [Re(η^6^-C_6_H_6_)(η^4^-C_6_H_8_)(NCCH_3_)]^+^. The cyclohexadiene ligand exchanges easily in the presence of excess CH_3_CN, yielding [**2**]^+^.^23^ Complex [**2**]^+^ is a synthon for the homoleptic, fully solvated Re^II^ complex [Re(NCCH_3_)_6_]^2+^. Its potential as a starting material for further Re half-sandwich complexes has though not yet been evaluated.^24^ We were thus motivated to explore the reactivities of the easily accessible [**2**]^+^ with a broad spectrum of ligands, ranging from coordination compounds to classical organometallic ligands, to better understand the electronic properties of this Re^I^ center.

## Results and Discussion

**Scheme 1:**
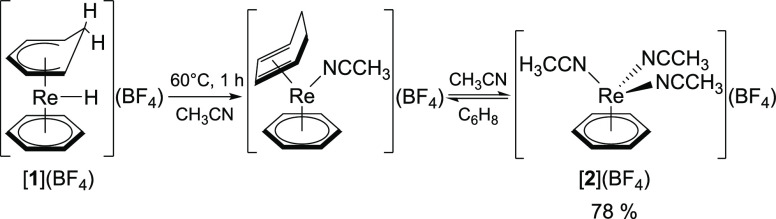
Thermal Treatment of the Re^III^ Hydride [**1**](BF_4_) in CH_3_CN Forms the Semi-Solvated Piano-Stool Complex [**2**](BF_4_) via a Cyclohexadiene Intermediate

Heating the Re^III^ hydride [**1**](BF_4_) in CH_3_CN at 60 °C for 1 h results in the formation of the piano-stool complex [**2**](BF_4_) via the key-intermediate [Re(η^6^-C_6_H_6_)(η^4^-C_6_H_8_)(NCCH_3_)]^+^. For later substitution reactions, [**2**]^+^ is formed *in situ* only. Nonetheless, isolation of the semi-solvated complex in 78% yield was achieved by addition of diethyl ether (Et_2_O) to a concentrated CH_3_CN solution of [**2**](BF_4_) (Scheme 1). Spectroscopic characterization of [**2**](BF_4_) as well as all described complexes can be found in Figures S1–S37. The CH_3_CN exchange in [**2**]^+^ is fast, and its exchange rate is not assessable by ^1^H NMR. The CH_3_CN/CD_3_CN exchange is already complete within the time frame required for sample preparation and measurement. The exchange is therefore much faster than for the analogous and isoelectronic [Ru(η^6^-C_6_H_6_)(NCCH_3_)_3_]^2+^ (*k* ≈ 4.1 × 10^–5^ s^–1^).^25^ Dissolved in CH_3_CN, [**2**](BF_4_) is remarkably stable and only little decomposition (<15 mol %) is observed after 15 h under an ambient atmosphere. This unexpected stability is portrayed well by electrochemical data. The cyclic voltammogram of [**2**](BF_4_) shows an irreversible oxidation at +0.18 V vs [Fe(η^5^-C_5_H_5_)_2_]^+^/[Fe(η^5^-C_5_H_5_)_2_] (Fc^+^/Fc) in CH_3_CN (0.1 M (NBu_4_)(PF_6_); Figure S38).

**Figure 1:**
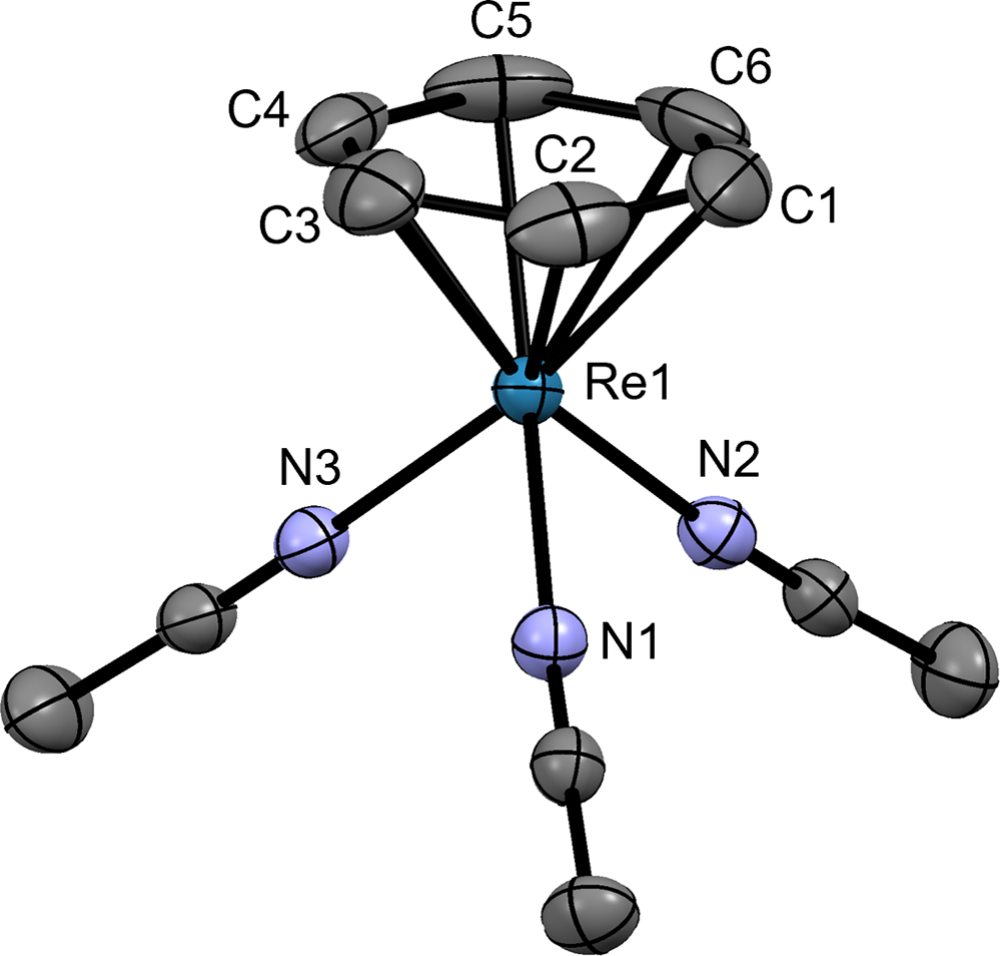
Ellipsoid displacement representation of the cation in the structure of [**2**](BF_4_)·0.5 CH_3_CN (ellipsoids drawn at 50% probability). Hydrogen atoms, the tetrafluoroborate anion, and a solvent molecule (0.5 CH_3_CN) are omitted for clarity. Selected bond lengths [Å]: Re1–C1 2.195(3), Re1–C2 2.165(3), Re1–C3 2.204(3), Re1–C4 2.178(3), Re1–C5 2.203(3), Re1–C6 2.162(3), Re1–N1 2.085(2), Re1–N2 2.087(2), Re1–N3 2.090(2).

[**2**](BF_4_)·0.5 CH_3_CN crystallized after slow evaporation of a CH_3_CN/toluene solution (Figure 1). The Re–N bond lengths range from 2.085(2) to 2.090(2) Å and are therefore significantly longer than in [Re(NCCH_3_)_6_](OTf)_2_ (2.041(4)–2.059(3) Å).^26^ The Re–C bond lengths in [**2**]^+^ (2.165(3)–2.204(3) Å) are shorter than in [Re(η^6^-C_6_H_6_)_2_](PF_6_) (2.194(9)–2.301(9) Å).^27^ Overall, the structural features resemble the ones found in [Ru(η^6^-C_6_H_6_)(NCCH_3_)_3_](PF_6_)_2_.^25^

**Scheme 2:**
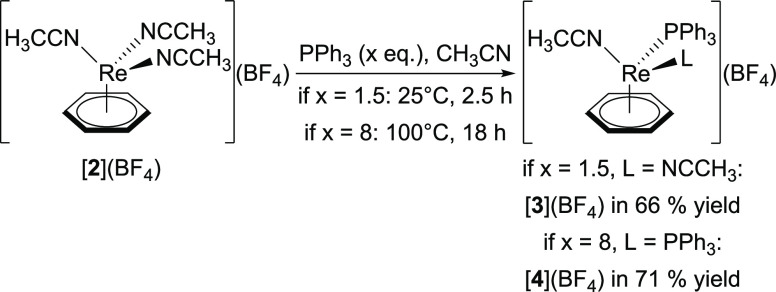
Depending on the Reaction Conditions, Treatment of [**2**](BF_4_) with PPh_3_ Results in Mono- or Di-Substitution Forming [**3**](BF_4_) or [**4**](BF_4_), Respectively

[**2**](BF_4_) reacts readily with phosphines. The reaction of triphenyl phosphine (PPh_3_, 1.5 equiv) with *in situ* formed [**2**](BF_4_) in CH_3_CN at 25 °C gave the mono-substituted product [Re(η^6^-C_6_H_6_)(NCCH_3_)_2_(PPh_3_)](BF_4_) ([**3**](BF_4_)) in 66% yield. Di-substitution required harsher conditions (100 °C, 18 h, 8 equiv of PPh_3_) but yielded [Re(η^6^-C_6_H_6_)(NCCH_3_)(PPh_3_)_2_](BF_4_) ([**4**](BF_4_)) in 71% (Scheme 2). This second substitution may be slow due to steric constraints (vide infra). However, the self-exchange rate of CH_3_CN ligands in mono-substituted complexes is orders of magnitudes slower than in [**2**]^+^ (vide infra). Thus, kinetics in a dissociatively driven reaction are likely to be co-responsible for this slow reaction. The ^1^H NMR signal of the coordinated CH_3_CN ligands in [**3**]^+^ is found as a doublet at δ = 2.19 ppm (*J* = 1.9 Hz; Figure S3) due to scalar coupling with the ^31^P nucleus in the PPh_3_ ligand. Accordingly, the analogous signal for the di-substituted product [**4**]^+^ is a triplet (δ = 2.43 ppm, *J* = 1.9 Hz; Figure S6).

**Figure 2:**
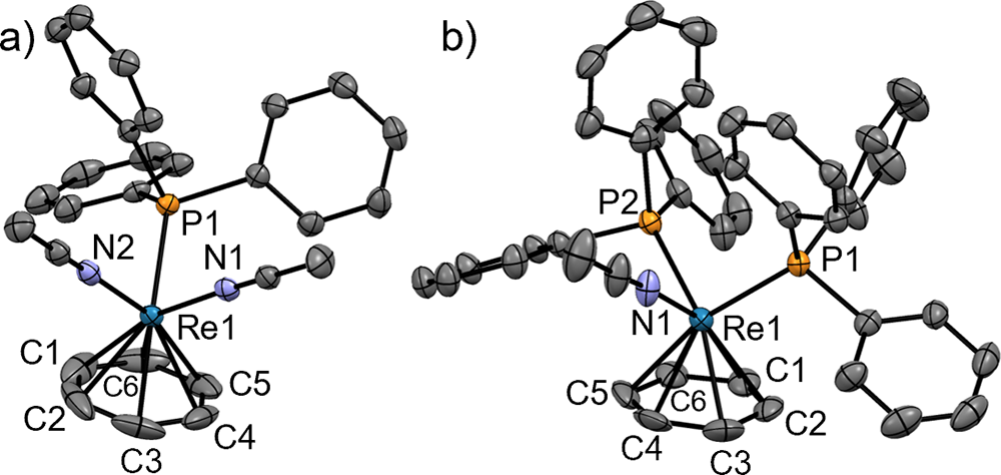
Ellipsoid displacement representation of the cations in the structures of (a) [**3**](BF_4_) and (b) [**4**](BF_4_) (ellipsoids drawn at 50% probability). In panel (a), only half of the asymmetric unit is depicted. All structural features of both species in the asymmetric unit are similar. Hydrogen atoms and tetrafluoroborate anions are omitted for clarity. Selected bond lengths [Å]: (a) Re1–C1 2.236(3), Re1–C2 2.207(3), Re1–C3 2.234(3), Re1–C4 2.207(3), Re1–C5 2.226(3), Re1–C6 2.188(3), Re1–N1 2.075(2), Re1–N2 2.071(2), Re1–P1 2.3940(6); (b) Re1–C1 2.263(4), Re1–C2 2.233(4), Re1–C3 2.271(5), Re1–C4 2.216(4), Re1–C5 2.241(4), Re1–C6 2.232(4), Re1–N1 2.059(4), Re1–P1 2.4166(9), Re1–P2 2.4215(10).

The Re–P bonds in the di-substituted [**4**](BF_4_) (2.4166(9) and 2.4215(10) Å) are slightly elongated as compared to the ones in the mono-substituted [**3**](BF_4_) (2.3940(6) and 2.3843(6) Å, two cations are in the asymmetric unit; Figure 2). The Re–N bond length in [**4**](BF_4_) (2.059(4) Å) is slightly shorter than the ones in [**3**](BF_4_) (2.068(2)–2.076(2) Å). The average Re–C bond length in [**3**](BF_4_) (2.212(3) Å) is shorter than in the di-substituted [**4**](BF_4_) (2.243(4) Å). The structural features of [**3**](BF_4_) are similar to those reported for [Ru(η^6^-C_6_H_6_)(NCCH_3_)_2_(PPh_3_)](BF_4_)_2_.^15^ To the best of our knowledge, a structural analogue of [**4**](BF_4_) does not exist. However, its structure is similar to the cyclopentadienyl complex [Os(η^5^-C_5_H_5_)(NCCH_3_)(PPh_3_)_2_](BF_4_).^28^

**Scheme 3:**
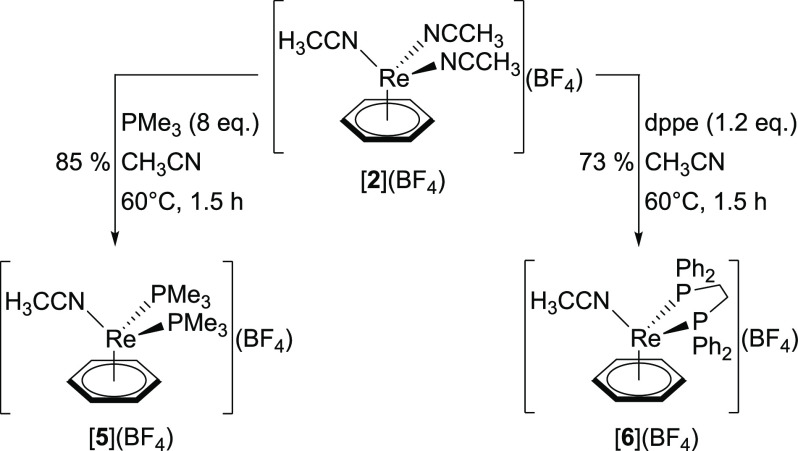
The Reaction of [**2**](BF_4_) with PMe_3_ Gives the Di-Substituted Product [**5**](BF_4_). Treatment of [**2**](BF_4_) with the Bidentate dppe Yields [**6**](BF_4_)

Di-substitution is facilitated with phosphines of smaller cone angles, e.g., PMe_3_.^29^ Heating [**2**](BF_4_) with PMe_3_ (8 equiv) in CH_3_CN at 60 °C for 1.5 h results in the exclusive formation of the di-substituted species [Re(η^6^-C_6_H_6_)(NCCH_3_)(PMe_3_)_2_](BF_4_) ([**5**](BF_4_)) in 85% yield (Scheme 3). Werner and Werner reported the reaction of [Ru(η^6^-C_6_H_6_)(NCCH_3_)_3_]^+^ with PMe_3_ forming the cyclohexadienyl species [Ru(η^5^-C_6_H_6_PMe_3_)(NCCH_3_)(PMe_3_)_2_]^2+^.^30^ An analogous Re species was not detected. Potentially, the reduced charge leads to less polarization of the benzene ligand, which prevents nucleophilic addition of PMe_3_.

**Figure 3:**
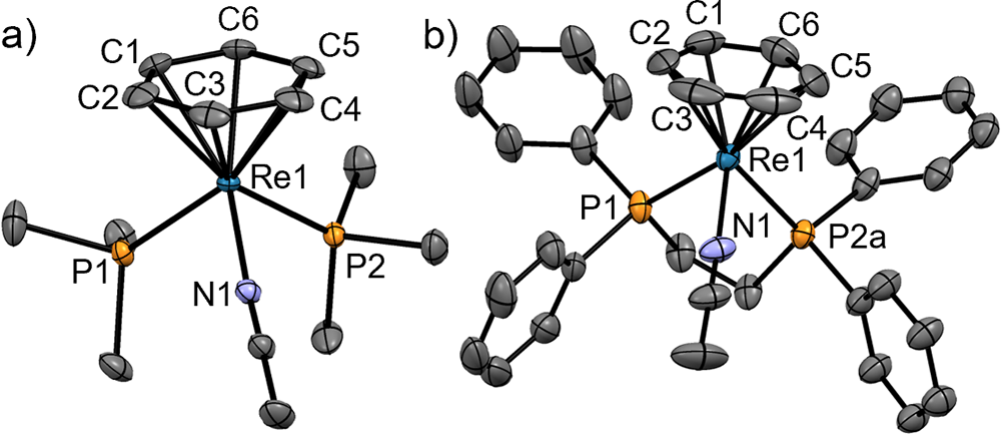
Ellipsoid displacement representation of the cations in the structures of (a) [**5**](BF_4_) and (b) [**6**](BF_4_) (ellipsoids drawn at 50% probability). In panel (a), only half of the asymmetric unit is depicted. Hydrogen atoms, tetrafluoroborate anions, and in panel (b), a partial disorder on the dppe ligand are omitted for clarity. Selected bond lengths [Å]: (a) Re1–C1 2.206(3), Re1–C2 2.268(3), Re1–C3 2.228(3), Re1–C4 2.251(3), Re1–C5 2.231(3), Re1–C6 2.227(3), Re1–N1 2.072(2), Re1–P1 2.3683(9), Re1–P2 2.3686(9); (b) Re1–C1 2.219(3), Re1–C2 2.236(3), Re1–C3 2.224(3), Re1–C4 2.225(3), Re1–C5 2.235(3), Re1–C6 2.217(3), Re1–N1 2.064(2), Re1–P1 2.3523(6), Re1–P2a 2.359(3).

[**5**](BF_4_) crystallized in the orthorhombic space group *Fdd*2. The Re–P bonds are shorter (2.365(1)–2.3686(9) Å, two cations in the asymmetric unit; Figure 3a) than those of its PPh_3_ analogue [**4**](BF_4_) (2.4166(9) and 2.4215(10) Å), while the Re–N bond lengths are slightly longer (2.065(3) and 2.072(2) Å in [**5**](BF_4_) vs 2.059(4) Å in [**4**](BF_4_)). The Re–C bonds are in a similar range (2.206(3)–2.268(3) Å) as in [**3**](BF_4_) or [**4**](BF_4_), respectively.

Complex [**2**]^+^ also reacts with bidentate phosphines such as 1,2-bis(diphenylphosphino)ethane (dppe). Treating [**2**](BF_4_) with dppe (1.2 equiv) in CH_3_CN at 60 °C for 1.5 h affords [Re(η^6^-C_6_H_6_)(dppe)(NCCH_3_)](BF_4_) ([**6**](BF_4_)) in 73% yield (Scheme 3). Complex [**6**](BF_4_) crystallized in the monoclinic space group *P*2_1_/*n* (Figure 3b). In the structure, the Re–C bond lengths range from 2.218(3) to 2.236(4) Å and the Re–N bond length is 2.064(2) Å. These values are comparable to the ones in the previously described complexes. The Re–P bonds (2.3523(7)–2.361(3) Å) are shorter than in the di-substituted complex [**4**](BF_4_) (2.4166(9) and 2.4215(10) Å).

**Scheme 4:**
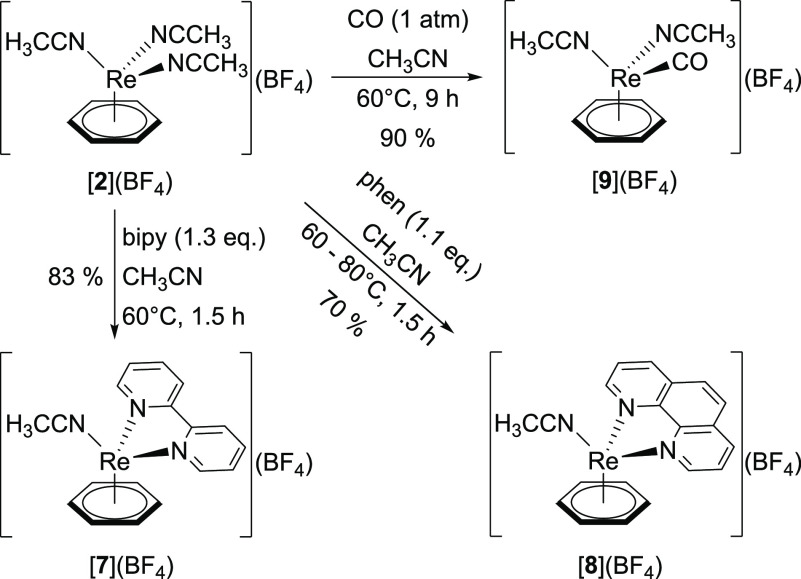
[**2**](BF_4_) Reacts with π-Acceptors Such as the Polypyridines bipy and phen or CO to Give the Complexes [**7**](BF_4_), [**8**](BF_4_), and [**9**](BF_4_), Respectively

Complex [**2**]^+^ reacts also with π-acceptors such as 2,2′-bipyridine (bipy) or 1,10-phenanthroline (phen). The reactions of [**2**](BF_4_) with bipy (1.1 equiv) or phen (1.1 equiv) in CH_3_CN gave [Re(η^6^-C_6_H_6_)(bipy)(NCCH_3_)](BF_4_) ([**7**](BF_4_)) in 83% yield and [Re(η^6^-C_6_H_6_)(phen)(NCCH_3_)](BF_4_) ([**8**](BF_4_)) in 70% yield (Scheme 4). Both complexes have a deep purple color, reflected by UV/vis absorptions at λ = 569 nm (for [**7**]^+^) and λ = 557 nm (for [**8**]^+^) (Figures S40 and S41, respectively).

In CD_3_CN, both complexes slowly decompose under an ambient atmosphere, indicated by the appearance of uncoordinated benzene according to ^1^H NMR studies. Neither uncoordinated bipy (or phen) nor new ^1^H NMR signals are observed, indicating the formation of a paramagnetic Re complex with bipy (or phen) still coordinated. Ultimately, the identities of the decomposition products were not assessed.

**Figure 4:**
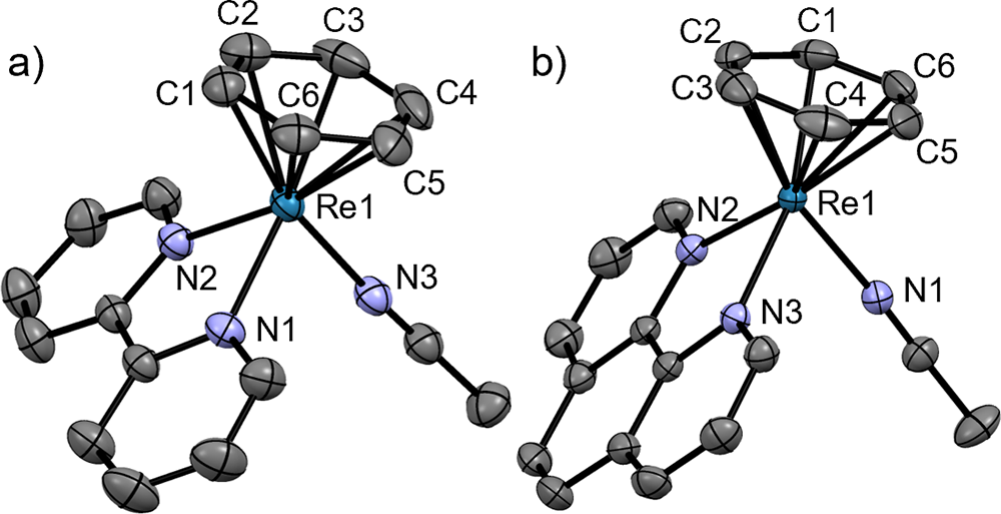
Ellipsoid displacement representation of the cations in the structures of (a) [**7**](BF_4_)·0.5 CH_3_CN and (b) [**8**](BF_4_)·CH_2_Cl_2_ (ellipsoids drawn at 50% probability). In panel (a), only half of the asymmetric unit is depicted. Hydrogen atoms, tetrafluoroborate anions, and solvent molecules are omitted for clarity. Selected bond lengths [Å]: (a) Re1–C1 2.205(3), Re1–C2 2.163(3), Re1–C3 2.211(3), Re1–C4 2.200(4), Re1–C5 2.228(3), Re1–C6 2.192(3), Re1–N1 2.103(2), Re1–N2 2.100(2), Re1–N3 2.081(2); (b) Re1–C1 2.203(2), Re1–C2 2.177(2), Re1–C3 2.200(2), Re1–C4 2.174(2), Re1–C5 2.202(2), Re1–C6 2.195(2), Re1–N1 2.0753(17), Re1–N2 2.1149(16), Re1–N3 2.1229(16).

The structures of [**7**](BF_4_)·0.5 CH_3_CN and [**8**](BF_4_)·CH_2_Cl_2_ are similar (Figure 4). For both complexes, the Re–NCCH_3_ bond lengths (2.081(2) Å for [**7**]^+^ and 2.0753(17) Å for [**8**]^+^) are shorter than the Re–N bond lengths to the N-heterocycles (2.100(2)–2.117(2) Å for [**7**]^+^ and 2.1149(16) and 2.1229(16) Å for [**8**]^+^). The Re–C bond lengths are almost identical with 2.161(4)–2.228(3) Å for [**7**]^+^ and 2.174(2)–2.203(2) Å for [**8**]^+^. Compounds [**4**]^+^, [**7**]^+^, and [**8**]^+^ show similar structural features as found in analogous Ru^II^ complexes.^15^

[Re(η^6^-C_6_H_6_)(CO)(NCCH_3_)_2_](BF_4_) ([**9**](BF_4_)) forms in 90% yield upon heating [**2**](BF_4_) at 60 °C for 9 h in CH_3_CN under a CO atmosphere (1 atm; Scheme 4). Coordination of CO is manifested by a single, intense IR absorption at 1900 cm^–1^ as well as a ^13^C NMR signal at δ = 200.5 ppm (Figure S22). In contrast to [**7**]^+^ and [**8**]^+^, [**9**]^+^ is stable under an ambient atmosphere, even in solution. This is reflected by cyclic voltammetry that displays an irreversible oxidation at +0.63 V vs Fc^+^/Fc in CH_3_CN (Figure S39), anodically shifted by 0.45 V in comparison to [**2**]^+^. The ^1^H NMR signal corresponding to the coordinated CH_3_CN ligands in [**9**]^+^ (δ = 2.52 ppm) barely shows signs of nitrile exchange in CD_3_CN, while the corresponding signals in [**7**]^+^ (δ = 2.14 ppm) and [**8**]^+^ (δ = 2.01 ppm) disappear within minutes in CD_3_CN (Figures S17, S19, and S21).

**Figure 5:**
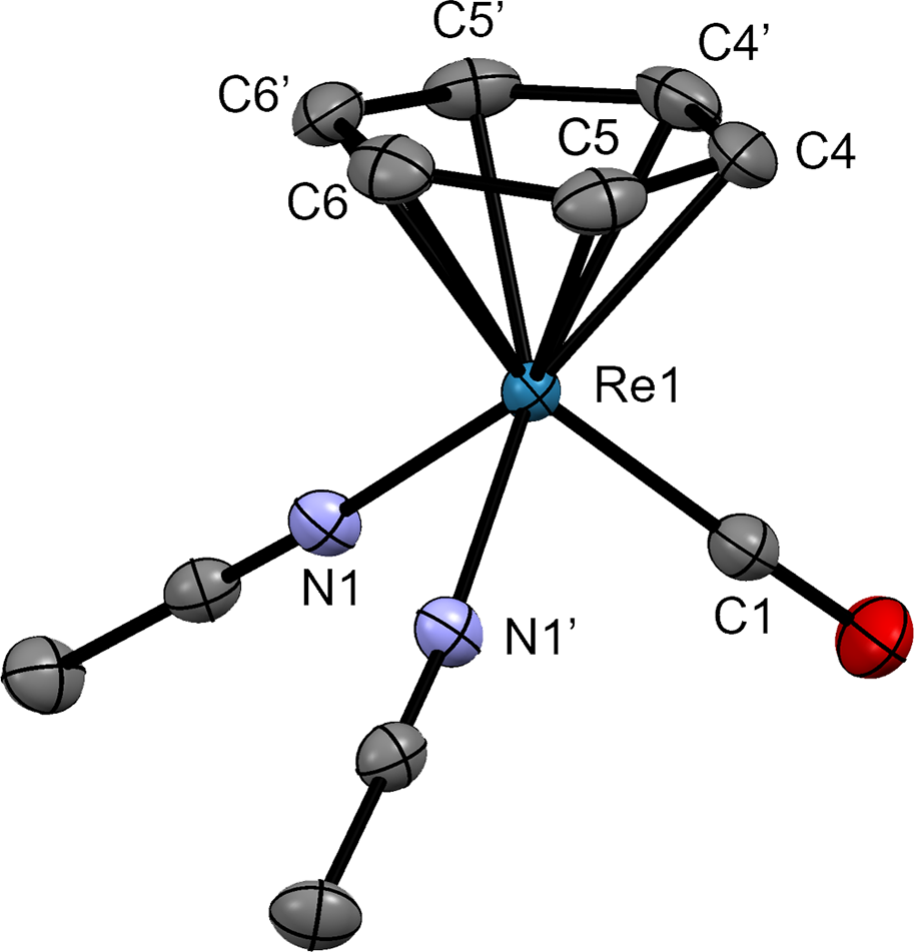
Ellipsoid displacement representation of the cation in the structures of [**9**](BF_4_) (ellipsoids drawn at 50% probability). Hydrogen atoms and tetrafluoroborate anion are omitted for clarity. Selected bond lengths [Å]: Re1–C1 1.913(4), Re1–C4 2.245(2), Re1–C5 2.210(3), Re1–C6 2.294(3), Re1–N1 2.082(2).

[**9**](BF_4_) crystallized in the monoclinic space group *P*2_1_/*m* (Figure 5). The Re–CO bond length is 1.913(4) Å and hence shorter than the Re–C bond lengths to the arene ligand (2.210(3)–2.294(3) Å). The Re–N bond length (2.082(2) Å) is similar as in the previously described nitrile complexes.

**Scheme 5:**
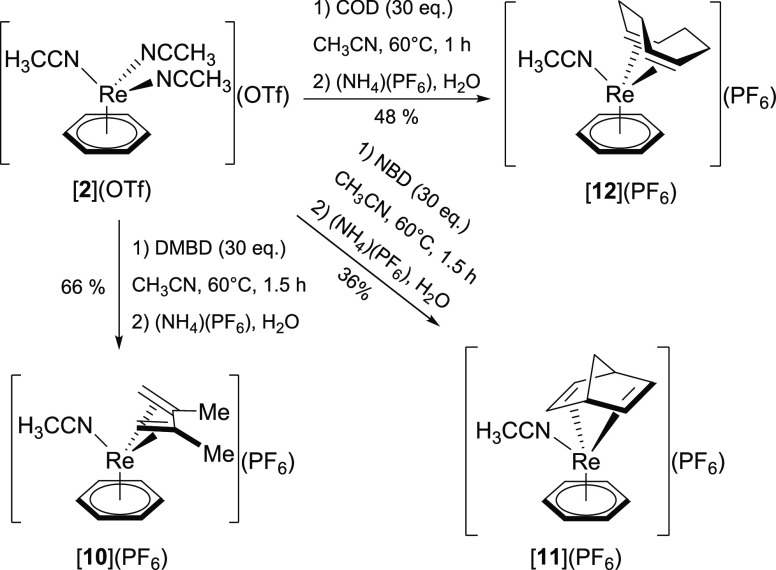
Complex [**2**](OTf) Reacts with Dienes Such as DMBD, NBD, and COD to Give the Complexes [**10**](PF_6_), [**11**](PF_6_), and [**12**](PF_6_), Respectively, after Subsequent Anion Exchange with (NH_4_)(PF_6_)

In a previous report, we showed that the equilibrium between [**2**]^+^ and its cyclohexadiene precursor [Re(η^6^-C_6_H_6_)(η^4^-C_6_H_8_)(NCCH_3_)]^+^ lies on the side of the of the cyclohexadiene complex (*K*_eq_ = 6.21 × 10^–3^ M^–1^). This unexpected stability of the {Re(η^4^-C_6_H_8_)}^+^ fragment was exploited for the isolation of [Re(η^6^-C_6_H_6_)(η^4^-C_6_H_8_)(NCCH_3_)]^+^. Addition of 30 equiv of 1,3-cyclohexadiene to [**2**]^+^ afforded the cyclohexadiene complex.^23^ The same strategy as with 1,3-cyclohexadiene was applied to dienes such as 2,3-dimethyl-1,3-butadiene (DMBD), norbornadiene (NBD), or 1,5-cyclooctadiene (COD), yielding products of the general formula [Re(η^6^-C_6_H_6_)(η^4^-diene)(NCCH_3_)](PF_6_) ([**10**](PF_6_), diene = DMBD; [**11**](PF_6_), diene = NBD; [**12**](PF_6_), diene = COD) in 36–66% yield (Scheme 5). All three complexes are sufficiently stable to be purified by preparative HPLC (aqueous acidic mobile phase). However, we note that prolonged storage under an ambient atmosphere leads to slow decomposition of the complexes.

The complexes were characterized by HR-ESI-MS and ^1^H and ^13^C NMR. Compared to the ^1^H and ^13^C NMR spectra of the uncoordinated dienes NBD and COD (Figures S36 and S37, respectively), all signals in [**11**]^+^ and [**12**]^+^, respectively, are split due to the reduced symmetry (Figures S25–S28). In the case of [**10**]^+^, the number of ^1^H and ^13^C NMR signals originating from the DMBD ligand is equal as found in the uncoordinated DMBD (Figures S23–S35). However, similar as in [**11**]^+^ and [**12**]^+^, coordination of the diene leads to a strong upfield shift of its NMR signals. For example, the ^13^C NMR signals of DMBD in acetone-*d*_6_ are at δ = 144.2 (C=CH_2_), 113.6 (C=CH_2_), and 20.8 (CH_3_) ppm. In [**10**]^+^, the corresponding signals are at δ = 102.8 (C=CH_2_), 46.5 (C=CH_2_), and 19.8 (CH_3_) ppm. Such upfield shifts of NMR signals were also observed in similar complexes such as [Co(η^4^-DMBD)(η^4^-COD)]^−^ and [Mo(η^4^-DMBD)(dppe)].^31,32^

**Figure 6:**
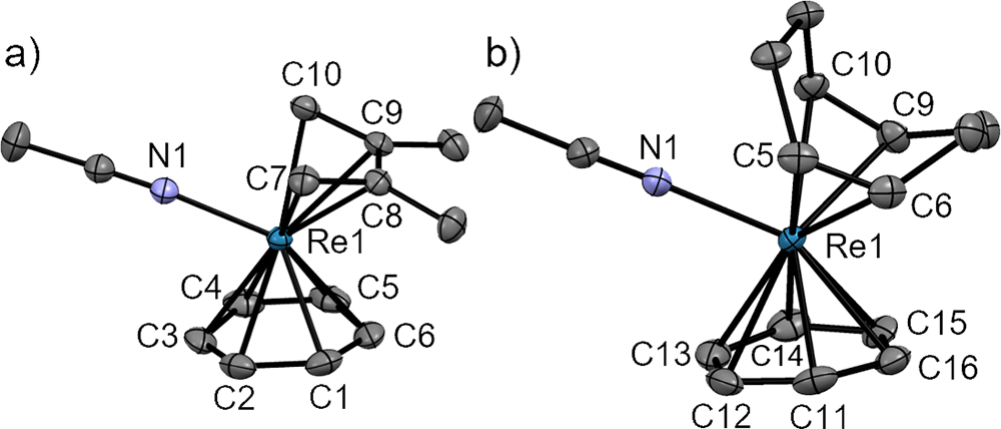
Ellipsoid displacement representation of the cations in the structures of (a) [**10**](PF_6_)·CH_3_CN and (b) [**12**](PF_6_) (ellipsoids drawn at 50% probability). Hydrogen atoms, hexafluorophosphate anions, and (a) solvent molecules (CH_3_CN) are omitted for clarity. Selected bond lengths [Å]: (a) Re1–C1 2.205(2), Re1–C2 2.231(2), Re1–C3 2.277(2), Re1–C4 2.235(2), Re1–C5 2.202(2), Re1–C6 2.263(2), Re1–C7 2.233(2), Re1–C8 2.249(2), Re1–C9 2.251(2), Re1–C10 2.234(2), Re1–N1 2.073(2); (b) Re1–C5 2.282(5), Re1–C6 2.289(3), Re1–C9 2.281(5), Re1–C10 2.286(5), Re1–C11 2.221(5), Re1–C12 2.278(5), Re1–C13 2.265(4), Re1–C14 2.238(5), Re1–C15 2.242(7), Re1–C16 2.252(5), Re1–N1 2.100(3).

There are only a few reports of Re complexes with dienes such as DMBD or COD, and to the best of our knowledge, no respective crystal structure data is available.^33−35^ Hence, [**10**](PF_6_)·CH_3_CN and [**12**](PF_6_) (anion exchange was performed after preparative HPLC for crystallization purposes) are the first examples of these types of complexes, characterized by single-crystal X-ray diffraction analysis (Figure 6). [**10**](PF_6_)·CH_3_CN crystallized in the monoclinic space group *P*2_1_/*n*, while [**12**](PF_6_) crystallized in the orthorhombic space group *Pna*2_1_. In [**10**]^+^, the distances of the Re atom to the terminal C atoms (Re1–C7: 2.233(2) Å and Re1–C10: 2.234(2) Å) are almost identical as the distances to the central C atoms of the DMBD ligand (Re1–C8: 2.249(2) Å and Re1–C9: 2.251(2) Å). In addition, the C–C bond lengths in the DMBD ligand are almost equal (C7–C8: 1.418(3) Å, C8–C9: 1.433(3) Å, and C9–C10 1.414(3)). The equalization of the C–C bond lengths in DMBD originates from π-back bonding. Similar values were found for the DMBD ligand in [Mo(η^4^-DMBD)_2_(dppe)](BPh_4_).^32^ The Re–C_benzene_ bond lengths in [**10**]^+^ range from 2.2046(19) to 2.2766(19) Å and are almost identical to the ones in [**12**]^+^ (2.221(4)–2.279(4) Å) The Re–C_COD_ bond lengths (2.281(4)–2.289(3) Å) are slightly longer than the corresponding Re–C_DMBD_ bonds (2.233(2)–2.251(2) Å), and the Re–N bond in [**12**]^+^ (2.0733(16) Å) is shorter than in [**10**]^+^ (2.100(4) Å). Both complexes are thus similar as [Re(η^6^-C_6_H_6_)(η^4^-1,3-cyclohexadiene)(NCCH_3_)]^+^.^23^

**Figure 7:**
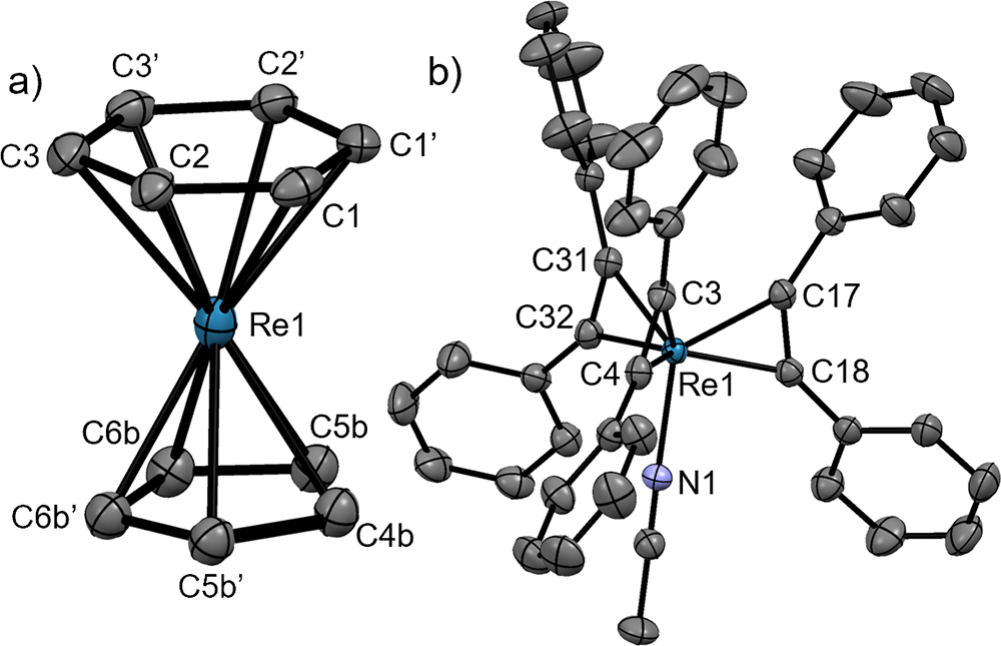
Ellipsoid displacement representation of (a) [**13**] and (b) the cation in the structure of [**14**](OTf) (ellipsoids drawn at 50% probability). For panel (b), only half of the asymmetric unit is depicted. All structural features of both species in the asymmetric unit are similar. Hydrogen atoms, (b) trifluoromethanesulfonate anion, and disorders in panel (a) are omitted for clarity. Selected bond lengths [Å]: (a) Re1–C1 2.284(6), Re1–C2 2.274(7), Re1–C3 2.277(9), Re1–C4b 2.136(18), Re1–C5b 2.162(13), Re1–C6b 2.139(15); (b) Re1–C3 2.039(3), Re1–C4 2.030(3), Re1–C17 2.040(3), Re1–C18 2.026(3), Re1–C31 2.039(3), Re1–C32 2.019(3), Re1–N1 2.128(3).

**Scheme 6:**
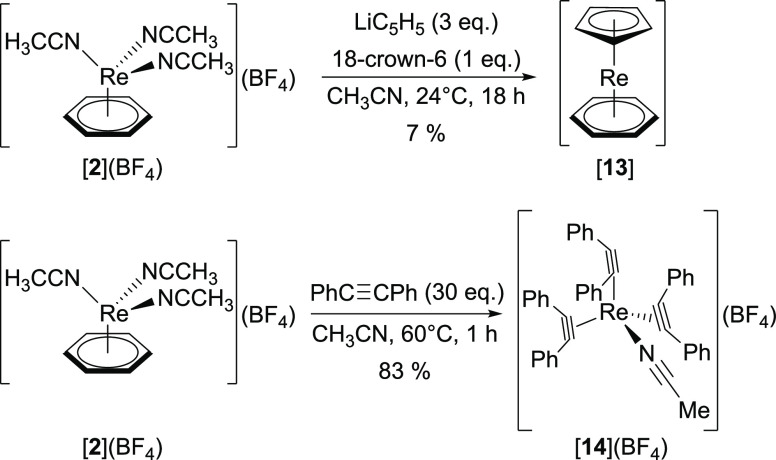
Reaction of [**2**](BF_4_) with LiC_5_H_5_ and 18-crown-6 Yields the Sandwich Complex [**13**]. Addition of the Alkyne PhC≡CPh to [**2**](BF_4_) Results in the Loss of the Benzene Ligand and Yields the Tetrahedral Complex [**14**](BF_4_)

Unexpectedly and in contrast to 1,3-cyclohexadiene, reaction of [**2**]^+^ with cyclopentadiene (a 1,3-diene) did not result in any conversion. When applying lithium cyclopentadienyl (LiC_5_H_5_) instead, the reaction of [**2**](BF_4_) with LiC_5_H_5_ (3 equiv) and 18-crown-6 (1 equiv) in CH_3_CN afforded the volatile sandwich complex [Re(η^5^-C_5_H_5_)(η^6^-C_6_H_6_)] ([**13**]) in 7% yield after purification by sublimation (Scheme 6). Complex [**13**] does not form in the absence of 18-crown-6, indicating that the activation of LiC_5_H_5_ is crucial. We note that ^1^H NMR shows the presence of co-sublimed 18-crown-6, lowering the purity of [**13**] to approximately 53%. Due to the instability of [**13**] and similar physiochemical properties, further purification of [**13**] was unsuccessful. This compound has been reported by Fischer and Wehner in 8% yield in 1968 starting from ReCl_5_ and a mixture of 1,3-cyclohexadiene, isopropyl, and cyclopentadienyl magnesium bromide in Et_2_O but without a crystal structure.^36^ Complex [**13**] crystallizes in the orthorhombic space group *Cmce* (Figure 7a). The model is highly disordered. The benzene ligand is disordered over two sets of positions (site occupancy 0.5), while the cyclopentadienyl ligands are disordered over four sets of positions (site occupancies 0.208(6) and 0.292(6)). The cyclopentadienyl ligand is more strongly bound to the Re-core (Re–C: 2.136(18)–2.17(2) Å) than the benzene ligand (Re–C: 2.274(7)–2.284(6) Å). Similar structural features were reported for the cation [Os(η^5^-C_5_H_5_)(η^6^-C_6_H_6_)]^+^ (Os–C: 2.071(9)–2.242(9) Å for the cyclopentadienyl ligand; Os–C: 2.186(7)–2.282(9) Å for the benzene ligand).^37^

So far, all substitution reactions did not result in a dissociation of the benzene ligand. Its loss was though observed when heating a mixture of [**2**](BF_4_) and diphenylacetylene (PhC≡CPh, 30 equiv) in CH_3_CN at 60 °C for 1 h, affording [Re(NCCH_3_)(η^2^-PhC≡CPh)_3_](BF_4_) ([**14**](BF_4_)) in 83% yield (Scheme 6). Tetra-coordinated alkyne complexes are known for neighboring elements, e.g., for W(0) (e.g., [W(CO)(η^2^-PhC≡CPh)_3_]).^38,39^ Examples with Re are more scarce. They are accessible from [ReX(η^2^-RC≡CR)_3_] (X = I and OSiMe_3_; R = Me and Et), obtained from a two-step procedure with [ReO(I)_3_(PPh_3_)_2_].^40,41^^1^H NMR analysis depicts two sets of signals of equal intensities for the PhC≡CPh ligands in [**14**]^+^. Thus, the three PhC≡CPh ligands are chemically equivalent, but in each of them, the phenyl moieties are in different chemical environments. This chemical inequality originates from differing proximities of the phenyl moieties and acetylenic C atoms to the NCCH_3_ ligand in [**14**]^+^.^38^ The ^13^C NMR signals of the acetylenic C atoms in [**14**]^+^ are at δ = 176.6 and 165.0 ppm (Figure S33), which are similar as in [Re(NCCH_3_)(η^2^-EtC≡CEt)_3_]^+^ (δ = 179.6 and 162.2 ppm, respectively).^41^

The distorted tetrahedral complex [**14**](OTf) was formed in the same way from [**2**](OTf) and crystallized in the monoclinic space group *P*2_1_/*c* (Figure 7b). Compared to the complexes described above, [**14**](OTf) has the longest Re–NCCH_3_ bond (2.122(3) and 2.128(3) Å; two cations in the asymmetric unit). The Re–C distances range from 2.026(3) to 2.048(3) Å. Coordination of PhC≡CPh to the Re atom leads to an elongation of the acetylenic bond (1.309(5)–1.320(5) Å in [**14**](OTf) vs 1.206(2) and 1.204(2) Å in uncoordinated PhC≡CPh) and bending of the C≡C—Ph angle (137.9(3)–146.1(3)°).^42^

## Conclusions

Fully or semi-solvated metal complexes are powerful starting materials in coordination chemistry. [Re(η^6^-C_6_H_6_)(NCCH_3_)_3_]^+^ is a novel precursor for Re arene chemistry. Its acetonitrile ligands exchange fast, thereby facilitating substitution reactions. It is remarkably stable under an ambient atmosphere. Thus, substitution reactions are performed with standard Schlenk techniques. The scope of applicable ligands is wide and entails common σ-donors such as phosphines, π-acceptors such as CO and polypyridines, or π-donors such as dienes, cyclopentadienyl, and even alkynes. Thus, we believe that [Re(η^6^-C_6_H_6_)(NCCH_3_)_3_]^+^ will pave the way for the synthesis of a plethora of novel Re arene complexes and might even be suitable for potential catalytic processes.

The CH_3_CN exchange rates of the described complexes differ substantially. While [Re(η^6^-C_6_H_6_)(NCCH_3_)_3_]^+^ or [Re(η^6^-C_6_H_6_)(bipy)(NCCH_3_)]^+^ exchanges its CH_3_CN ligands within minutes, others (e.g., [Re(η^6^-C_6_H_6_)(CO)(NCCH_3_)_2_]^+^) display almost no nitrile self-exchange. Currently, we are investigating these different nitrile exchange rates as they may enable further modification of the complex by substitution. In addition, a fast nitrile exchange results in a free coordination site on the Re^I^ atom, which is a prerequisite for a catalytic core.

## Experimental Section

### General

All reactions were performed under a N_2_ atmosphere by applying standard Schlenk techniques or by working inside an MBRAUN Labmaster DP glovebox. NMR sample preparations, crystallizations (unless otherwise stated), and KBr pellet preparation for IR spectroscopy were performed under a N_2_ atmosphere inside an MBRAUN Labmaster DP glovebox. Reagent grade chemicals from Sigma-Aldrich Chemie GmbH, Tokyo Chemical Industry, and Merck KGaA were used without further purification. NMR solvents (CD_3_CN, acetone-*d*_6_, and C_6_D_6_) were purchased from Cambridge Isotopes Laboratories, Inc. NMR solvents were dried by addition of molecular sieves (4 Å, 20 wt %).^43^ Dry diethyl ether (Et_2_O) and tetrahydrofuran (THF) were obtained from distillation over sodium. Dry toluene, dry CH_3_CN, and dry CH_2_Cl_2_ were obtained by chromatographical separation on a MB SPS system from MBRAUN. Freeze-drying was carried out with a Christ Alpha 2-4 LD plus lyophilizer. Microwave reaction vials and alumina caps with septa were purchased from Biotage. The vials were charged with the reactants inside a glovebox and sealed with the alumina caps. If necessary, the vials were heated conventionally with an oil bath. [**1**](X) (X = BF_4_ and OTf) was produced following literature procedures.^23^^1^H NMR spectra were recorded on a Bruker AV-400400 MHz spectrometer. ^13^C and ^31^P NMR spectra were proton-decoupled recorded and measured on a Bruker DRX-500500 MHz spectrometer. ^1^H and ^13^C chemical shifts were reported relative to residual protio solvent resonances.^44^^31^P chemical shifts were reported relative to an 85% H_3_PO_4_ external standard (δ 0). FT-IR spectra were measured as KBr pellets (unless otherwise stated) on a PerkinElmer Spectrum Two spectrophotometer. UV/vis spectra were collected on a Varian Cary 50 UV/visible spectrophotometer (Agilent Technologies). λ_max_ is in nanometers, and molar extinction coefficients ε are listed as log(ε). High-resolution electrospray mass spectra (HR-ESI-MS) were recorded on a maXis QTOF-MS instrument (Bruker DaltonicsGmbH, Bremen, Germany). The samples were dissolved in CH_3_CN at a concentration of ca. 50 μg/mL and analyzed via continuous flow injection (2 μL/min). The mass spectrometer was operated in the positive (and negative) electrospray ionization mode at 4000 V (−4000 V) capillary voltage, −500 V (500 V) endplate offset, with a N_2_ nebulizer pressure of 0.8 bar, and dry gas flow of 4 L min^–1^ at 180 °C. Mass spectra were acquired in the mass range from *m*/*z* 50 to 2000 at 20,000 resolution (full width at half-maximum) and 1.0 Hz rate. The mass analyzer was calibrated between *m*/*z* 118 and 2721 using an Agilent ESI-L low concentration tuning mix solution (Agilent, USA) at a resolution of 20,000 and a mass accuracy below 2 ppm. All solvents were purchased in the best LC–MS qualities. High resolution electron ionization mass spectrometry (HR-EI-MS) is as follows: double-focusing (BE geometry) magnetic sector mass spectrometer DFS (Thermo Fisher Scientific, Bremen, Germany); solid probe inlet; EI at 70 eV; source temperature 200 °C; acceleration voltage 5 kV; electric scan mode; mass range 313–345* *m*/*z* at 10,000 resolution (10% valley definition) and a scan rate of 100–200 s per decade; mass accuracy ≤2 ppm after calibration with perfluorokerosene (PFK, Fluorochem, Derbyshire, UK). Preparative HPLC on a Shimadzu HPLC system was equipped with a Dr. Maisch Reprosil C18 10 μm, 100 Å (250 × 40 mm) column. HPLC solvents were trifluoroacetic acid (0.1% in bi-distilled water) (solvent A) and acetonitrile (solvent B). Applied HPLC gradient is as follows: 0–3 min: 80% A and 20% B; 3–30 min: linear gradient from 80 to 0% A and 20 to 100% B; 30–35 min: 0% A and 100% B. The flow rate was 40 mL min^–1^. Detection was performed at 260 nm. Electrochemical measurements were carried out with a standard three-electrode setup of the glassy carbon working electrode (i.d. = 3 mm), platinum auxiliary electrode, and Ag/AgCl reference electrode. Measurements were done in acetonitrile containing (NBu_4_)(PF_6_) (0.1 M) as a conducting electrolyte. The measurements were performed first by measuring the compound alone, and in the second step, a small amount of [Fe(η^5^-C_5_H_5_)_2_] was added as an internal reference. The combined sample was then remeasured under the same conditions. All potentials were given vs Fc^+^/Fc. Sweep rate = 0.1 V/s. All crystal structures (except for [**3**](BF_4_), [**5**](BF_4_), [**12**](PF_6_), and [**13**]) were obtained by single-crystal X-ray diffraction analyses on a Rigaku OD Supernova-Atlas diffractometer equipped with an Oxford Instruments Cryojet XL cooler using a single wavelength X-ray source from a micro-focus sealed X-ray tube (Cu K_α_ radiation, λ = 1.54184 Å) or the molybdenum X-ray radiation (Mo K_α_ λ = 0.71073 Å) from a dual wavelength X-ray source. Single-crystal X-ray diffraction analyses of [**3**](BF_4_) and [**12**](PF_6_) were performed on a Rigaku OD XtaLAB Synergy, Dualflex, Pilatus diffractometer equipped with an Oxford liquid nitrogen Cryostream cooler using a single wavelength X-ray source from a micro-focus sealed X-ray tube (Cu K_α_ radiation, λ = 1.54184 Å). Single-crystal X-ray diffraction analysis of [**5**](BF_4_) was performed on a Rigaku OD Synergy/Pilatus diffractometer using the molybdenum X-ray radiation (Mo K_α_ λ = 0.71073 Å) from a dual wavelength X-ray source and an Oxford Instruments Cryojet XL cooler. Single-crystal X-ray diffraction analysis of [**13**] was performed on a Rigaku OD Synergy-HyPix diffractometer equipped with an Oxford Instruments Cryojet XL cooler using molybdenum X-ray radiation (Mo K_α_ λ = 0.71073 Å) from a dual wavelength X-ray source. Pre-experiment, data collection, data reduction, and analytical absorption correction^45^ were performed with the program suite CrysAlisPro.^46^ Using Olex2,^47^ the structure was solved with the SHELXT^48^ small molecule structure solution program and refined with the SHELXL2018/3 program package^49^ by full-matrix least-squares minimization on F^2^. The data collections and structure refinement parameters are summarized in Tables S1–S6. CCDC 2224042 for [**2**](BF_4_), 2224034 for [**3**](BF_4_), 2224033 for [**4**](BF_4_), 2224035 for [**5**](BF_4_), 2224039 for [**6**](BF_4_), 224038 for [**7**](BF_4_), 2224041 for [**8**](BF_4_), 2224037 for [**9**](BF_4_), 2224036 for [**10**](PF_6_), 2224040 for [**12**](PF_6_), 2224031 for [**13**], and 2224032 for [**14**](OTf) contain the supplementary crystallographic data for these compounds and can be obtained free of charge from the Cambridge Crystallographic Data Centre via www.ccdc.cam.ac.uk/data_request/cif.

### [Re(η^6^-C_6_H_6_)(NCCH_3_)_3_](BF_4_) ([**2**](BF_4_))

In a 5 mL microwave vial, [**1**](BF_4_) (44.4 mg, 103 μmol) was dissolved in dry CH_3_CN (3 mL) and stirred for 1 h at 60 °C. The solvent was concentrated to approximately 0.5 mL, and dry Et_2_O (5 mL) was added. The resulting suspension was centrifuged, and the supernatant was decanted. The precipitate was dried *in vacuo* to give [**2**](BF_4_) (38.0 mg, 80 μmol) in 78% yield as a greenish gray powder. Crystals suitable for X-ray diffraction analysis were obtained after slow evaporation of a CH_3_CN/toluene solution at −11 °C.

^1^H NMR (acetone-*d*_6_): 4.64 (s, C_6_H_6_), 2.74 (s, 3 × NCCH_3_) ppm. ^13^C NMR (acetone-*d*_6_): 126.0 (3 × N*C*CH_3_), 68.2 (C_6_H_6_), 3.6 (3 × NC*C*H_3_) ppm. IR: 2922 (m), 2851 (m), 2359 (m), 2341 (m), 2271 (w), 1635 (m), 1457 (m), 1436 (m), 1385 (s), 1262 (w), 1057 (s), 1024 (s), 879 (w), 806 (w). HR-ESI-MS: C_12_H_15_N_3_Re^+^ [**2**]^+^: calculated 388.08180, found 388.08277.

### [Re(η^6^-C_6_H_6_)(NCCH_3_)_2_(PPh_3_)](BF_4_) ([**3**](BF_4_))

In a 2 mL microwave tube, [**1**](BF_4_) (9.2 mg, 21 μmol) was dissolved in dry CH_3_CN (1 mL) and the yellow solution was heated to 60 °C for 1 h. PPh_3_ (8.2 mg, 31 μmol) dissolved in dry CH_3_CN (1 mL) was added to the reaction mixture. The yellow solution was stirred at 25 °C for 2.5 h before the reaction mixture was concentrated to approximately 0.4 mL with a stream of N_2_. Dry Et_2_O (3 mL) was added, and the formed yellow precipitate was collected by centrifugation. After washing with additional dry Et_2_O (3 mL) and drying with a stream of N_2_, [**3**](BF_4_) (9.8 mg, 14 μmol) was obtained in 66% yield as a yellow solid. Crystals suitable for X-ray diffraction analysis were obtained by slow evaporation of a CH_3_CN/toluene (1:1) solution.

^1^H NMR (500 MHz, CD_3_CN): 7.47 (m, 9 arom. H), 7.34–7.30 (m, 6 arom. H), 4.53 (s, C_6_H_6_), 2.19 (d, *J* = 1.3 Hz, 2 × NCCH_3_) ppm. ^13^C NMR (126 MHz, CD_3_CN): 134.6 (d, *J* = 10.7 Hz, arom. CH), 134.2 (*ipso*-C), 131.1 (arom. CH), 129.3 (d, *J* = 9.5 Hz, arom. CH), 126.1 (s, CN), 73.9 (C_6_H_6_), 4.0 (CH_3_) ppm. ^31^P NMR (202 MHz, CD_3_CN): 20.9 ppm. IR: 2926 (w), 2274 (w), 1638 (w), 1434 (m), 1384 (s), 1093 (w), 1058 (m), 882 (w), 836 (w), 750 (w), 700 (w), 620 (w), 530 (w), 514(w) cm^–1^. HR-ESI-MS: C_28_H_27_N_2_PRe^+^ [**3**]^+^: calculated 609.14639, found 609.14566.

### [Re(η^6^-C_6_H_6_)(NCCH_3_)(PPh_3_)_2_](BF_4_) ([**4**](BF_4_))

Inside a N_2_ glovebox in a 10 mL Schlenk tube, [**1**](BF_4_) (22 mg, 51 μmol) was dissolved in dry CH_3_CN (3 mL) and the yellow solution was heated to 60 °C for 1 h. PPh_3_ (105 mg, 400 μmol, 8 equiv) was added to the reaction mixture. The yellow reaction mixture was heated to 100 °C for 18 h, after which the reaction mixture was concentrated *in vacuo* to approximately 0.5 mL inside the glovebox. Dry Et_2_O (3 mL) was added, and the green-yellow supernatant was separated from the dark green precipitate by filtration. The precipitate was extracted with a CH_3_CN/Et_2_O mixture (1:6, 3.5 mL), and the combined filtrates were dried to completeness *in vacuo*. CH_2_Cl_2_ (0.5 mL) and dry Et_2_O (6 mL) were added, yielding a yellow precipitate, which was collected by filtration. The yellow precipitate was washed with a CH_2_Cl_2_/Et_2_O mixture (1:12, 2 × 6.5 mL) and Et_2_O (2 × 2 mL) before it was dried with a stream of N_2_, giving [**4**](BF_4_) (33.2 mg, 36.2 mmol) as a yellow solid in 71% yield. Crystals suitable for X-ray diffraction analysis were obtained by vapor diffusion (CH_2_Cl_2_/Et_2_O). Note: There was a small contamination of the product by [**3**]^+^ and [Re(η^6^-C_6_H_6_)_2_]^+^ (approximately 7.5% each), indicated by ^1^H NMR.

^1^H NMR (CD_3_CN): 7.36 (t, *J* = 7.3 Hz, 6 × arom. H), 7.28 (t, *J* = 7.6 Hz, 12 × arom. H), 7.15–7.10 (m, 12 × arom. H), 4.52 (s, C_6_H_6_), 2.25 (t, *J* = 1.9 Hz, CH_3_CN) ppm. ^13^C NMR (CD_3_CN): 137.2–136.7 (m, *ipso*-C), 134.6 (t, *J* = 5.0 Hz, arom. CH), 130.9 (s, arom. CH), 129.0 (t, *J* = 4.8 Hz, arom. CH), 126.9 (CH_3_*C*N), 78.5 (C_6_H_6_), 4.9 (*C*H_3_CN) ppm. ^31^P NMR (CD_3_CN): 6.3 ppm. IR: 2258 (w), 1585 (w), 1479 (w), 1433 (m), 1311 (w), 1282 (w), 1184 (w), 1087 (m), 1053 (s), 998 (m), 827 (w) cm^–1^. HR-ESI-MS: C_44_H_39_NP_2_Re^+^ [**4**]^+^: calculated 830.21098, found 830.21019.

### [Re(η^6^-C_6_H_6_)(NCCH_3_)(PMe_3_)_2_](BF_4_) ([**5**](BF_4_))

Inside a N_2_ glovebox in a 25 mL Schlenk flask, [**1**](BF_4_) (19.2 mg, 44.5 μmol) was dissolved in dry CH_3_CN (3 mL) and the yellow solution was heated to 60 °C. PMe_3_ (1 M in THF, 0.37 mL, 370 μmol) was added to the reaction mixture and heated to 60 °C for 1.5 h. Afterward, the reaction mixture was concentrated *in vacuo* to approximately 0.2 mL and dry Et_2_O (7 mL) was added. The yellow precipitate was collected by filtration and washed with a mixture of CH_3_CN/Et_2_O (1:10, 5.5 mL) and Et_2_O (2 × 2 mL). Drying with a stream of N_2_ afforded [**5**](BF_4_) (20.7 mg, 38.0 μmol) in 85% yield as a yellow solid. Crystals suitable for X-ray diffraction analysis were obtained by vapor diffusion (THF/hexane).

^1^H NMR (CD_3_CN): 4.62 (s, C_6_H_6_), 2.49 (t, *J* = 2.2 Hz, NCCH_3_), 1.59–1.57 (m, 2 × PMe_3_) ppm. ^13^C NMR (CD_3_CN): 122.9 (CH_3_*C*N), 74.2 (C_6_H_6_), 21.2–20.7 (m, PMe_3_), 4.4 (*C*H_3_CN) ppm.^31^P NMR (CD_3_CN): −40.9 ppm. IR: 2981 (w), 2916 (w), 2252 (w), 1423 (m), 1304 (w), 1286 (m), 1089 (m), 1030 (s), 936 (s), 859 (m), 824 (m), 722 (w), 672 (w), 647 (w), 615 (w) cm^–1^. HR-ESI-MS: C_14_H_27_NP_2_Re [**5**]^+^: calculated 458.11708, found 458.11681.

### [Re(η^6^-C_6_H_6_)(dppe)(NCCH_3_)](BF_4_) ([**6**](BF_4_))

Inside a glovebox in a 25 mL Schlenk flask, [**1**](BF_4_) (15.9 mg, 36.9 μmol) was dissolved in dry CH_3_CN (3 mL) and the solution was heated to 60 °C for 1 h. dppe (17.4 mg, 43.7 μmol) was added, and the mixture was heated to 60 °C for 1.5 h. The solvent was reduced *in vacuo* to approximately 0.3 mL. Upon addition of dry Et_2_O (6 mL), a yellow suspension formed. The solid was collected by filtration, washed with a CH_3_CN/Et_2_O mixture (1:12, 2 × 6.5 mL) and Et_2_O (2 × 2 mL), and dried with a stream of N_2_, affording [**6**](BF_4_) (21.3 mg, 26.9 μmol) in 73% yield. Crystals suitable for X-ray diffraction analysis were obtained by slow evaporation of a CH_3_CN/toluene (1:1) solution.

^1^H NMR (CD_3_CN): 7.75–7.71 (m, 4 arom. H), 7.50–7.40 (m, 12 arom. H), 7.32–7.28 (m, 4 arom. H), 4.57 (s, C_6_H_6_), 2.70–2.58 (m, 2 × C*H*H), 2.54–2.42 (m, 2 × CH*H*), 1.43 (s, CH_3_CN) ppm. ^13^C NMR (CD_3_CN): 139.7 (d, *J* = 48.9 Hz, *ipso*-C), 134.1 (d, *J* = 10.8 Hz, arom. CH), 131.7 (d, *J* = 10.1 Hz, arom. CH), 131.3 (s, arom. CH), 130.9 (s, arom. CH), 130.3 (dd, *J* = 44.3, 4.0 Hz, *ipso*-C), 129.7 (d, *J* = 9.9 Hz, arom. CH), 129.5 (d, *J* = 10.0 Hz, arom. CH), 123.2 (CH_3_*C*N), 76.5 (t, *J* = 2.0 Hz, C_6_H_6_), 29.8 (dd, *J* = 33.8, 12.8 Hz, CH_2_), 3.22 (CH_3_CN) ppm. ^31^P NMR (CD_3_CN): 46.9 ppm. IR: 3059 (w), 3026 (w), 2923 (m), 2851 (w), 2266 (w), 1633 (m), 1482 (w), 1435 (m), 1384 (w), 1262 (w), 1083 (s), 1056 (s), 871 (w), 805 (w), 698 (w), 674 (w), 617 (w), 530 (m) cm^–1^. HR-ESI-MS: C_34_H_33_NP_2_Re [**6**]^+^: calculated 704.16403, found 704.16224.

### [Re(η^6^-C_6_H_6_)(bipy)(NCCH_3_)](BF_4_) ([**7**](BF_4_))

Inside a glovebox in a 25 mL Schlenk flask, [**1**](BF_4_) (14.6 mg, 33.9 μmol) was dissolved in dry CH_3_CN (3 mL). The solution was heated to 60 °C for 1 h before 2,2′-bipyridine (7.0 mg, 45 μmol) was added, and the mixture was heated to 60 °C for 1.5 h, after which the solvent was reduced *in vacuo* to approximately 0.3 mL. Dry Et_2_O (7 mL) was added, and the formed purple precipitate was collected by filtration. The solid was washed with a CH_3_CN/Et_2_O mixture (1:16, 3 × 5.1 mL) and Et_2_O (2 × 2 mL) and dried with a stream of N_2_, affording [**7**](BF_4_) (15.5 mg, 28.3 mmol) in 83% yield. Crystals suitable for X-ray diffraction analysis were obtained from slow evaporation of a CH_3_CN/toluene solution at −11 °C.

^1^H NMR (CD_3_CN): 9.41 (d, *J* = 5.6 Hz, 2 arom. H), 8.37 (d, *J* = 8.3 Hz, 2 arom. H), 7.82–7.79 (m, 2 arom. H), 7.42–7.40 (m, 2 arom. H), 4.59 (s, C_6_H_6_), 2.14 (s, NCCH_3_, integral <3 due to fast CH_3_CN/CD_3_CN exchange) ppm. ^13^C NMR (CD_3_CN): 155.8 (2 × arom. C), 154.5 (2 × arom. CH), 136.3 (2 × arom. CH), 126.8 (2 × arom. CH), 124.0 (coordinated NCCD_3_), 123.4 (2 × arom. CH), 69.7 (C_6_H_6_), 3.7 (septuplet, coordinated NCCD_3_) ppm. IR: 2926 (w), 2852 (w), 2259 (w), 1633 (m), 1452 (m), 1417(w), 1384 (m), 1256 (m), 1083 (s), 1058 (s), 828 (w), 760 (m), 619 (w) cm^–1^. HR-ESI-MS: C_18_H_17_N_3_Re^+^ [**8**]^+^: calculated 462.09745, found 462.09729. UV/vis (CH_3_CN): λ_max_ 297 (4.39), λ_max_ 407 (3.63), λ_max_ 569 (3.63) nm.

### [Re(η^6^-C_6_H_6_)(phen)(NCCH_3_)](BF_4_) ([**8**](BF_4_))

Inside a glovebox in a microwave vial, [**1**](BF_4_) (18.9 mg, 44 μmol) was dissolved in dry CH_3_CN (2.5 mL). The solution was heated for 1 h at 60 °C. Afterward, 1,10-phenanthroline (9.1 mg, 50 μmol) dissolved in dry CH_3_CN (0.5 mL) was added to the solution. The mixture was stirred for 2.5 h at room temperature, then for 1 h at 60 °C, then for 25 min at 70 °C, and at the end for 10 min at 80 °C. The mixture was concentrated to approximately 0.4 mL by a stream of N_2_. Dry Et_2_O (6 mL) was added, the resulting suspension was centrifuged, and the supernatant was decanted. The precipitate was washed with dry Et_2_O (4 mL) and dried by a stream of N_2_ to afford [**8**](BF_4_) (17.9 mg, 31 μmol, 70%) as a deep purple solid. Crystals suitable for X-ray diffraction analysis were obtained by vapor diffusion (CH_2_Cl_2_/Et_2_O) at −11 °C.

^1^H NMR (CD_3_CN): 9.74 (dd, *J* = 5.4, 1.2 Hz, 2 arom. H), 8.41 (dd, *J* = 8.1, 1.2 Hz, 2 arom. H), 8.11 (s, 2 arom. H), 7.83 (dd, *J* = 8.1 and 5.4 Hz, 2 arom. H), 4.70 (s, C_6_H_6_), 2.01 (s, CH_3_CN, integral <3 due to fast CH_3_CN/CD_3_CN exchange) ppm. ^13^C NMR (CD_3_CN): 154.5 (2 × arom. CH), 147.4 (1 × arom. C), 135.6 (2 × arom. CH,), 131.1 (1 × arom. C), 128.3 (2 × arom. CH), 125.9 (2 × arom. CH), 124.4 (coordinated N*C*CD_3_) 69.2 (C_6_H_6_), 3.6 (septuplet, coordinated NC*C*D_3_) ppm. IR (neat): 3069 (w), 2937 (w), 2260 (w), 1570 (w), 1506 (w), 1427 (m), 1410 (m), 1281 (m), 1199 (m), 1045 (s), 1026 (s), 877 (w), 831 (m), 770 (w) cm^–1^. HR-ESI-MS: C_20_H_17_N_3_Re^+^ [**8**]^+^: calculated 486.09745, found 486.09744. UV/vis (CH_3_CN): λ_max_ 557 nm (3.84).

### [Re(η^6^-C_6_H_6_)(CO)(NCCH_3_)_2_](BF_4_) ([**9**](BF_4_))

Inside a glovebox, [**1**](BF_4_) (22.1 mg, 51 μmol) was dissolved in dry CH_3_CN (4 mL) in a 10 mL Schlenk tube. Outside the glovebox, the solution was heated at 60 °C for 1 h. Afterward, the N_2_ atmosphere was exchanged with CO and the mixture was heated at 60 °C for 9 h before the solvent was concentrated by a stream of N_2_ to approximately 0.5 mL. Dry Et_2_O (5 mL) was added, the formed suspension was centrifuged, and the supernatant was decanted. The solid was washed with additional dry Et_2_O (2 mL). The solid was dried by a stream of N_2_, affording [**9**](BF_4_) (21.4 mg, 46 μmol) in 90% yield as a brown solid. Crystals suitable for X-ray diffraction analysis were obtained by vapor diffusion (CH_3_CN/Et_2_O).

^1^H NMR (CD_3_CN): 5.19 (s, C_6_H_6_), 2.52 (s, 2 × NCCH_3_) ppm. ^13^C NMR (CD_3_CN): 200.5 (C≡O), 126.3 (N*C*CH_3_), 68.2 (C_6_H_6_), 15.6 (NC*C*H_3_) ppm. IR (neat): 3091 (w), 2999 (w), 2937 (w), 2286 (w), 2257 (w), 1900 (s), 1506 (w), 1424 (m), 1366 (w), 1286 (w), 1095 (m), 1052 (s), 910 (m), 858 (w), 831 (m). HR-ESI-MS: C_11_H_12_ON_2_Re^+^ [**9**]^+^: calculated 357.05017, found 357.04960.

### [Re(η^6^-C_6_H_6_)(η^4^-DMBD)(NCCH_3_)](PF_6_) ([**10**](PF_6_))

[**1**](OTf) (52.6 mg, 107 μmol) was dissolved in dry CH_3_CN (5 mL) and heated to 60 °C for 1 h. Afterward, 2,3-dimethyl-1,3-butadiene (DMBD, 360 μL, 3.20 mmol) was added to the yellow-greenish solution. After heating the solution at 60 °C for an additional 1.5 h, the solvent was removed by a stream of N_2_. The orange residue was purified by preparative HPLC. The solvent was removed by freeze-drying. The obtained orange solid was suspended in sat. aq. (NH_4_)(PF_6_) (3 mL) and extracted with CH_2_Cl_2_ (2 × 2 mL). The organic extract was washed with H_2_O (2 × 2 mL), and the solvent was removed with a stream of N_2_. Recrystallization of the orange solid (vapor diffusion, acetone/Et_2_O) gave [**10**](PF_6_) (37.5 mg, 70.4 μmol) in 66% yield as orange crystals suitable for X-ray diffraction analysis.

^1^H NMR (acetone-*d*_6_): 5.31 (s, C_6_H_6_), 3.77 (s, 2 × =C*H*H), 2.71 (s, CH_3_CN), 2.18 (s, 2 × CH_3_), 1.46 (s, 2 × =CH*H*) ppm. ^13^C NMR (acetone-*d*_6_): 132.8 (N*C*CH_3_), 102.6 (2 × *C*=CH_2_), 83.5 (C_6_H_6_), 46.5 (2 × =CH_2_), 19.6 (2 × CH_3_), 4.6 (NC*C*H_3_) ppm. IR: 3066 (w), 2998 (w), 2932 (w), 2321 (w), 1444 (w), 1420 (m), 1404 (w), 1384 (w), 1263 (s), 1225 (m), 1169 (m), 1145 (m), 1028 (s), 907 (w), 822 (w), 638 (s). HR-ESI-MS: C_14_H_19_NRe^+^ [**10**]^+^: calculated 388.10695, found 388.10643.

### [Re(η^6^-C_6_H_6_)(η^4^-NBD)(NCCH_3_)](PF_6_) ([**11**](PF_6_))

[**1**](OTf) (29.9 mg, 69.4 μmol) was dissolved in dry CH_3_CN (3 mL), and the solution was heated to 60 °C for 1 h. Norbornadiene (210 μL, 2.07 mmol) was added to the greenish-yellow solution. The mixture was heated at 60 °C for 1 h before the solvent was removed with a stream of N_2_. The greenish residue was purified by preparative HPLC, fractions containing the same product were combined, and the solvent was removed by freeze-drying. The obtained brown oil was suspended in sat. aq. (NH_4_)(PF_6_) (3 mL) and extracted with CH_2_Cl_2_ (2 × 2 mL). The organic extract was washed with H_2_O (2 × 2 mL), and the solvent was evaporated. Recrystallization of the yellow solid (vapor diffusion, acetone/Et_2_O) gave [**11**](PF_6_) (13.8 mg, 25.4 μmol) in 36% yield as yellow needles.

^1^H NMR (acetone-*d*_6_): 5.35 (t, *J* = 4.3 Hz, 2 × *H*C=CH), 5.12 (s, C_6_H_6_), 4.13 (br. s, 1 × CH), 3.81 (t, *J* = 4.0 Hz, 2 × HC=C*H*), 3.76 (br. s, 1 × CH), 2.84 (s, NCCH_3_), 0.87 (m, CH_2_) ppm. ^13^C NMR (acetone-*d*_6_): 127.5 (N*C*CH_3_), 81.6 (C_6_H_6_), 65.9 (CH_2_), 61.5 (2 × H*C*=CH), 51.4 (1 × CH), 51.1 (1 × CH), 50.9 (2 × HC=*C*H), 4.6 (NC*C*H_3_) ppm. IR: 3090 (w), 2958 (w), 2926 (w), 2857 (w), 2291 (w), 1435 (w), 1423 (w), 1406 (w), 1305 (w), 1186 (w), 1075 (w), 838 (vs), 558 (m) cm^–1^. HR-ESI-MS: C_15_H_17_NRe^+^ [**11**]^+^: calculated 398.09130, found 398.09101.

### [Re(η^6^-C_6_H_6_)(η^4^-COD)(NCCH_3_)](PF_6_) ([**12**](PF_6_))

[**1**](OTf) (22.5 mg, 45.6 μmol) was dissolved in dry CH_3_CN (1 mL), and the solution was heated to 60 °C for 1 h. Afterward, 1,5-cyclooctadiene (COD, 170 μL, 1.38 mmol) was added to the yellow-greenish solution. After heating at 60 °C for 1 h, the solvent was removed by a stream of N_2_. The greenish residue was purified by preparative HPLC, fractions containing the same product were combined, and the solvent was removed by freeze-drying. The obtained yellow solid was suspended in sat. aq. (NH_4_)(PF_6_) (3 mL) and extracted with CH_2_Cl_2_ (2 × 2 mL). The organic extract was washed with H_2_O (2 × 2 mL), and the solvent was removed with a stream of N_2_. Recrystallization of the yellow solid (vapor diffusion, acetone/Et_2_O) afforded [**12**](PF_6_) (12.2 mg, 21.8 μmol) in 48% yield as yellow needles suitable for X-ray diffraction analysis.

^1^H-NMR (acetone-*d*_6_): 5.96–5.91 (m, 2 × *H*C=CH), 5.24 (s, C_6_H_6_), 3.72–3.68 (m, 2 × HC=C*H*), 2.76 (s, NCCH_3_); 2.59–2.40 (m, 2 × CH_2_); 2.13–1.95 (m, 2 × CH_2_) ppm. ^13^C-NMR (*d*_6_-acetone): 127.7 (N*C*CH_3_), 83.4 (C_6_H_6_), 78.3 (2 × H*C*=CH), 77.7 (2 × HC=*C*H), 33.2 (2 × CH_2_), 31.2 (2 × CH_2_), 4.4 (NC*C*H_3_) ppm. IR: 3107 (w), 2990 (w), 2929 (w), 2849 (w), 2281 (w), 1455 (w), 1430 (w), 1384 (w), 1336 (w), 1323 (w), 1247 (w), 1166 (w), 1009 (w), 838 (vs), 558 (m) cm^–1^. HR-ESI-MS: C_16_H_21_NRe [**12**]^+^: calculated 414.12260, found 414.12276.

### [Re(η^5^-C_5_H_5_)(η^6^-C_6_H_6_)] ([**13**])

Inside a glovebox, [**1**](BF_4_) (23.7 mg, 55 μmol) was dissolved in CH_3_CN (3 mL) in a 50 mL Schlenk flask and heated to 60 °C for 1 h. Afterward, Li(C_5_H_5_) (12.4 mg, 172 μmol) dissolved in CH_3_CN (2 mL) and 18-crown-6 (14.6 mg, 55 μmol) dissolved in CH_3_CN (1.5 mL) were added to the reaction solution. The reaction mixture was stirred for 18 h at 24 °C. The solution was transferred to a 50 mL round bottom flask, and the solvent was removed by a stream of N_2_ to ensure slow evaporation. Purification was performed by sublimation (35 °C, 0.35 mbar), affording [**13**] (1.4 mg, 4 μmol) in 7% yield as yellow-orange needles. Crystals suitable for X-ray diffraction analysis were obtained by slow evaporation of a cyclohexane solution. Note: The obtained product was contaminated by co-sublimed 18-crown-6 (^1^H NMR estimated purity of [**13**] = 53%).

^1^H NMR (C_6_D_6_): 4.98 (s, C_5_H_5_), 4.66 (s, C_6_H_6_) ppm. ^13^C NMR (C_6_D_6_): 71.9 (C_5_H_5_), 60.4 (C_6_H_6_) ppm. IR: 2924 (s), 2853 (m), 1631 (m), 1458 (w), 1384 (m), 1262 (w), 1099 (m), 1023 (w), 878 (w), 812 (w), 619 (w) cm^–1^. HR-EI-MS: C_11_H_11_Re^+·^ [**13**]^+·^: calculated 330.04128, found 330.04099.

### [Re(NCCH_3_)(η^2^-PhC≡CPh)_3_](BF_4_) ([**14**](BF_4_))

In a 25 mL Schlenk flask, [**1**](BF_4_) (21.4 mg, 49.6 μmol) was dissolved in CH_3_CN (3 mL) and the solution was heated to 60 °C for 1 h before diphenylacetylene (PhC≡CPh, 0.26 g, 1.46 mmol) was added. The deep red solution was heated to 60 °C for 1 h, after which the solvent was removed *in vacuo*. Inside a glovebox, the solid was dissolved in CH_2_Cl_2_ (0.5 mL) and pentane (5 mL) was added. The precipitate was collected by filtration, washed with pentane (2 × 5 mL), and dried with a stream of N_2_, affording [**14**](BF_4_) (34.8 mg, 41.0 μmol) in 83% yield as a brown solid.

[**14**](OTf) was isolated the same way as [**14**](BF_4_), starting from [**1**](OTf). However, smaller amounts of PhC≡CPh were applied (3 equiv instead of 30 equiv) and the [**2**]^+^/alkyne mixture was heated at 60 °C for 3 h instead of 1 h. Crystals suitable for X-ray diffraction analysis were obtained by layering a concentrated Et_2_O solution of [**14**](OTf) with pentane in a standard NMR tube.

^1^H NMR ([**14**](OTf), acetone-*d*_6_): 7.69–7.67 (m, 6 × CH_ortho_), 7.62–7.59 (m, 6 × CH_meta_), 7.55–7.49 (m, 6 × CH_para_), 7.47–7.45 (m, 6 × CH_ortho_), 7.40–7.37 (m, 6 × CH_meta_), 3.06 (s, NCCH_3_) ppm. ^13^C NMR ([**14**](OTf), acetone-*d*_6_): 176.6 (3 × *C*≡C), 165.0 (3 × C≡*C*), 139.6 (N*C*CH_3_), 136.4 (3 × C_ipso_), 131.0 (3 × CH_para_), 130.4 (3 × CH_para_), 129.9 (3 × CH_ortho_), 129.6 (3 × CH_ortho_), 129.2 (3 × CH_meta_), 128.5 (3 × CH_meta_), 4.2 (1 × NC*C*H_3_) ppm. Note: Only one C_ipso_^13^C NMR signal was detected. IR ([**14**](BF_4_)): 3056 (w), 2925 (w), 2852 (w), 2320w, 2292 (w), 2218 (w), 1694 (w), 1643 (w), 1593 (w), 1573 (w), 1480 (w), 1441 (m), 1385 (w), 1315 (w), 1274 (w), 1178 (w), 1159 (w), 1056 (s), 998 (w), 931 (m), 765 (s), 691 (s), 595 (w), 580 (w), 554 (w), 515 (w). HR-ESI-MS: C_44_H_33_NRe^+^ [**14**]^+^: calculated 762.21650, found: 762.21666.

## Supporting Information Available

NMR data of new compounds, electrochemistry data, and crystallographic details (PDF)
