# Supplementary material for: Exploring Rhenium Arene Piano-Stool Chemistry with [Re(η6-C6H6)(NCCH3)3]+: A Powerful Semi-Solvated Precursor
Source: Inorg Chem. 2023 Feb 28;62(10):4227–37. doi: 10.1021/acs.inorgchem.2c04346 (PMC10015454; doi:10.1021/acs.inorgchem.2c04346)
Supplement: Supplementary file 1 — ic2c04346_si_001.pdf [file ic2c04346_si_001.pdf]

Exploring Rhenium Arene Piano-Stool Chemistry with $[Re(\eta^6\text{-}C_6H_6)(NCCH_3)_3]^+$ – A Powerful Semi-Solvated Precursor

Robin Bolliger, Lukas Siebenmann, Emily Wolf, Megan Ross, Giuseppe Meola, Olivier Blacque, Henrik Braband, and Roger Alberto*

Department of Chemistry, University of Zürich, Winterthurerstrasse 190, CH-8057 Zürich Switzerland

*Corresponding author email address: ariel@chem.uzh.ch

Supporting Information

Table of Content

1 NMR Data of Complexes 2

2 NMR Data of Ligands 19

3 Electrochemistry 21

4 UV-Vis Spectroscopy 22

5 Crystallographic Details 23

1 NMR Data of Complexes

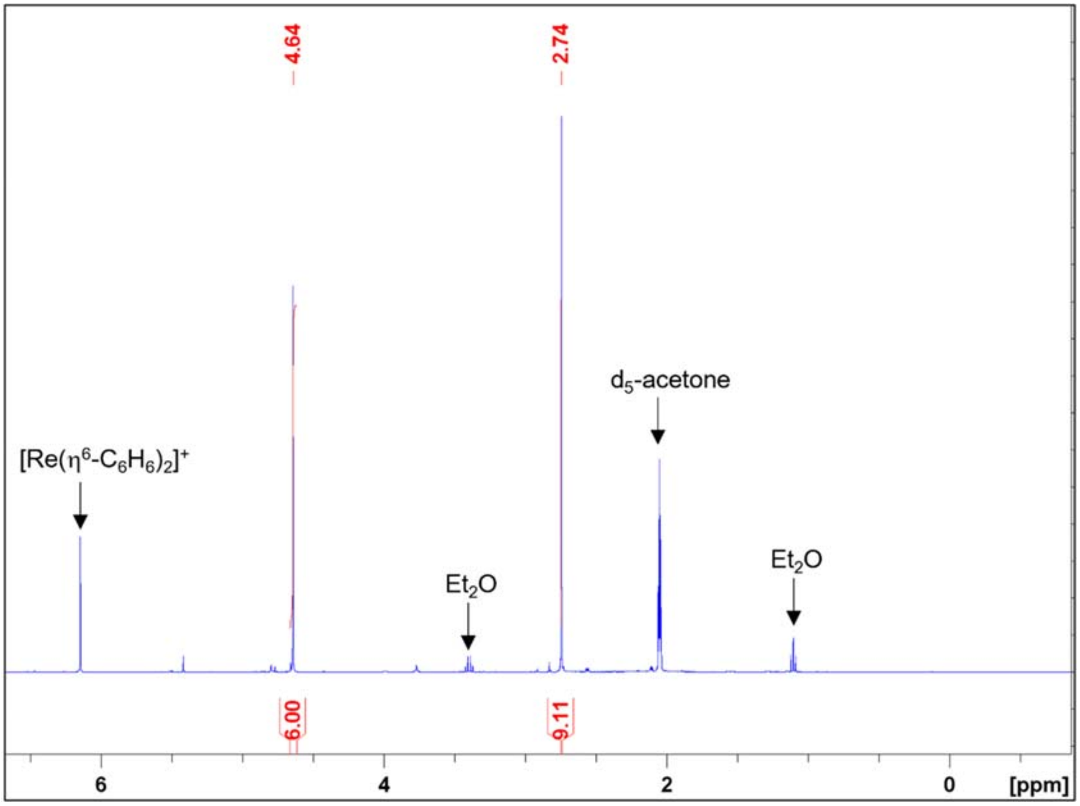

Figure S1: ^{1}H NMR spectrum of $[Re(\eta^6\text{-}C_6H_6)(NCCH_3)_3](BF_4)$ ([**2**](BF_4)) in d_6-acetone.

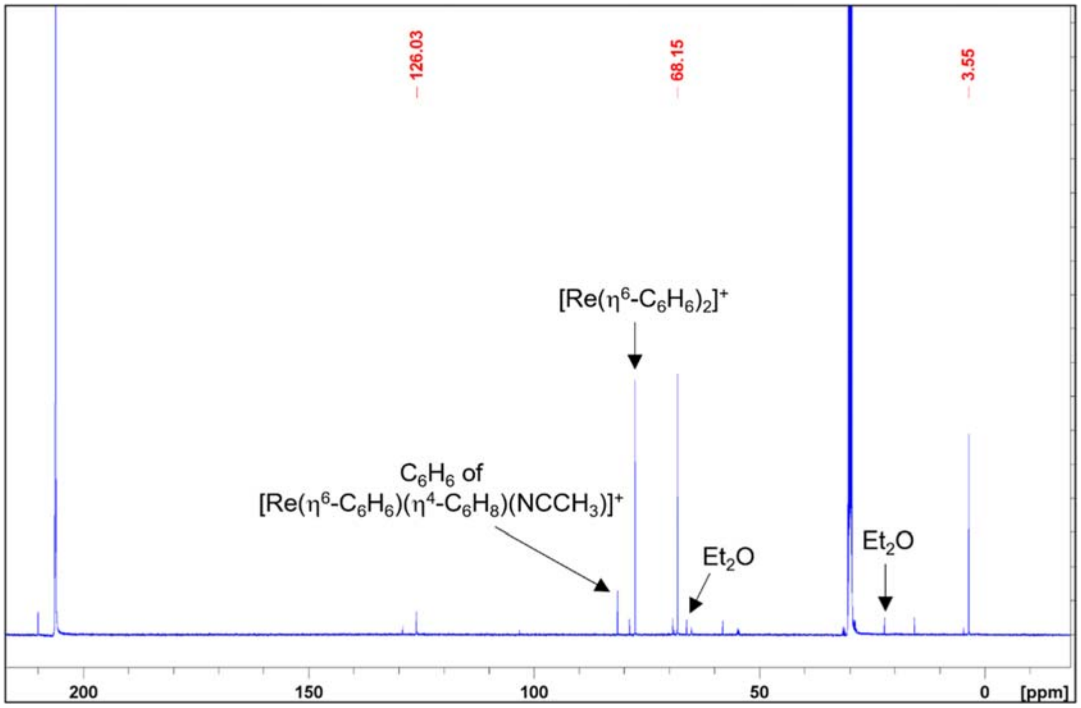

Figure S2: ^{13}C NMR spectrum of $[Re(\eta^6\text{-}C_6H_6)(NCCH_3)_3](BF_4)$ ([**2**](BF_4)) in d_6-acetone.

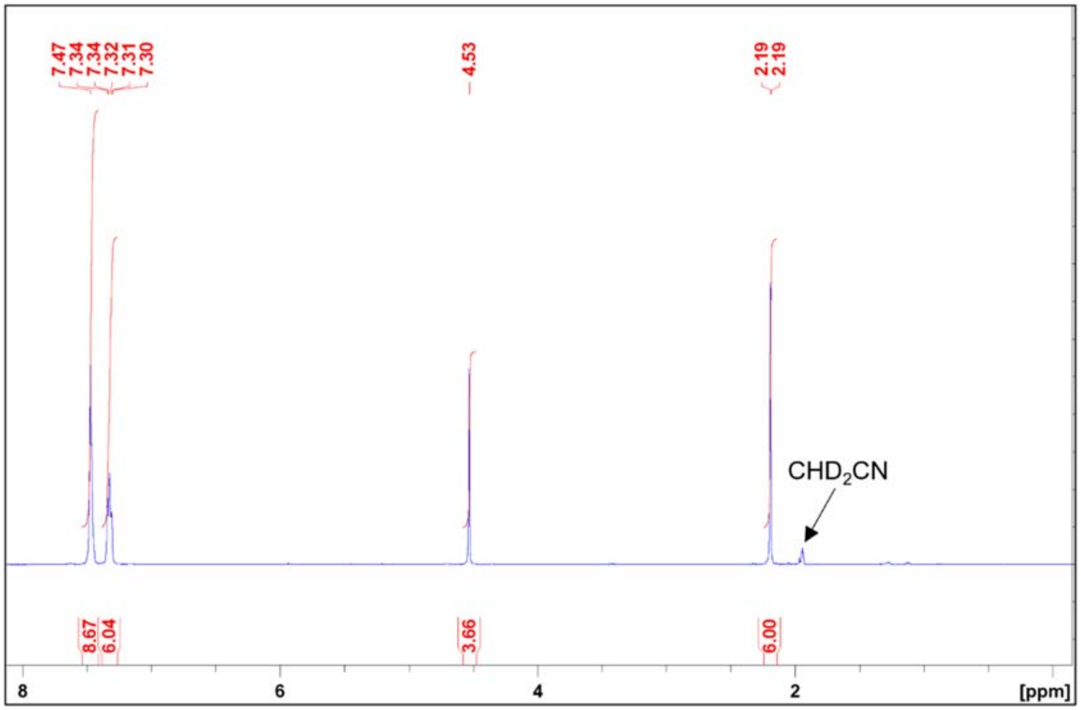

Figure S3: ^{1}H NMR spectrum of [Re(η^6-C_6H_6)($NCCH_3$)$_2$(PPh_3)](BF_4) ([**3**](BF_4)) in CD_3CN.

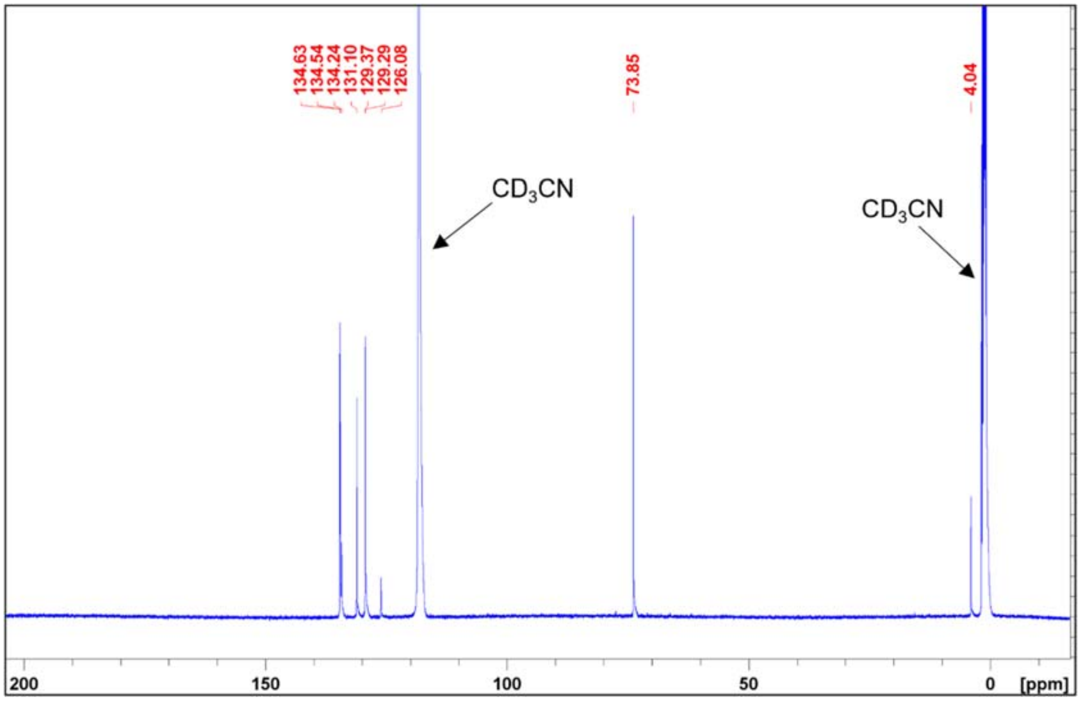

Figure S4: ^{13}C NMR spectrum of [Re(η^6-C_6H_6)($NCCH_3$)$_2$(PPh_3)](BF_4) ([**3**](BF_4)) in CD_3CN.

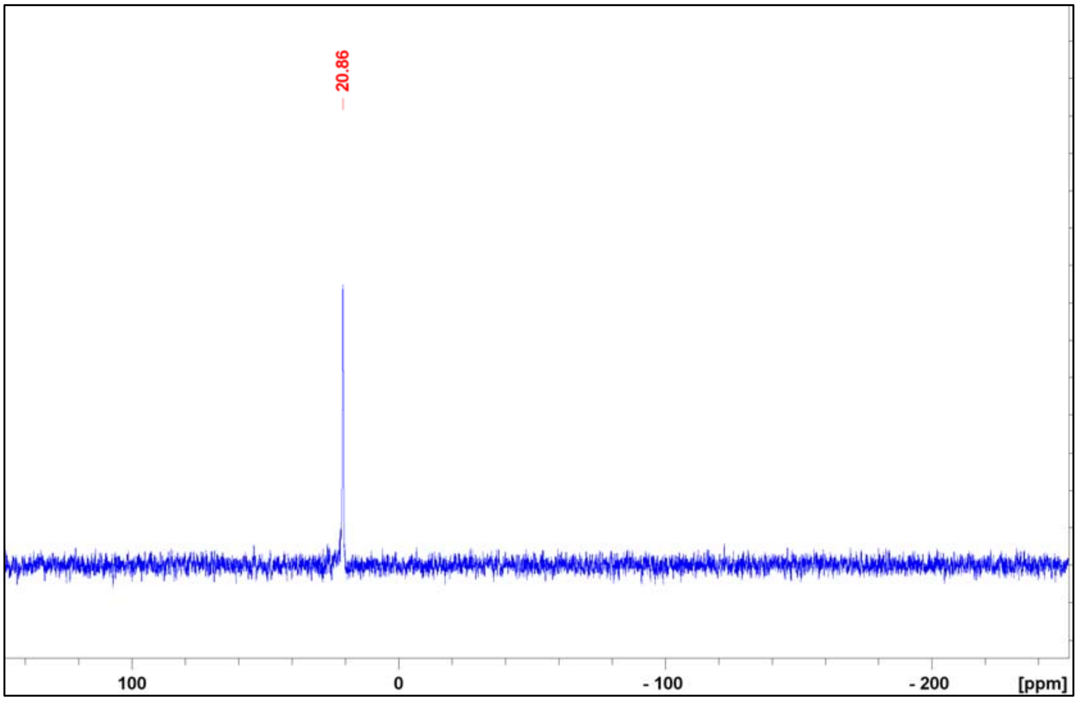

Figure S5: ^{31}P NMR spectrum of $[Re(\eta^6\text{-}C_6H_6)(NCCH_3)_2(PPh_3)](BF_4)$ ([**3**](BF_4)) in CD_3CN.

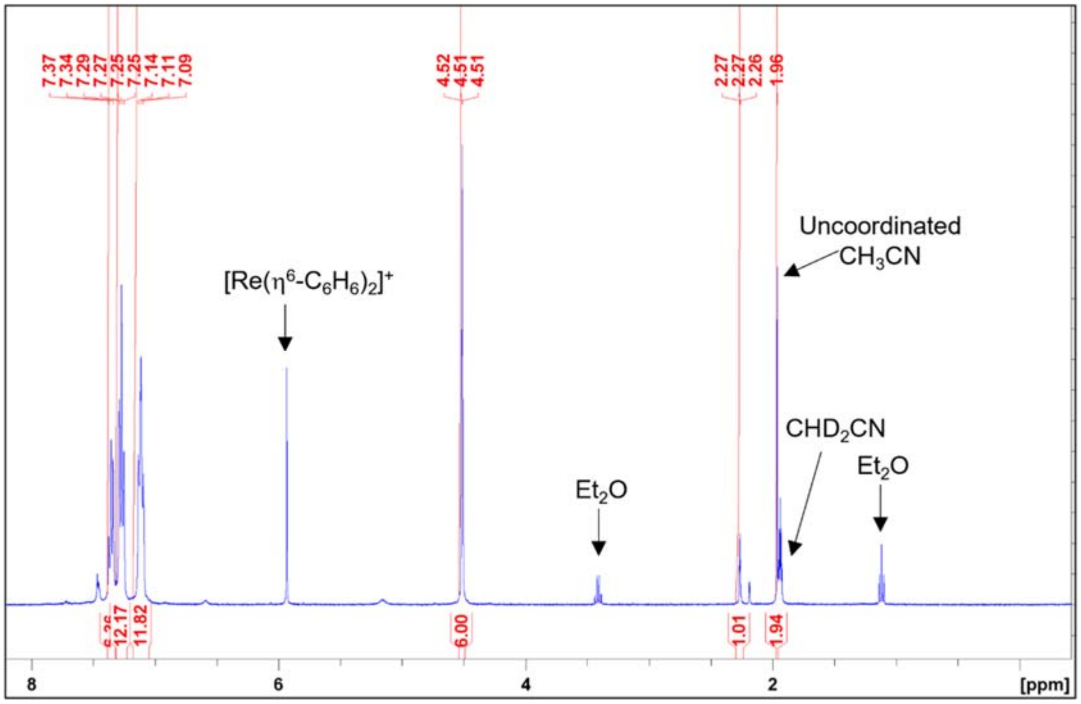

Figure S6: ^{1}H NMR spectrum of $[Re(\eta^6\text{-}C_6H_6)(NCCH_3)(PPh_3)_2](BF_4)$ ([**4**](BF_4)) in CD_3CN. Note: Due to the fast CH_3CN/CD_3CN exchange the integral of coordinated CH_3CN (δ = 2.27 ppm) is smaller than 3.

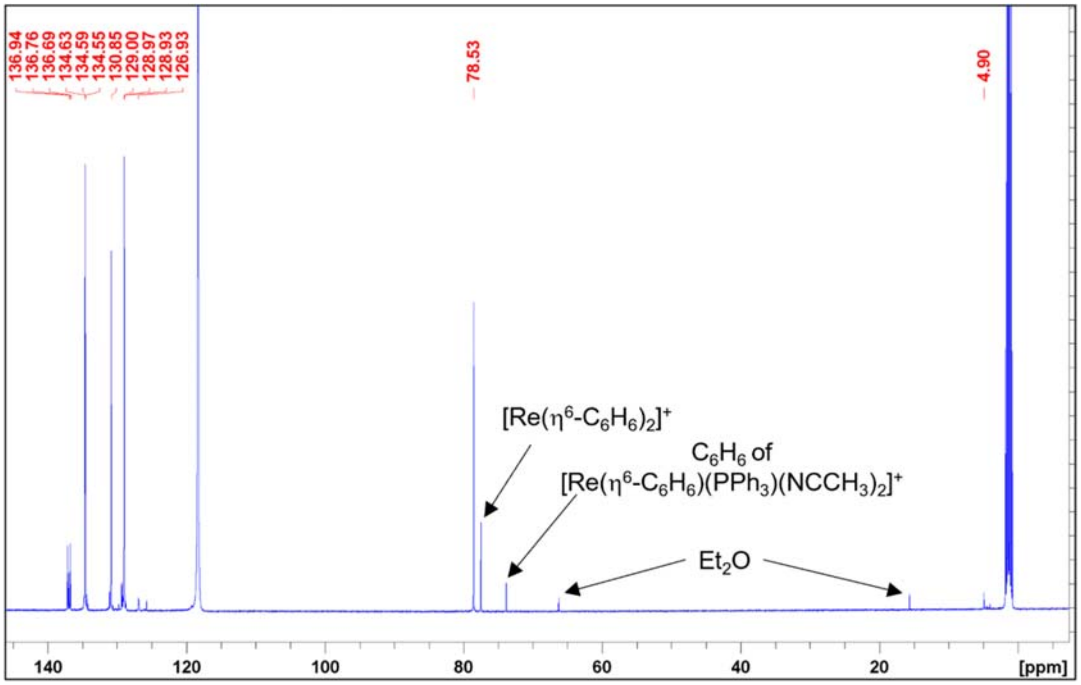

Figure S7: ^{13}C NMR spectrum of $[Re(\eta^6\text{-}C_6H_6)(NCCH_3)(PPh_3)_2](BF_4)$ ([**4**](BF_4)) in CD_3CN.

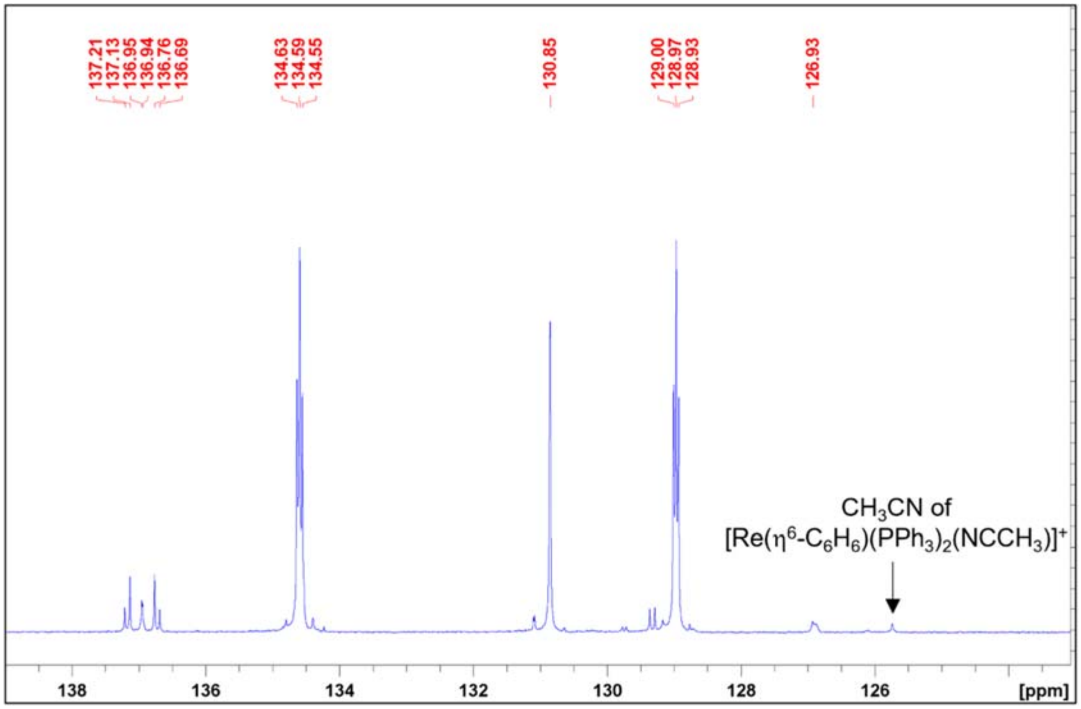

Figure S8: Detailed view on the ^{13}C NMR spectrum of $[Re(\eta^6\text{-}C_6H_6)(NCCH_3)(PPh_3)_2](BF_4)$ ([**4**](BF_4)) in CD_3CN (δ = 127-139 ppm).

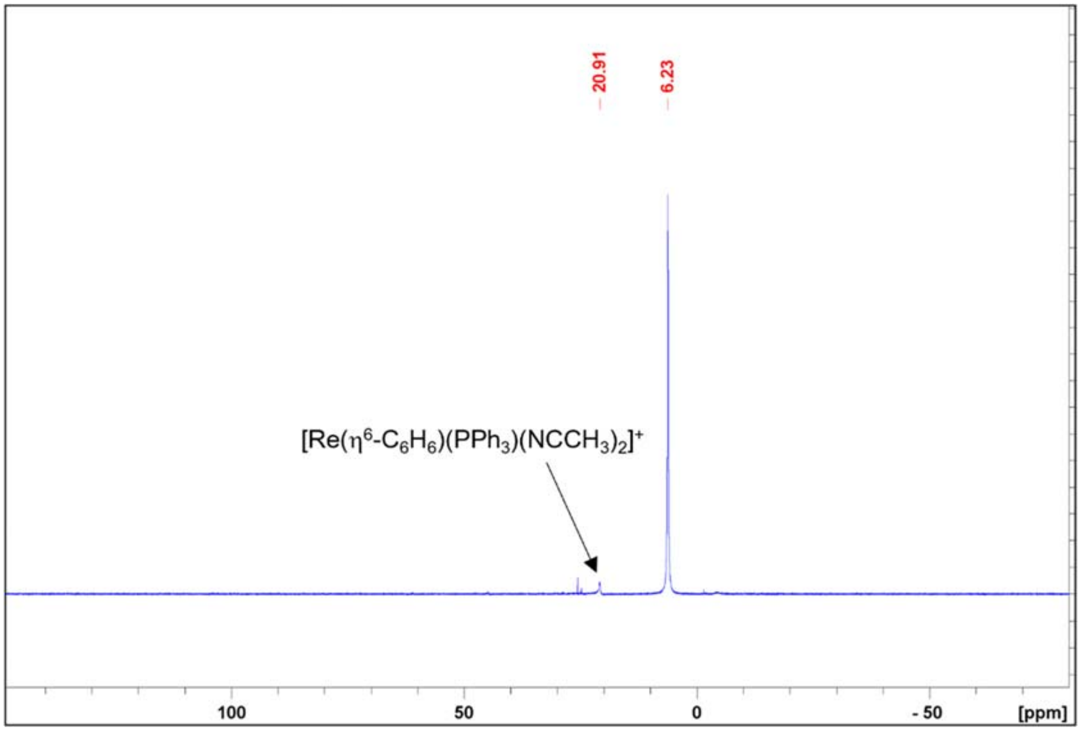

Figure S9: ^{31}P NMR spectrum of $[Re(\eta^6-C_6H_6)(NCCH_3)(PPh_3)_2](BF_4)$ ([**4**](BF_4)) in CD_3CN.

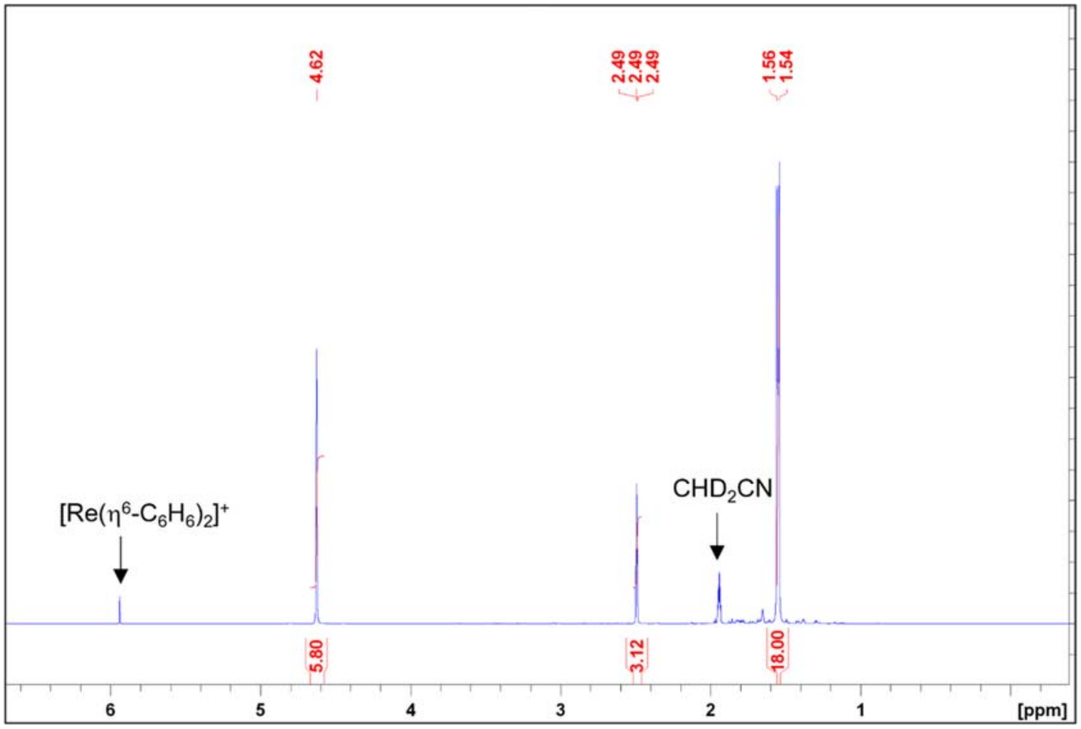

Figure S10: ^{1}H NMR spectrum of $[Re(\eta^6-C_6H_6)(NCCH_3)(PMe_3)_2](BF_4)$ ([**5**](BF_4)) in CD_3CN.

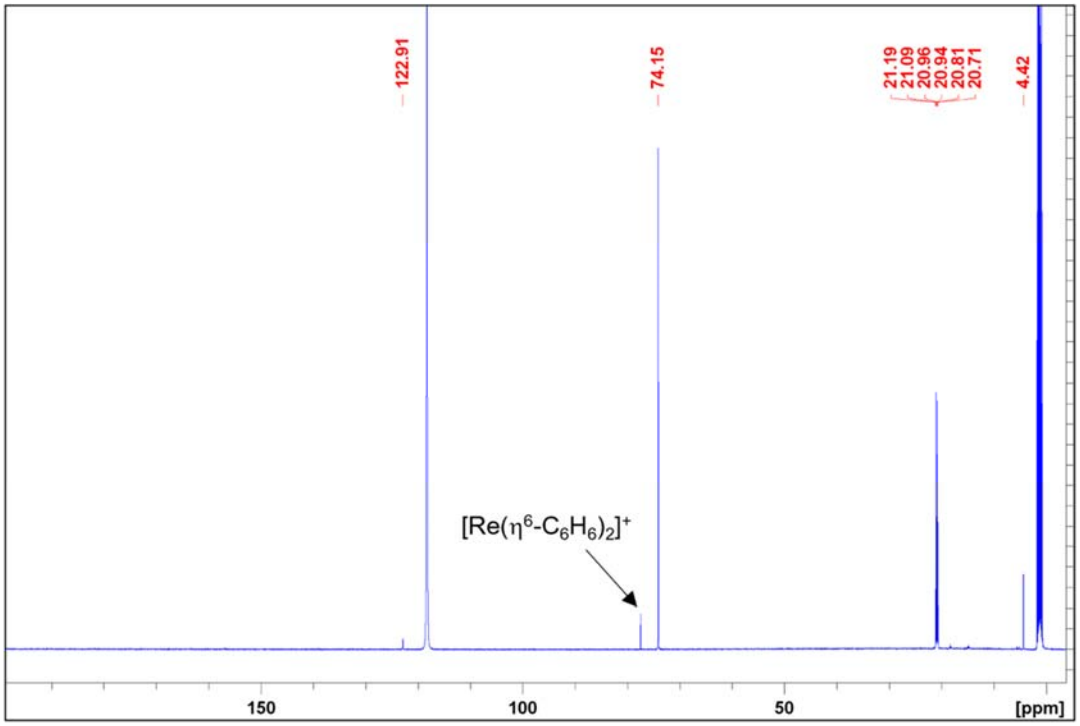

Figure S11: ^{13}C NMR spectrum of [Re(η^6-C_6H_6)($NCCH_3$)(PMe_3)$_2$](BF_4) ([**5**](BF_4)) in CD_3CN.

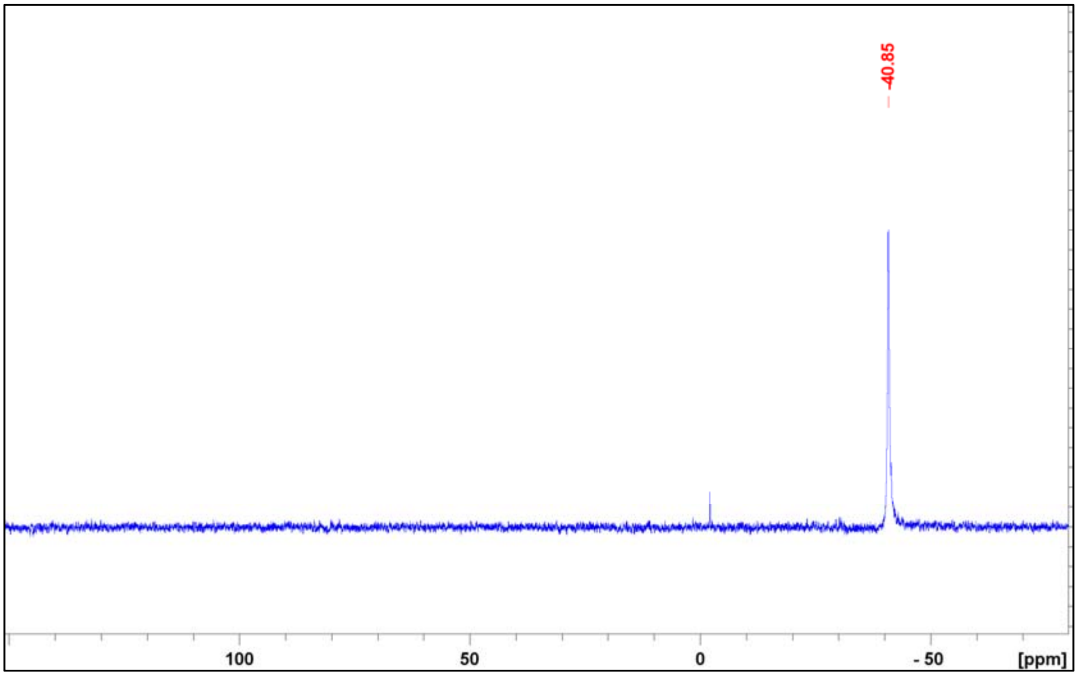

Figure S12: ^{31}P NMR spectrum of [Re(η^6-C_6H_6)($NCCH_3$)(PMe_3)$_2$](BF_4) ([**5**](BF_4)) in CD_3CN.

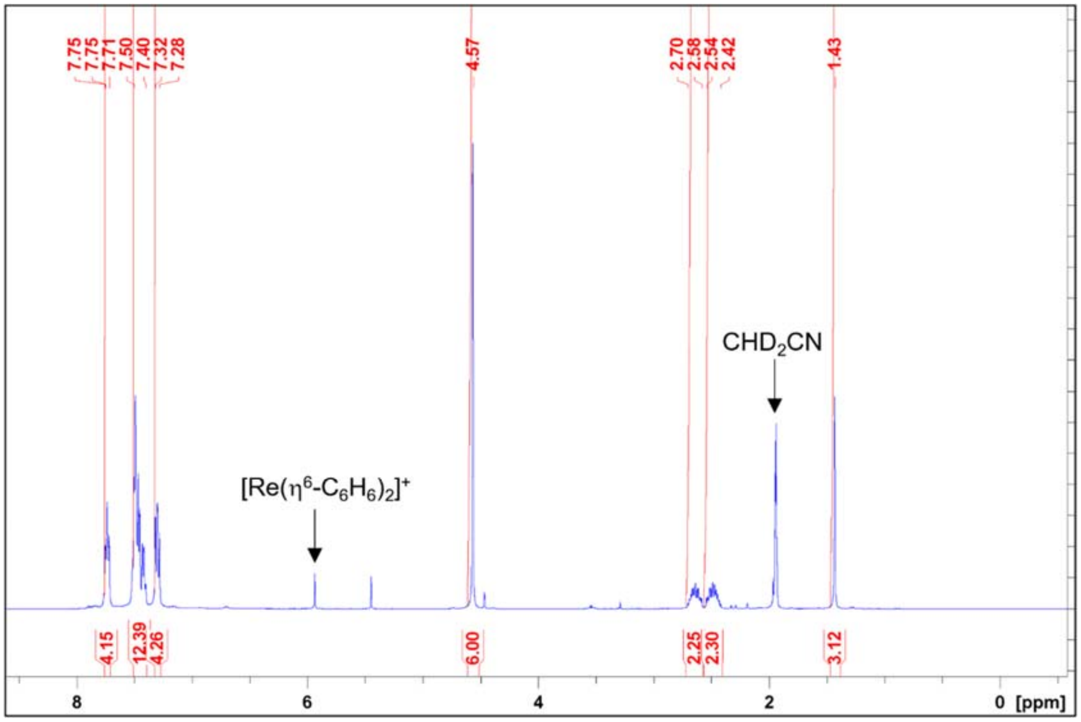

Figure S13: ^{1}H NMR spectrum of [Re(η^6-C_6H_6)(dppe)($NCCH_3$)](BF_4) ([**6**](BF_4)) in CD_3CN.

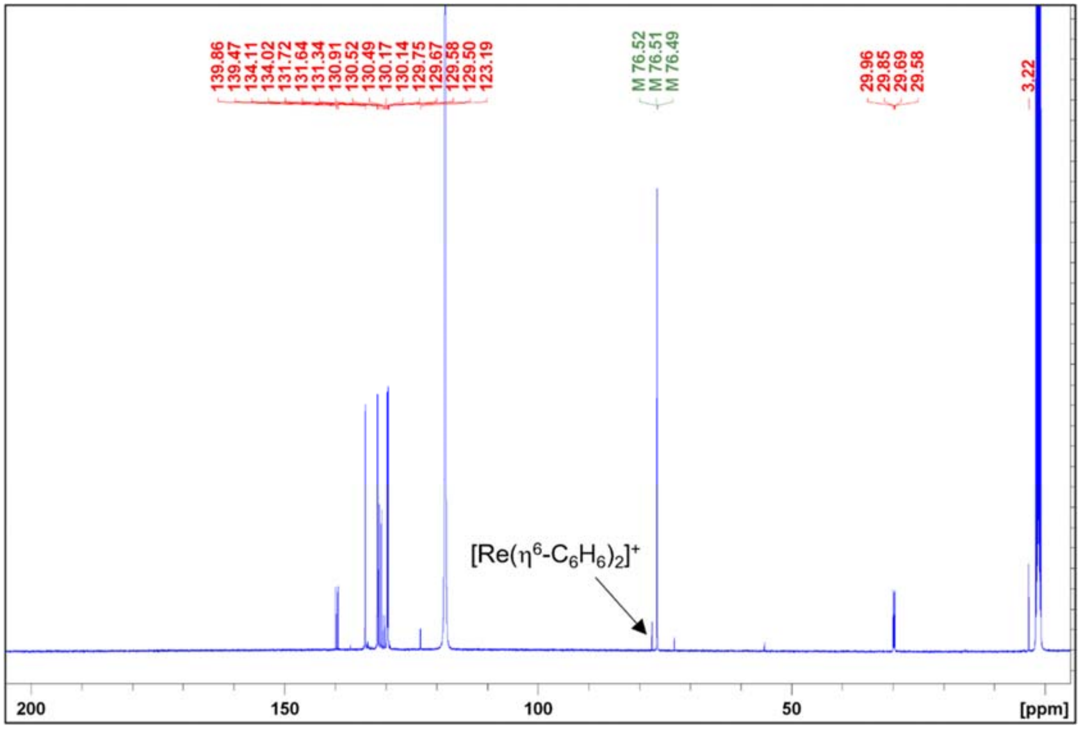

Figure S14: ^{13}C NMR spectrum of [Re(η^6-C_6H_6)(dppe)($NCCH_3$)](BF_4) ([**6**](BF_4)) in CD_3CN.

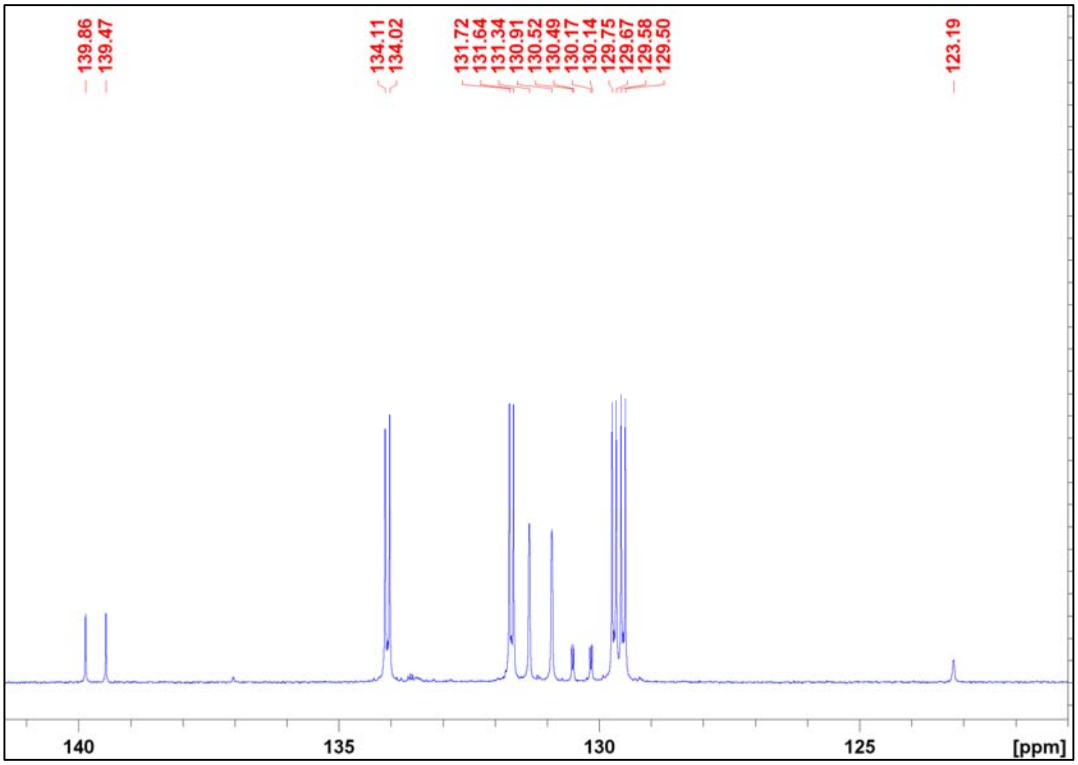

Figure S15: Detailed view on the ^{13}C NMR spectrum of [Re(η^6-C_6H_6)(dppe)($NCCH_3$)](BF_4) ([**6**](BF_4)) in CD_3CN (δ = 121 – 141 ppm).

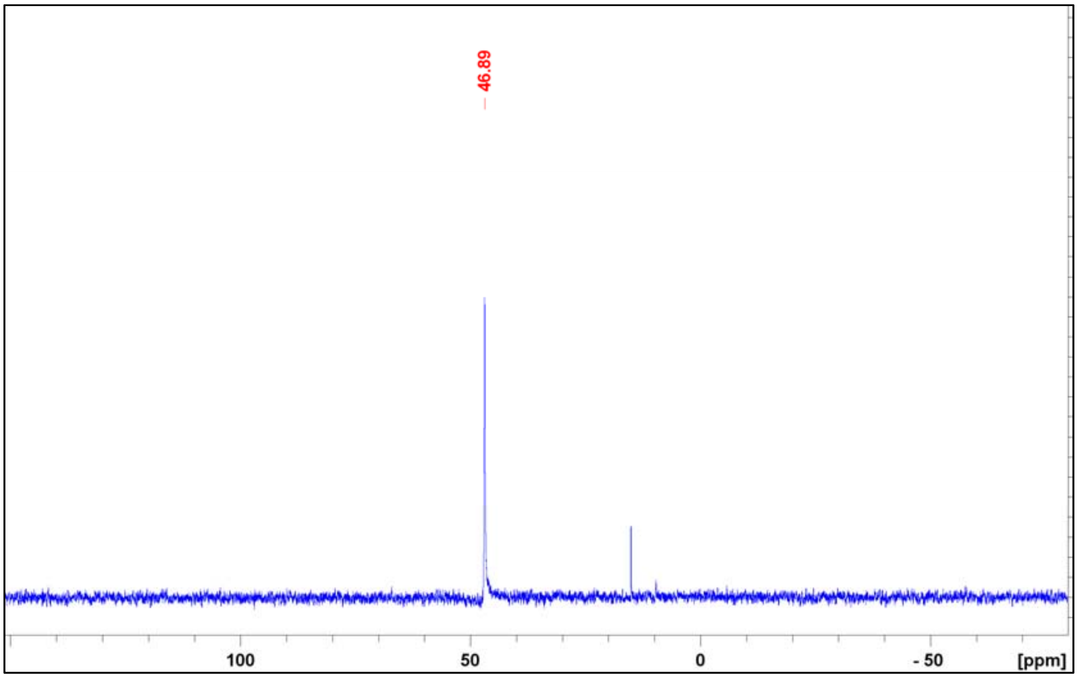

Figure S16: ^{31}P NMR spectrum of [Re(η^6-C_6H_6)(dppe)($NCCH_3$)](BF_4) ([**6**](BF_4)) in CD_3CN.

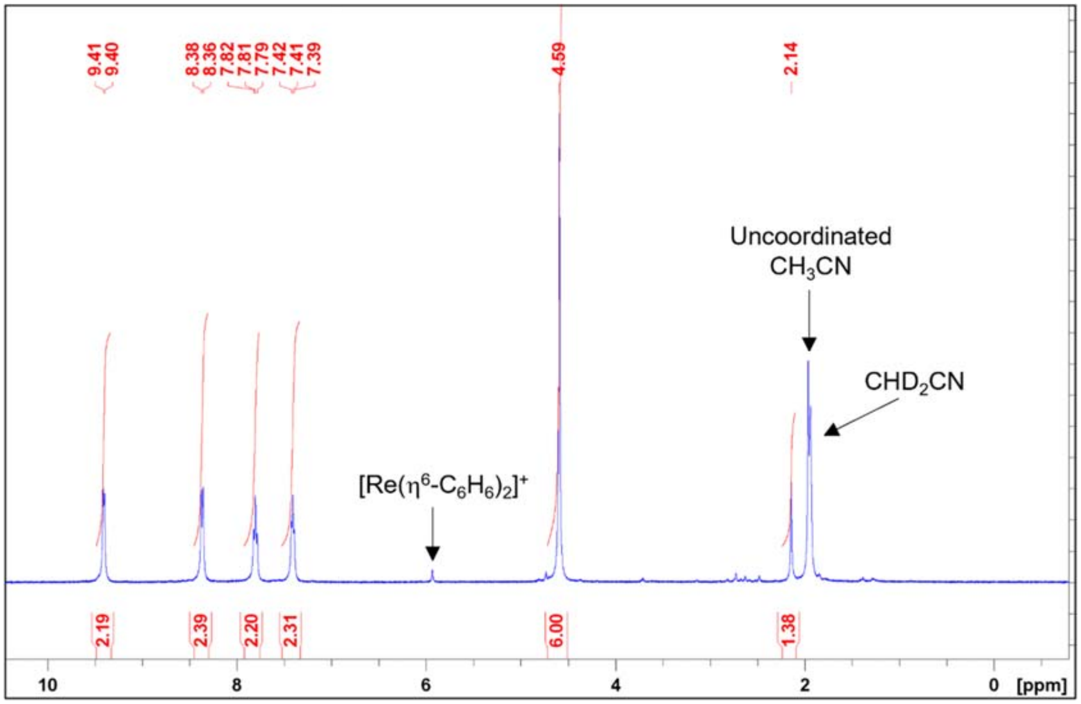

Figure S17: ^{1}H NMR spectrum of $[Re(\eta^6-C_6H_6)(bipy)(NCCH_3)](BF_4)$ ([**7**](BF_4)) in CD_3CN. Note: Due to the fast CH_3CN/CD_3CN exchange the integral of coordinated CH_3CN (δ = 2.01 ppm) is smaller than 3.

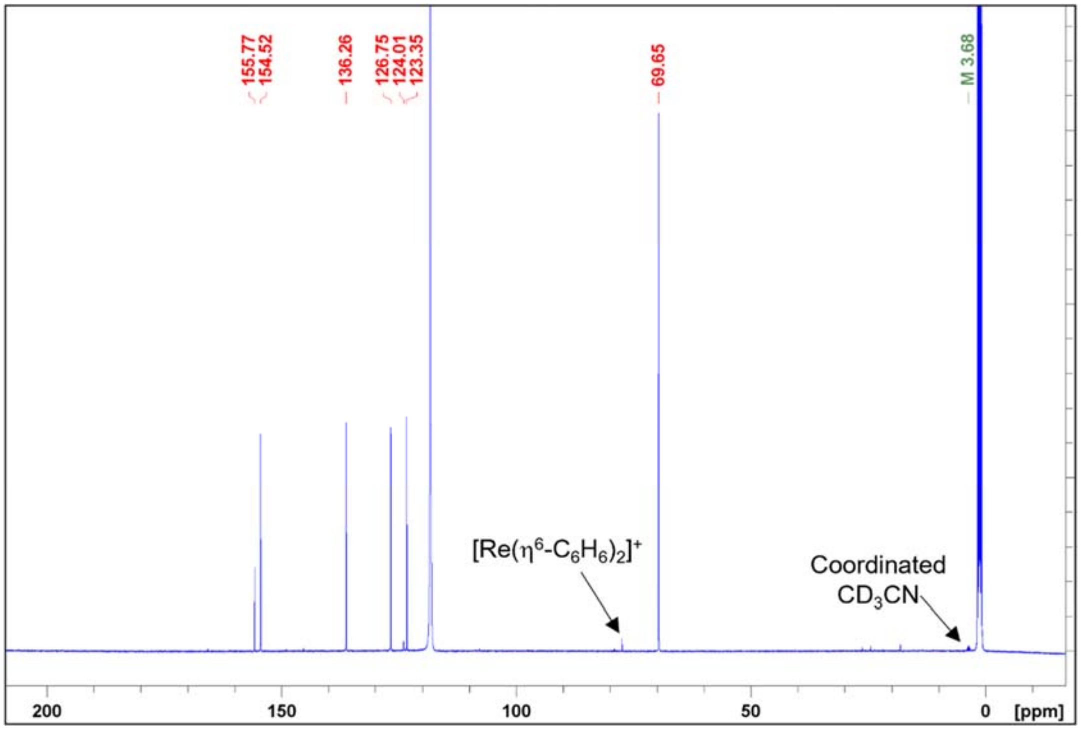

Figure S18: ^{13}C NMR spectrum of $[Re(\eta^6-C_6H_6)(bipy)(NCCH_3)](BF_4)$ ([**7**](BF_4)) in CD_3CN.

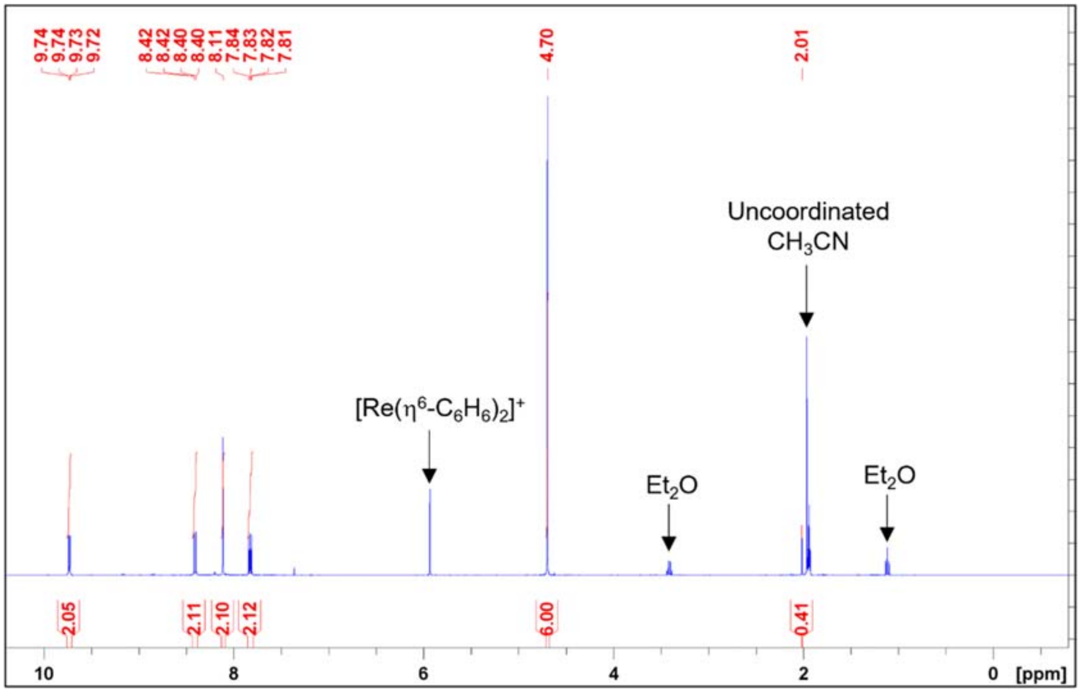

Figure S19: ^{1}H NMR spectrum of [Re(η^{6}-C_6H_6)(phen)($NCCH_3$)](BF_4) ([**8**](BF_4)) in CD_3CN. Note: Due to the fast CH_3CN/CD_3CN exchange the integral of coordinated CH_3CN (δ = 2.01 ppm) is smaller than 3.

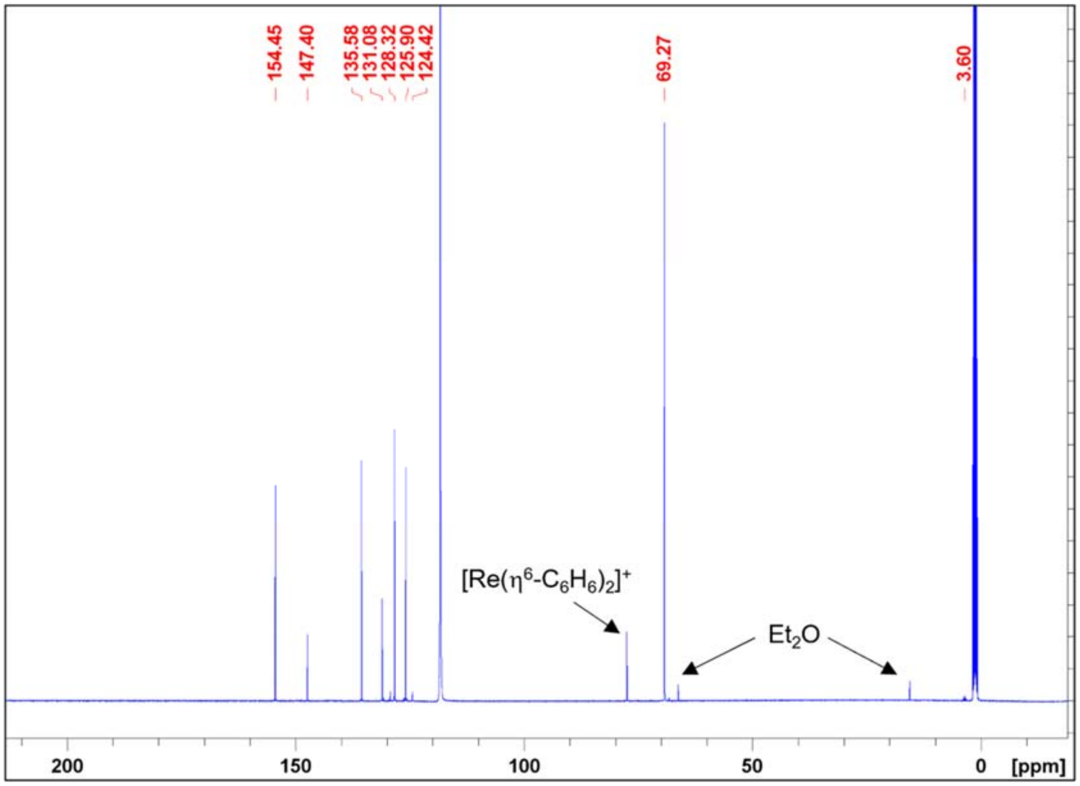

Figure S20: ^{13}C NMR spectrum of [Re(η^{6}-C_6H_6)(phen)($NCCH_3$)](BF_4) ([**8**](BF_4)) in CD_3CN.

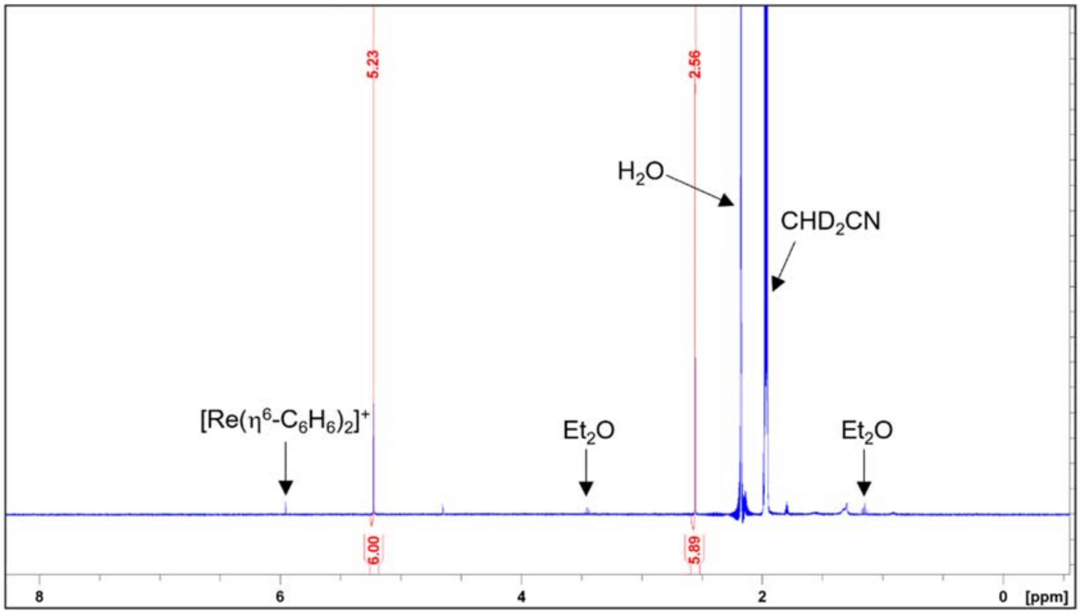

Figure S21: ^{1}H NMR spectrum of $[Re(\eta^6\text{-}C_6H_6)(CO)(NCCH_3)_2](BF_4)$ ([**9**](BF_4)) in CD_3CN.

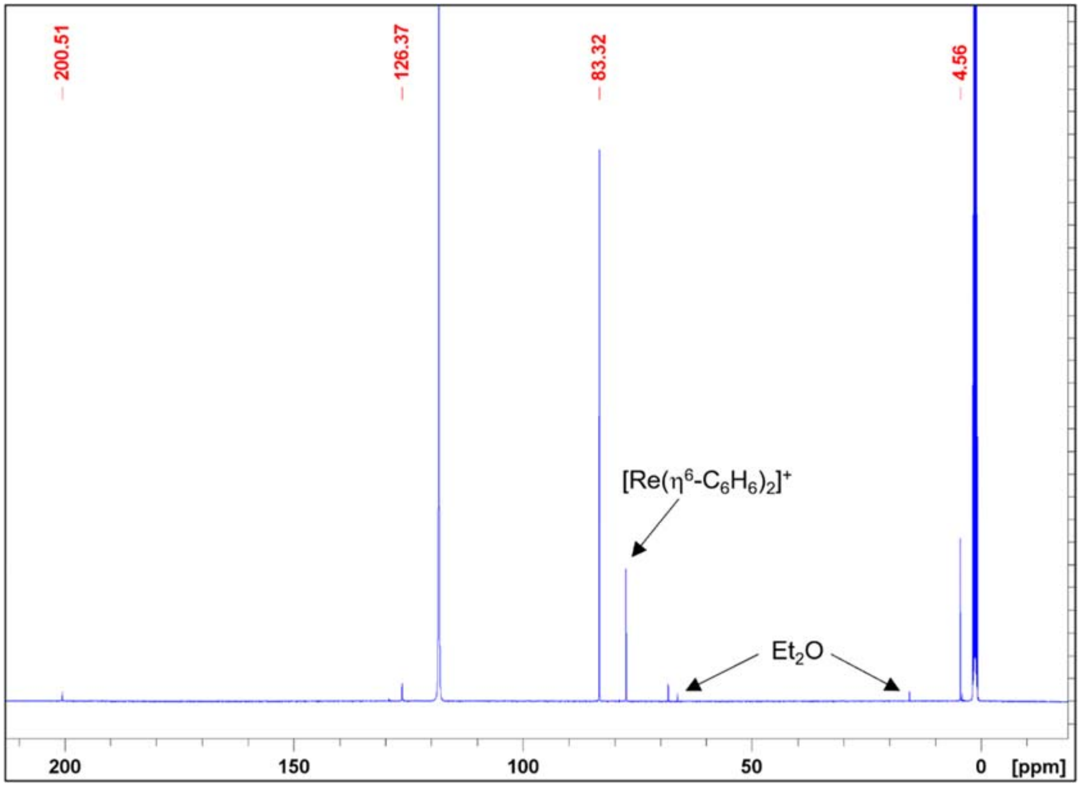

Figure S22: ^{13}C NMR spectrum of $[Re(\eta^6\text{-}C_6H_6)(CO)(NCCH_3)_2](BF_4)$ ([**9**](BF_4)) in CD_3CN.

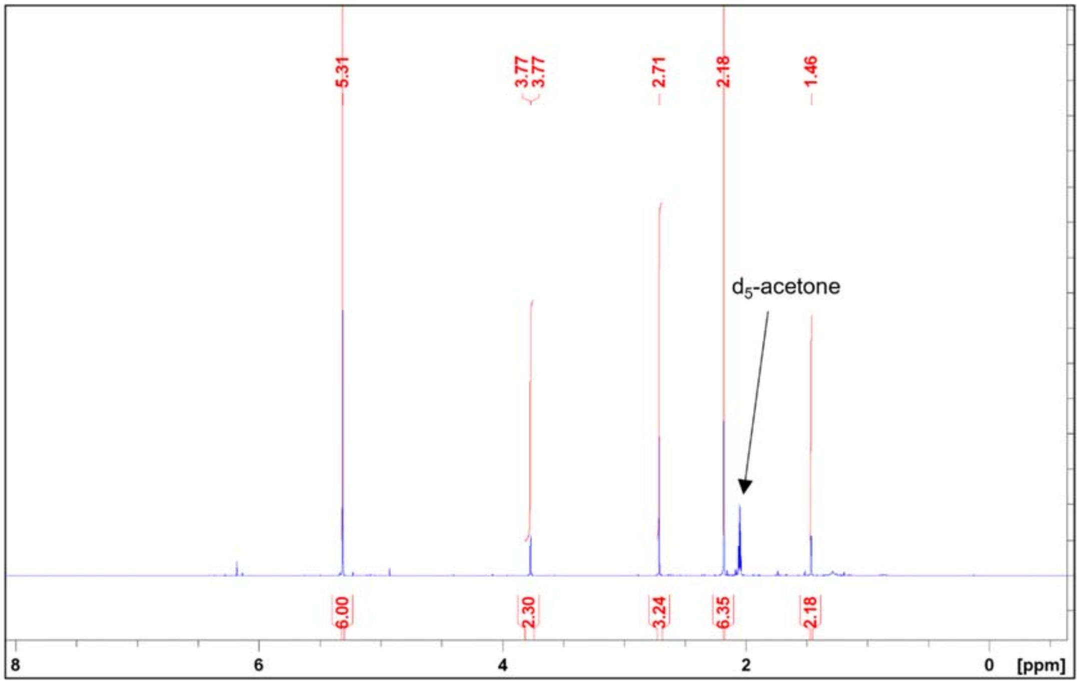

Figure S23: ^{1}H NMR spectrum of [Re(η^6-C_6H_6)(η^4-DMBD)($NCCH_3$)](PF_6) ([**10**](PF_6)) in d_6-acetone.

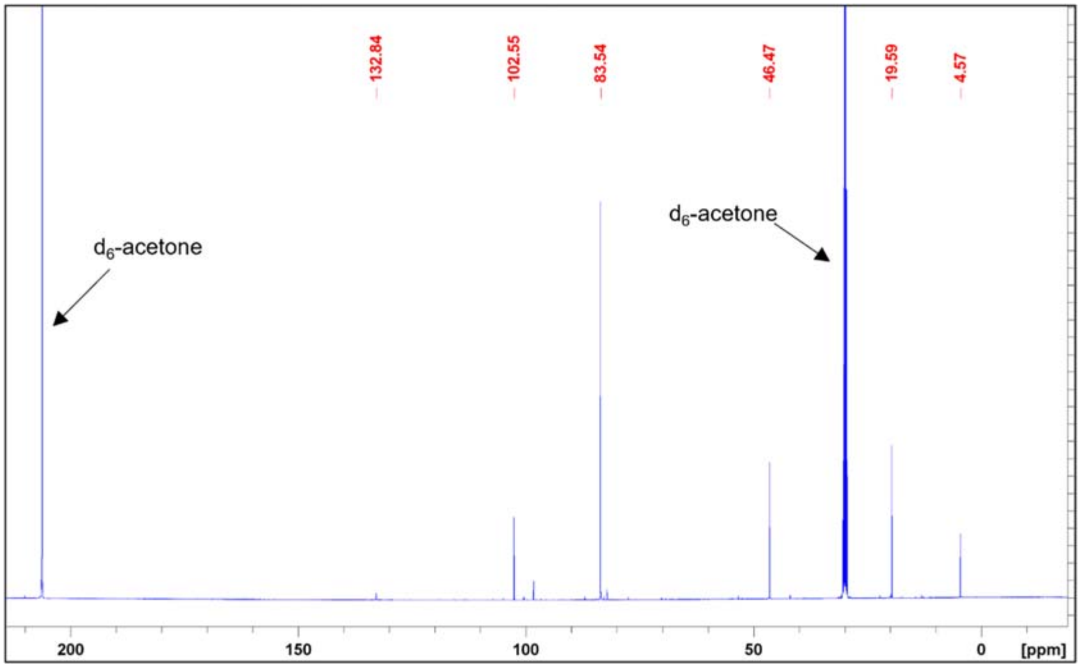

Figure S24: ^{13}C NMR spectrum of [Re(η^6-C_6H_6)(η^4-DMBD)($NCCH_3$)](BF_4) ([**10**](PF_6)) in d_6-acetone. Note: DEPT-135 experiments suggest that the signal at δ = 98.2 ppm originates from a methine or methyl C-atom.

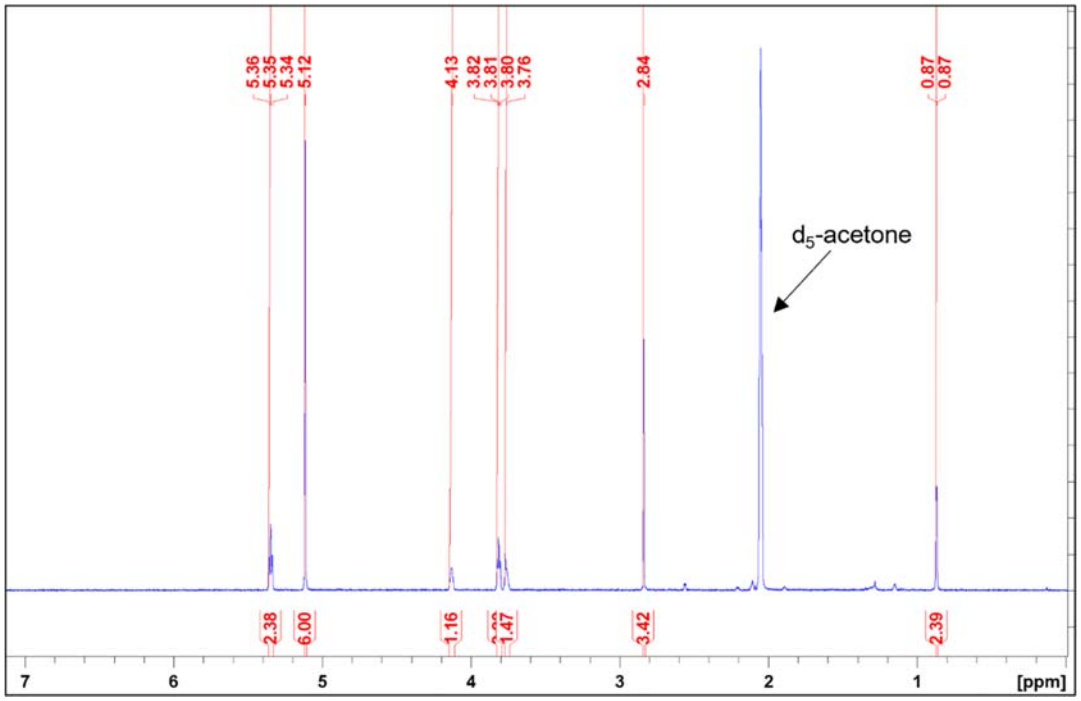

Figure S25: ^{1}H NMR spectrum of $[Re(\eta^6\text{-}C_6H_6)(\eta^4\text{-NBD})(NCCH_3)](PF_6)$ ([**11**](PF_6)) in d_6-acetone.

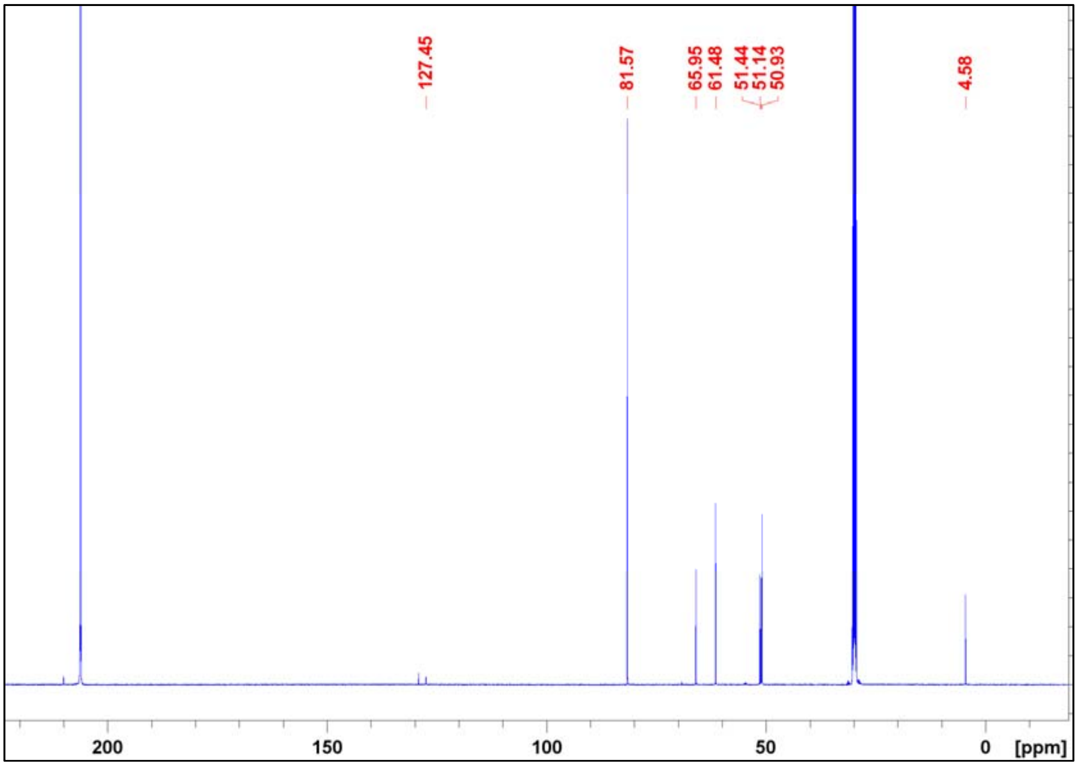

Figure S26: ^{13}C NMR spectrum of $[Re(\eta^6\text{-}C_6H_6)(\eta^4\text{-NBD})(NCCH_3)](PF_6)$ ([**11**](PF_6)) in d_6-acetone. Assignment of the ^{13}C NMR signal of the quaternary C-atom of the coordinated CH_3CN in $[\mathbf{11}]^+$ (δ = 127.5 ppm) performed by HMBC experiments.

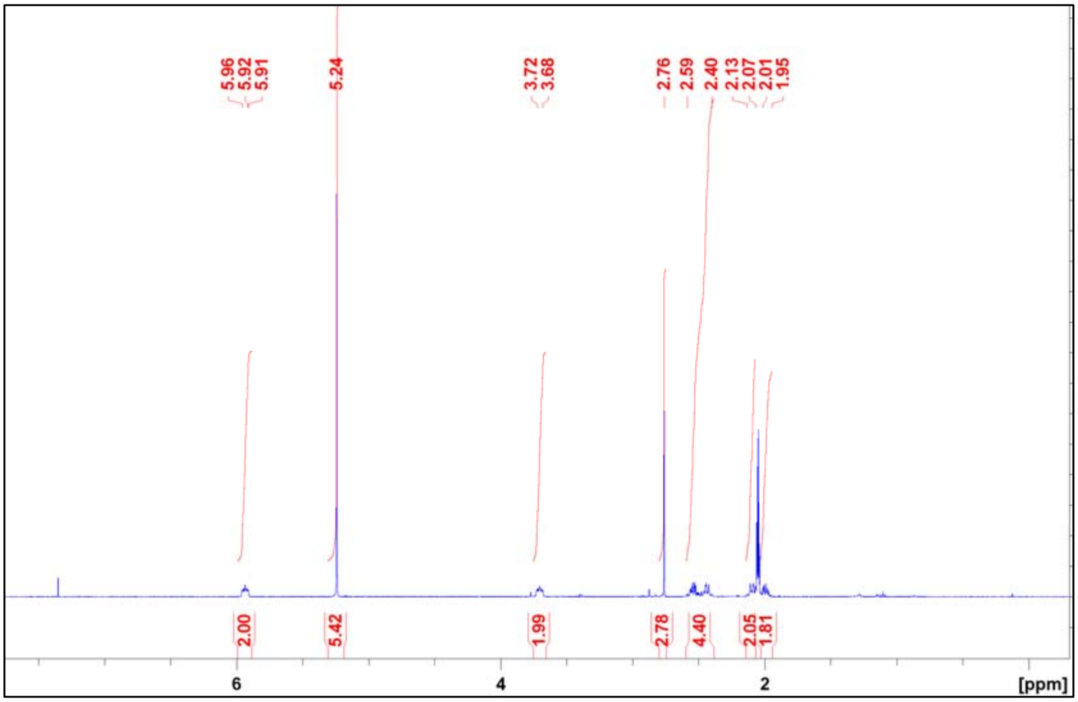

Figure S27: ^{1}H NMR spectrum of $[Re(\eta^6\text{-}C_6H_6)(\eta^4\text{-COD})(NCCH_3)](PF_6)$ ([**12**](PF_6)) in d_6-acetone.

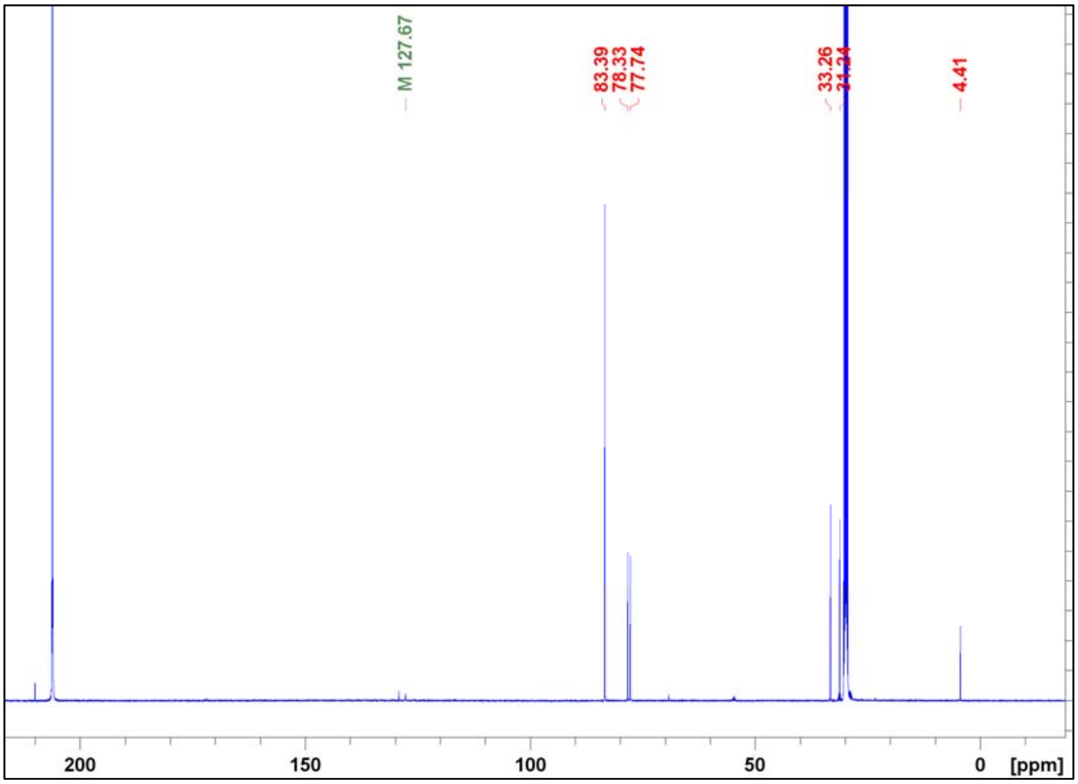

Figure S28: ^{13}C NMR spectrum of $[Re(\eta^6\text{-}C_6H_6)(\eta^4\text{-COD})(NCCH_3)](BF_4)$ ([**12**](PF_6)) in d_6-acetone. Assignment of the ^{13}C NMR signal of the quaternary C-atom of the coordinated CH_3CN in [**12**]$^+$ (δ = 127.7 ppm) performed by HMBC experiments.

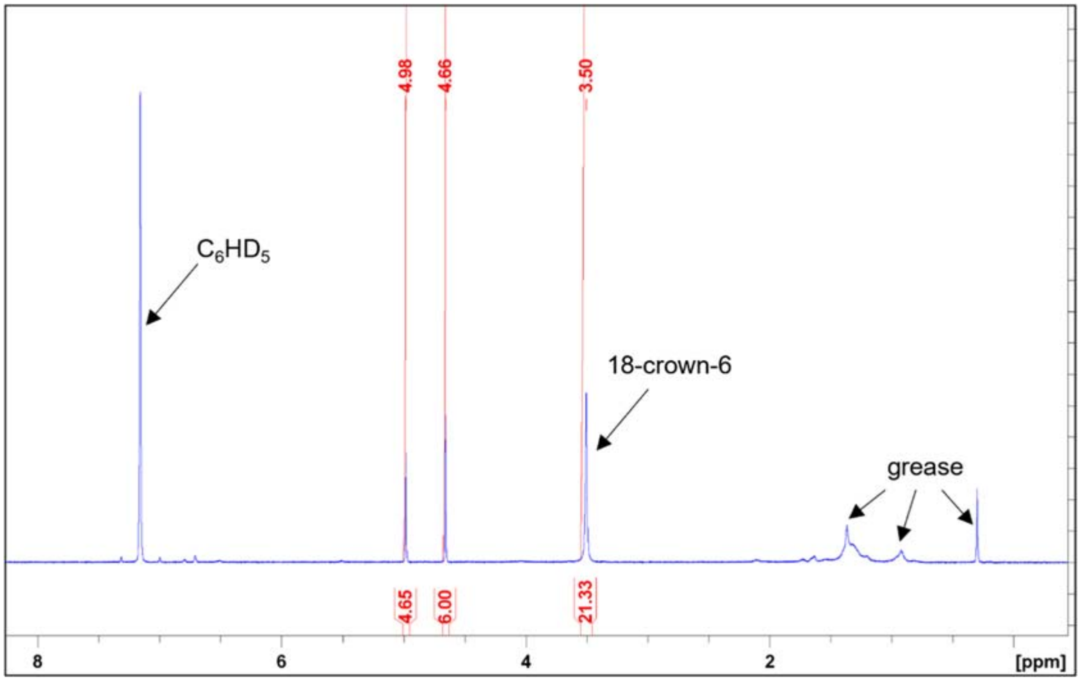

Figure S29: ^{1}H NMR spectrum of $[Re(\eta^5-C_5H_5)(\eta^6-C_6H_6)]$ ([**13**]) in C_6D_6.

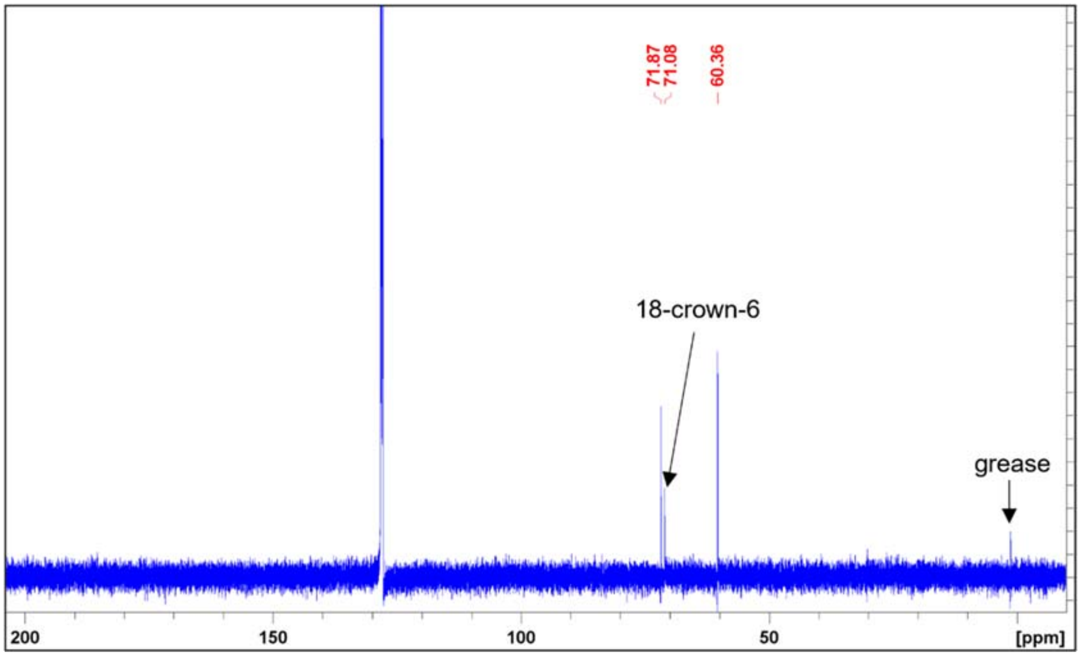

Figure S30: ^{13}C NMR spectrum of $[Re(\eta^5-C_5H_5)(\eta^6-C_6H_6)]$ ([**13**]) in C_6D_6.

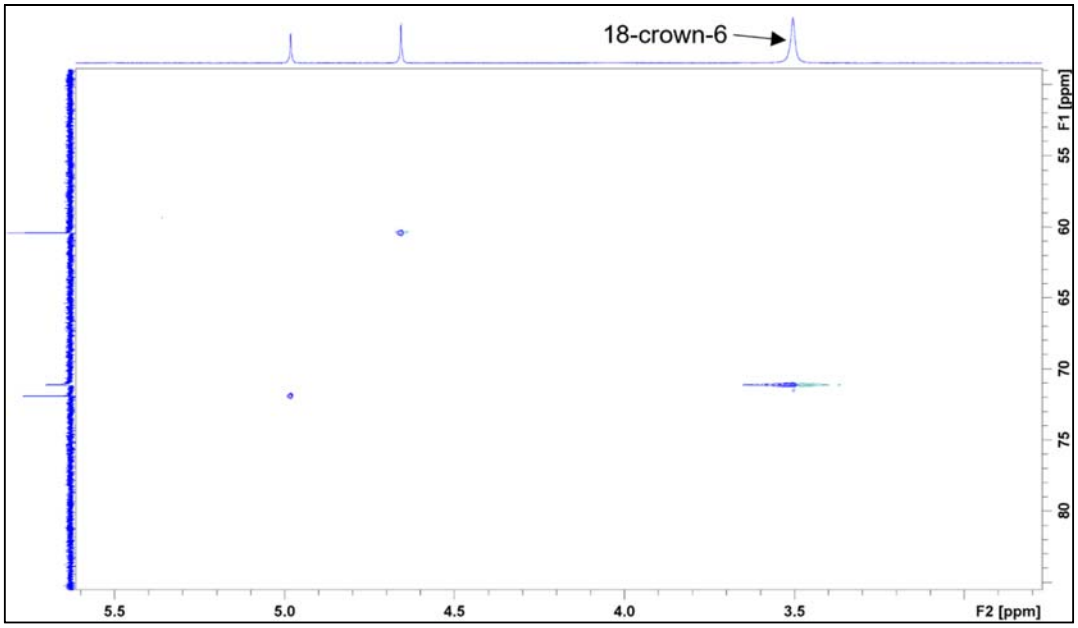

Figure S31: HSQC NMR spectrum of $[Re(\eta^5\text{-}C_5H_5)(\eta^6\text{-}C_6H_6)]$ ([**13**]) in C_6D_6.

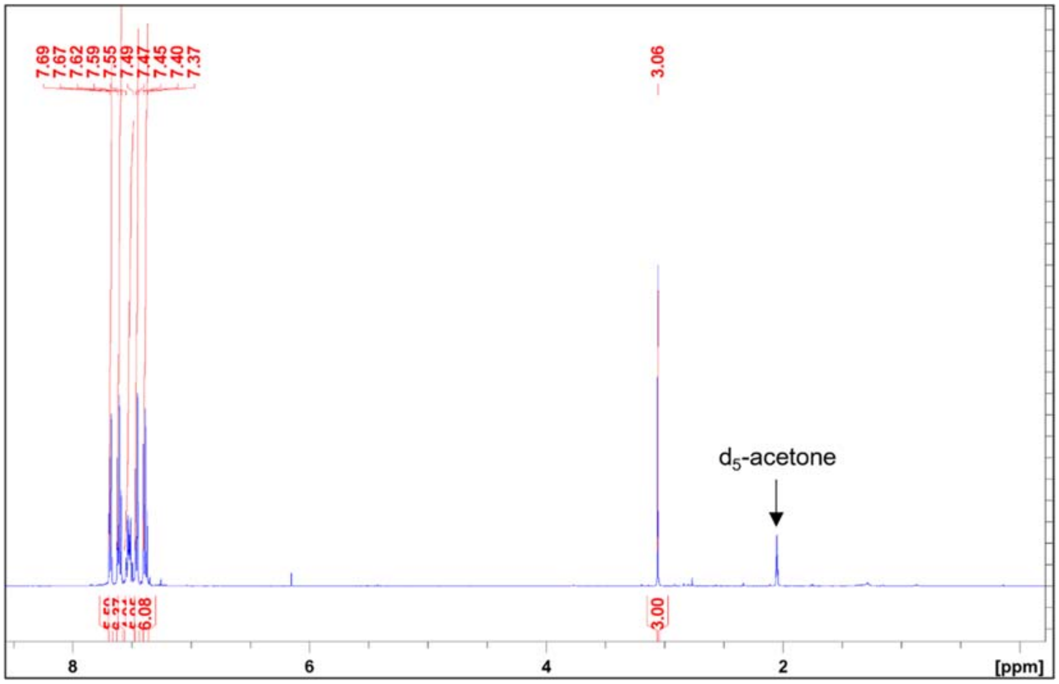

Figure S32: ^{1}H NMR spectrum of $[Re(NCCH_3)(\eta^2\text{-}PhCCPh)_3](OTf)$ ([**14**](OTf)) in d_6-acetone.

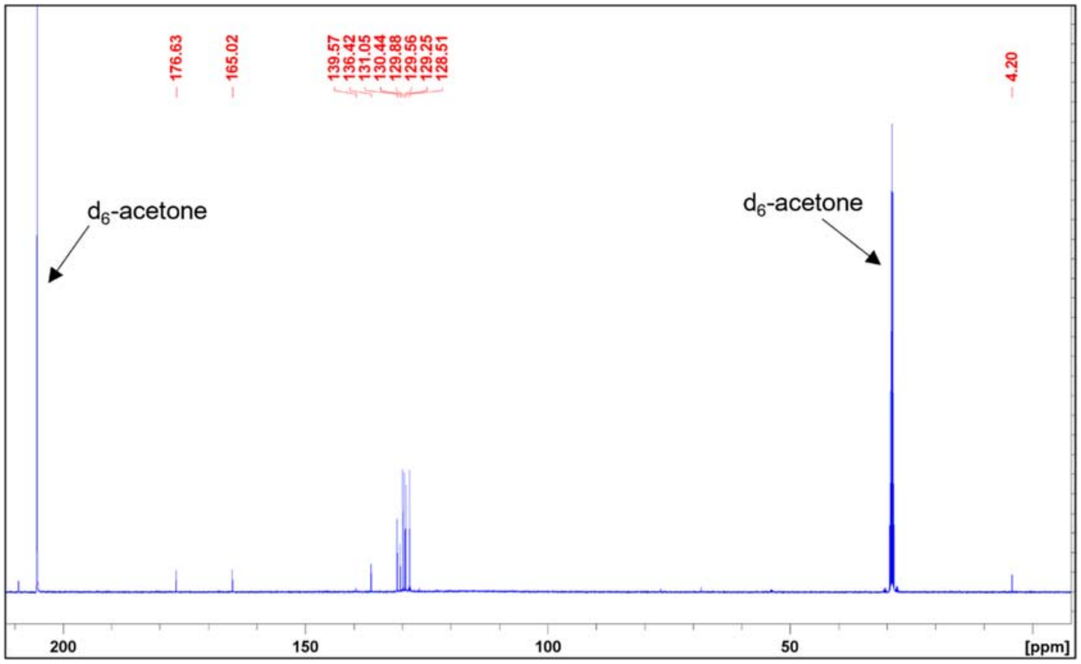

Figure S33: ^{13}C NMR spectrum of [Re(NCCH$_3$)(η^2-PhCCPh)$_3$](OTf) ([**14**](OTf)) in d$_6$-acetone.

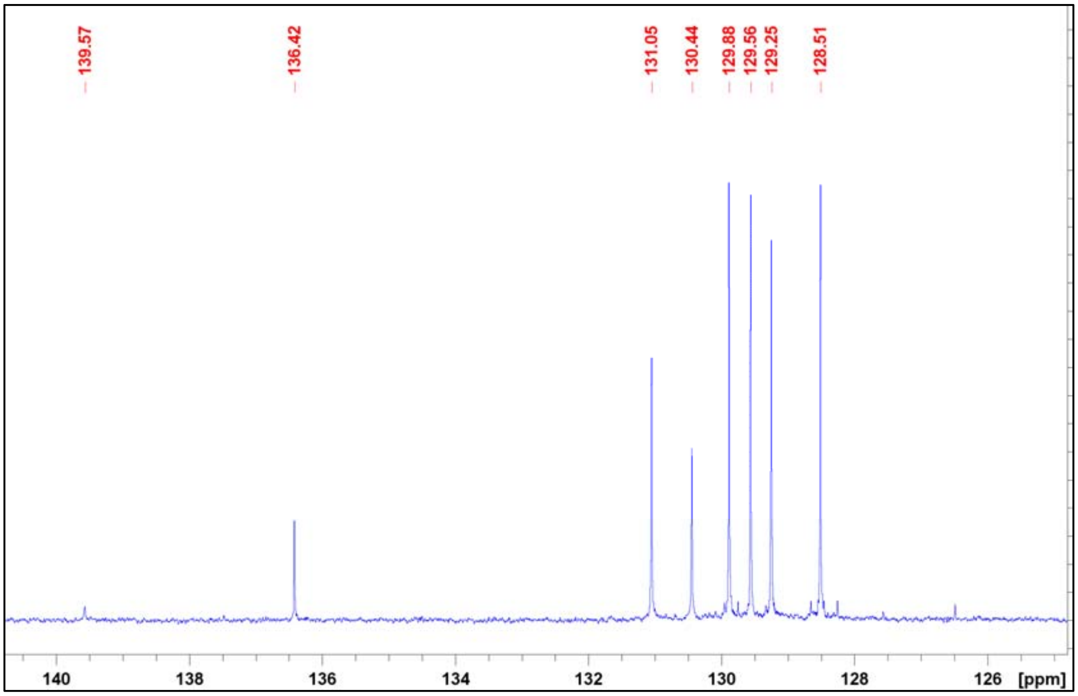

Figure S34: Detailed view on the ^{13}C NMR spectrum (δ = 140 – 126 ppm) of [Re(NCCH$_3$)(η^2-PhCCPh)$_3$](OTf) ([**14**](OTf)) in d$_6$-acetone. Note: Only one signal for the *ipso*-C atoms was found (δ = 136.4 ppm). The signal at δ = 139.6 ppm belongs to the quaternary C-atom of the coordinated CH_3CN ligand. Assignment of the ^{13}C NMR signals performed by HMBC experiments.

2 NMR Data of Ligands

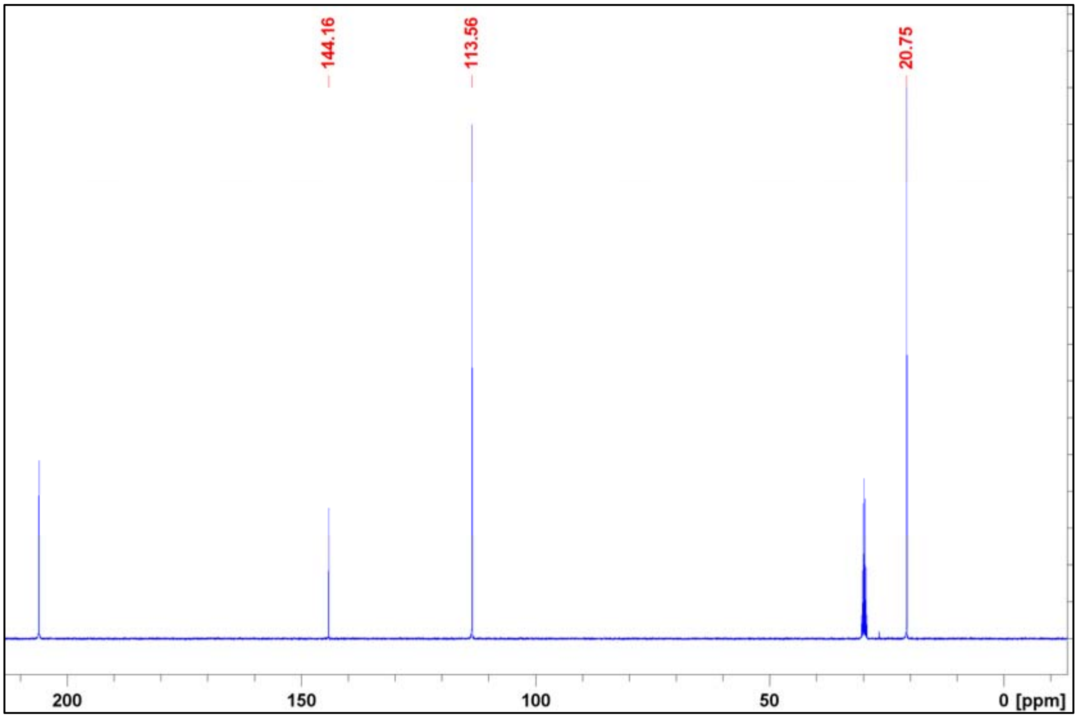

Figure S35: ^{13}C NMR spectrum of 2,3-dimethyl-1,3-butadiene (DMBD) in d_6-acetone.

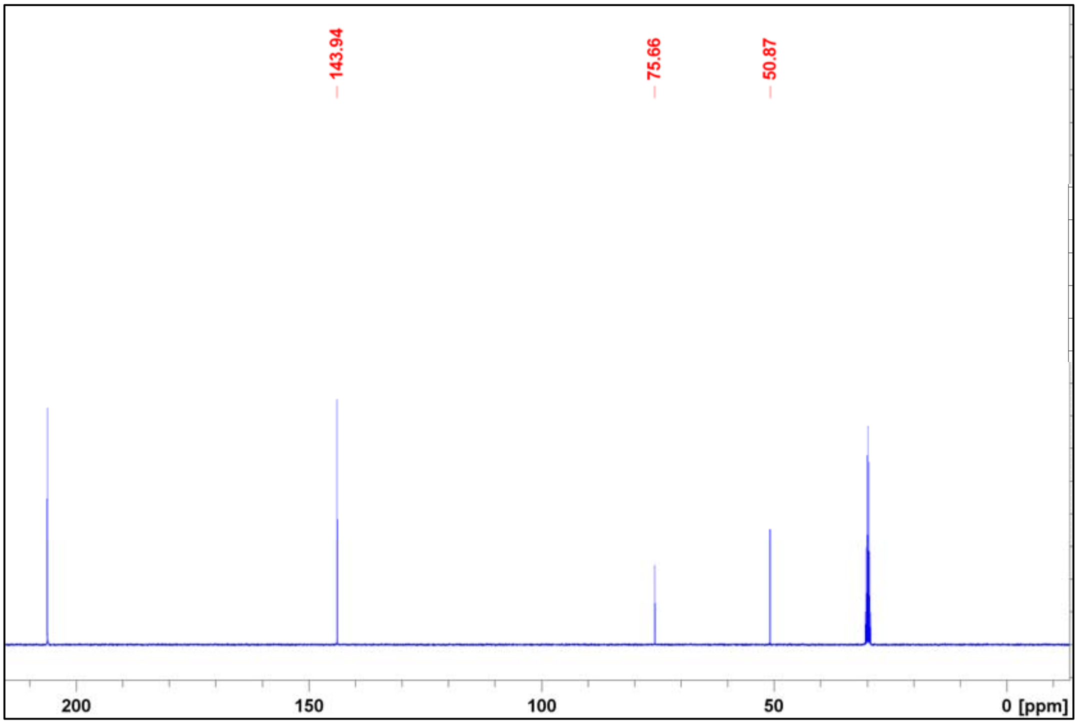

Figure S36: ^{13}C NMR spectrum of norbornadiene (NBD) in d_6-acetone.

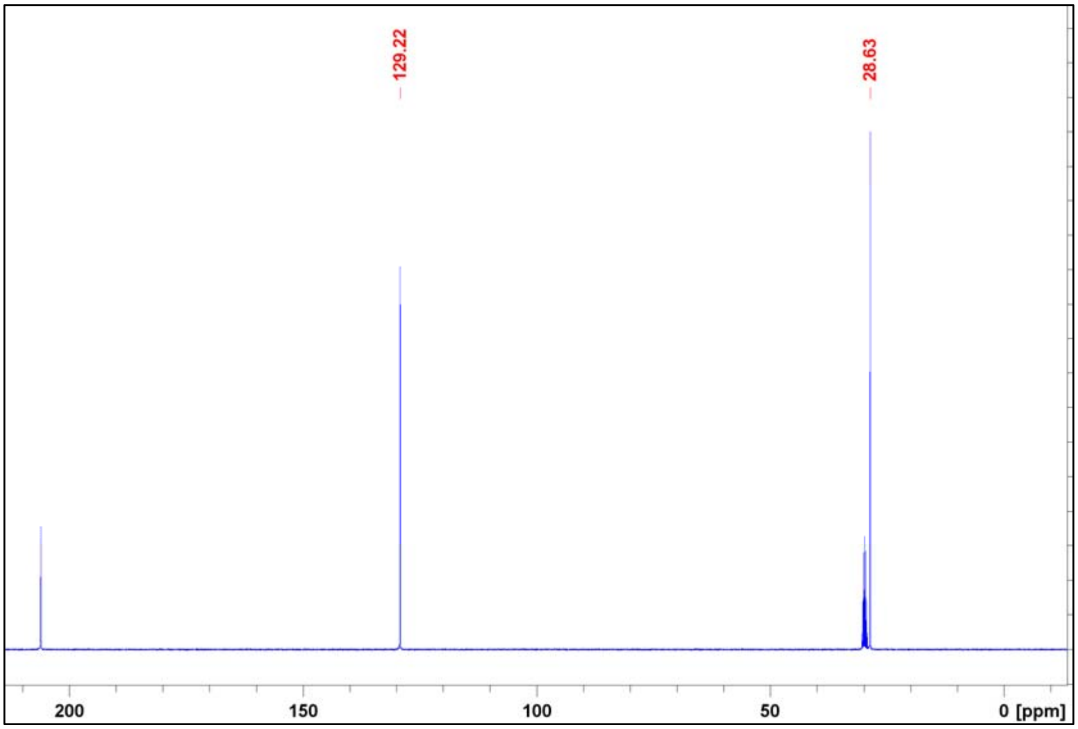

Figure S37: ^{13}C NMR spectrum of 1,5-cyclooctadiene (COD) in d_6-acetone.

3 Electrochemistry

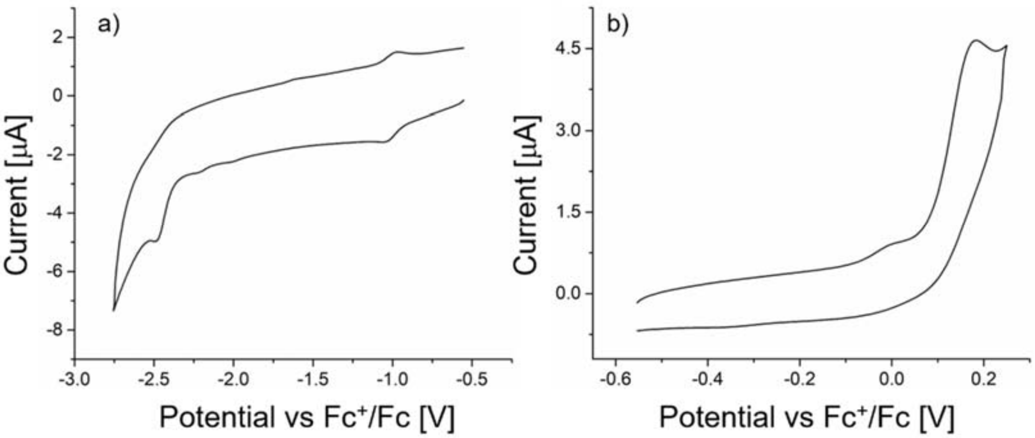

Figure S38: Cyclic voltammogram of [Re(η^6-C_6H_6)($NCCH_3$)$_3$](BF_4) ([**2**](BF_4)) in CH_3CN with (NBu_4)(PF_6) (0.1 M) as electrolyte, depicting its a) reduction and b) oxidation, respectively. Note: The reversible reduction in a) at around – 1.00 V originates from the reduction of $[Re(NCCH_3)_6]^{2+}$, present as small contaminant in [**2**](BF_4).

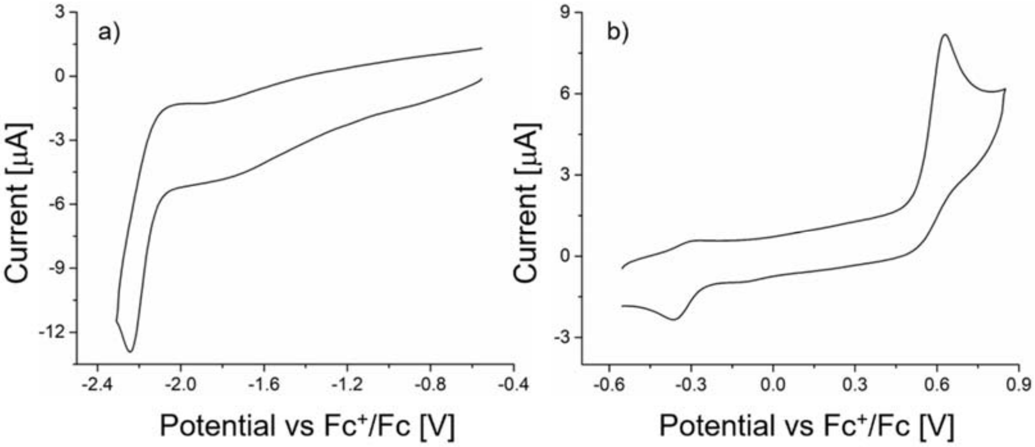

Figure S39: Cyclic voltammogram of [Re(η^6-C_6H_6)(CO)($NCCH_3$)$_2$](BF_4) ([**9**](BF_4)) in CH_3CN with (NBu_4)(PF_6) (0.1 M) as electrolyte, depicting its a) reduction and b) oxidation, respectively.

4 UV-Vis Spectroscopy

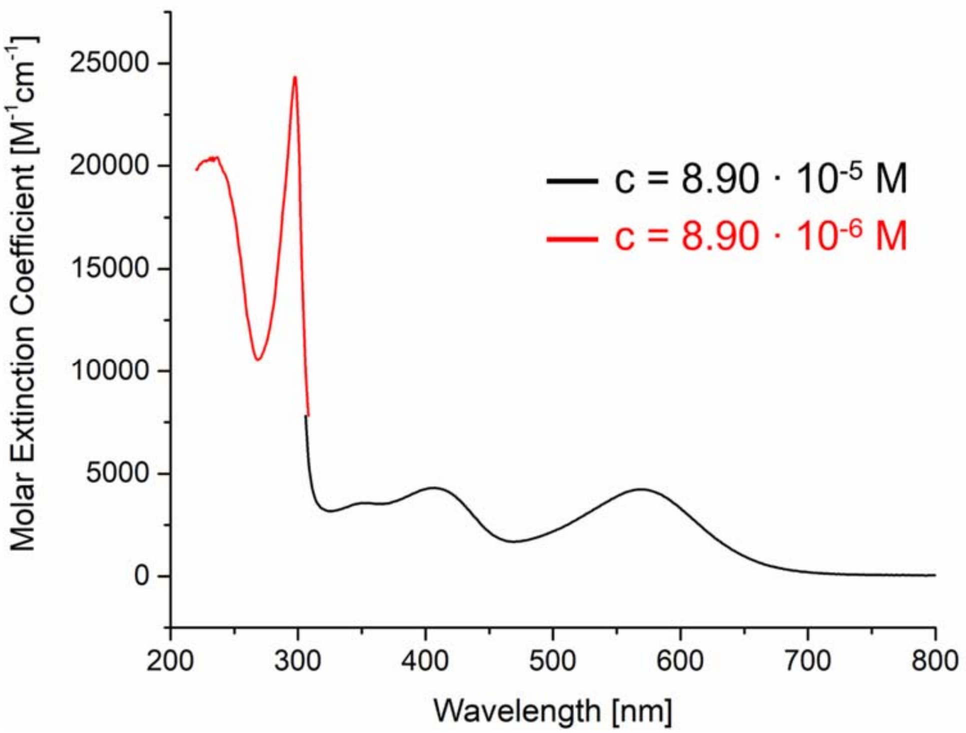

Figure S40: UV/vis spectrum of [Re(η^6-C_6H_6)(bipy)($NCCH_3$)](BF_4) ([**7**](BF_4)) in CH_3CN. Black trace: concentration (c) = $8.90 \cdot 10^{-5}$ M; red trace: c = $8.90 \cdot 10^{-6}$ M.

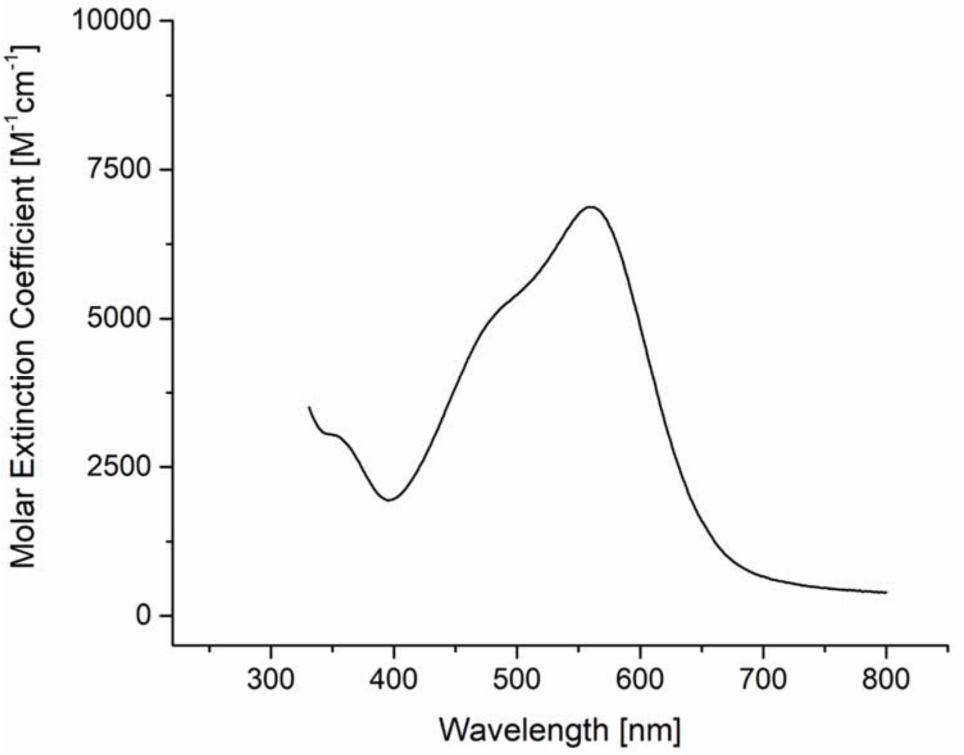

Figure S41: UV/vis spectrum of [Re(η^6-C_6H_6)(phen)($NCCH_3$)](BF_4) ([**8**](BF_4)) in CH_3CN.

5 Crystallographic Details

Table S1: Crystallographic details for [**2**](BF_4) · 0.5 CH_3CN and [**3**](BF_4).

Crystal Structure	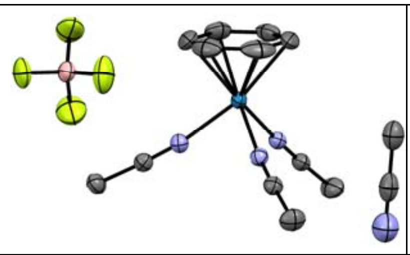	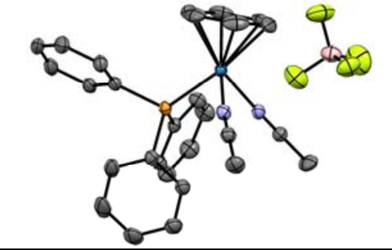
Compound	**[2](BF_4) · 0.5 CH_3CN**	**[3](BF_4)**
Empirical formula	$C_{26}H_{33}B_2F_8N_7Re_2$	$C_{28}H_{27}BF_4N_2PRe$
Formula weight	989.61	695.49
Temperature/K	160(1)	160(1)
Crystal system	monoclinic	triclinic
Space group	I2/a	P-1
a/Å	21.4875(4)	10.20720(10)
b/Å	6.88260(10)	15.4885(2)
c/Å	23.5536(5)	17.5951(2)
α/°	90	75.5340(10)
β/°	110.868(2)	81.3400(10)
γ/°	90	89.1170(10)
Volume/$Å^3$	3254.84(11)	2662.07(5)
Z	4	4
$\rho_{calc}g/cm^3$	2.020	1.735
μ/mm^{-1}	7.507	9.916
F(000)	1880.0	1360.0
Crystal size/mm^3	0.26 × 0.14 × 0.04	0.25 × 0.19 × 0.07
Radiation	Mo Kα (λ = 0.71073)	Cu Kα (λ = 1.54184)
2Θ range for data collection/°	3.702 to 61.014	5.248 to 148.972
Index ranges	-30 ≤ h ≤ 30, -9 ≤ k ≤ 9, -32 ≤ l ≤ 33	-12 ≤ h ≤ 12, -19 ≤ k ≤ 19, -21 ≤ l ≤ 21
Reflections collected	31225	108181
Independent reflections	4971 [R_{int} = 0.0318, R_{sigma} = 0.0205]	10748 [R_{int} = 0.0309, R_{sigma} = 0.0117]
Data/restraints/parameters	4971/244/246	10748/0/672
Goodness-of-fit on F^2	1.041	1.158
Final R indexes [I>=2σ (I)]	R_1 = 0.0194, wR_2 = 0.0432	R_1 = 0.0205, wR_2 = 0.0503
Final R indexes [all data]	R_1 = 0.0240, wR_2 = 0.0454	R_1 = 0.0209, wR_2 = 0.0507
Largest diff. peak/hole / e $Å^{-3}$	0.81/-0.74	0.65/-0.59

Table S2: Crystallographic details for [**4**](BF_4) and [**5**](BF_4).

Crystal Structure	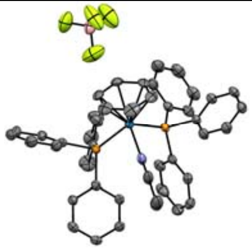	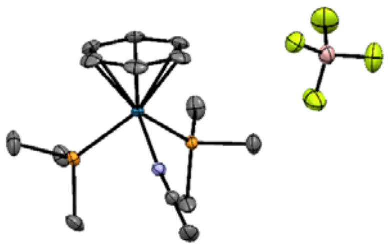
Compound	**[4](BF_4)**	**[5](BF_4)**
Empirical formula	$C_{44}H_{39}BF_4NP_2Re$	$C_{14}H_{27}BF_4NP_2Re$
Formula weight	916.71	544.31
Temperature/K	160(1)	160.0(1)
Crystal system	orthorhombic	orthorhombic
Space group	$P2_12_12$	Fdd2
a/Å	20.15350(10)	32.0682(4)
b/Å	18.54730(10)	29.1987(4)
c/Å	10.10790(10)	16.6667(2)
α/°	90	90
β/°	90	90
γ/°	90	90
Volume/Å^3	3778.26(5)	15605.9(3)
Z	4	32
ρ_{calc}g/cm^3	1.612	1.853
μ/mm^{-1}	7.532	6.424
F(000)	1824.0	8448.0
Crystal size/mm^3	0.11 × 0.06 × 0.04	0.1 × 0.1 × 0.05
Radiation	Cu Kα (λ = 1.54184)	Mo Kα (λ = 0.71073)
2Θ range for data collection/°	6.476 to 148.94	4.738 to 61.014
Index ranges	$-25 \le h \le 25, -23 \le k \le 22, -12 \le l \le 10$	$-45 \le h \le 45, -41 \le k \le 41, -23 \le l \le 23$
Reflections collected	38886	147507
Independent reflections	7728 [R_{int} = 0.0271, R_{sigma} = 0.0179]	11887 [R_{int} = 0.0371, R_{sigma} = 0.0153]
Data/restraints/parameters	7728/18/479	11887/135/470
Goodness-of-fit on F^2	1.052	1.041
Final R indexes [I>=2σ (I)]	R_1 = 0.0184, wR_2 = 0.0466	R_1 = 0.0150, wR_2 = 0.0302
Final R indexes [all data]	R_1 = 0.0192, wR_2 = 0.0470	R_1 = 0.0176, wR_2 = 0.0307
Largest diff. peak/hole / e Å^{-3}	0.69/-0.52	0.48/-0.42
Flack parameters	-0.034(3)	-0.008(2)

Table S3: Crystallographic details for [**6**](BF_4) and [**7**](BF_4) · 0.5 CH_3CN.

Crystal Structure	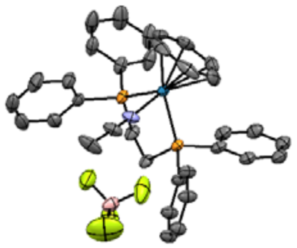	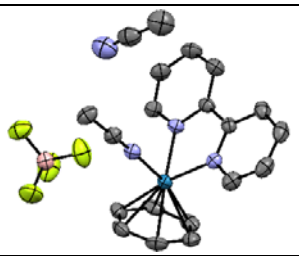
Compound	**[6](BF$_4$)**	**[7](BF$_4$) · 0.5 CH$_3$CN**
Empirical formula	$C_{34}H_{33}BF_4NP_2Re$	$C_{38}H_{37}B_2F_8N_7Re_2$
Formula weight	790.56	1137.76
Temperature/K	160(1)	160.0(1)
Crystal system	monoclinic	monoclinic
Space group	$P2_1/n$	$P2_1/c$
a/Å	8.4500(2)	10.92960(10)
b/Å	19.7085(4)	15.40740(10)
c/Å	18.9425(4)	23.12910(10)
α/°	90	90
β/°	92.769(2)	96.7720(10)
γ/°	90	90
Volume/Å^3	3150.94(12)	3867.69(5)
Z	4	4
ρ_{calc}g/cm^3	1.666	1.954
μ/mm^{-1}	8.917	12.733
F(000)	1560.0	2184.0
Crystal size/mm^3	0.17 × 0.05 × 0.02	0.37 × 0.23 × 0.07
Radiation	Cu Kα (λ = 1.54184)	Cu Kα (λ = 1.54184)
2Θ range for data collection/°	6.476 to 140.142	6.908 to 152.932
Index ranges	-10 ≤ h ≤ 10, -24 ≤ k ≤ 23, -23 ≤ l ≤ 16	-10 ≤ h ≤ 13, -19 ≤ k ≤ 19, -28 ≤ l ≤ 28
Reflections collected	35071	45418
Independent reflections	5988 [R_{int} = 0.0300, R_{sigma} = 0.0199]	8079 [R_{int} = 0.0326, R_{sigma} = 0.0182]
Data/restraints/parameters	5988/988/525	8079/0/518
Goodness-of-fit on F^2	1.034	1.136
Final R indexes [I>=2σ (I)]	R_1 = 0.0213, wR_2 = 0.0538	R_1 = 0.0256, wR_2 = 0.0670
Final R indexes [all data]	R_1 = 0.0232, wR_2 = 0.0553	R_1 = 0.0261, wR_2 = 0.0674
Largest diff. peak/hole / e Å^{-3}	0.83/-0.72	1.36/-1.11

Table S4: Crystallographic details for [**8**](BF_4) · CH_2Cl_2 and [**9**](BF_4).

Crystal Structure	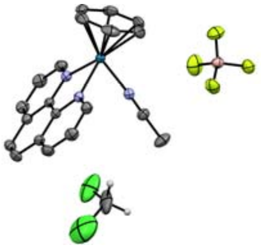	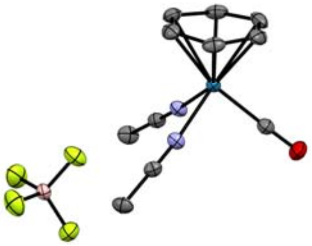
Compound	**[8](BF_4) · CH_2Cl_2**	**[9](BF_4)**
Empirical formula	$C_{41}H_{36}B_2Cl_2F_8N_6Re_2$	$C_{11}H_{12}BF_4N_2ORe$
Formula weight	1229.68	461.24
Temperature/K	160(1)	160(1)
Crystal system	monoclinic	monoclinic
Space group	$P2_1/n$	$P2_1/m$
a/Å	12.7607(1)	6.74570(10)
b/Å	12.5693(1)	10.1488(2)
c/Å	13.1865(1)	10.5783(2)
α/°	90	90
β/°	103.845(1)	108.541(2)
γ/°	90	90
Volume/Å^3	2053.57(3)	686.61(2)
Z	2	2
ρ_{calc}g/cm^3	1.989	2.231
μ/mm^{-1}	6.096	8.890
F(000)	1180.0	432.0
Crystal size/mm^3	0.16 × 0.09 × 0.02	0.33 × 0.19 × 0.11
Radiation	Mo Kα (λ = 0.71073)	Mo Kα (λ = 0.71073)
2Θ range for data collection/°	3.99 to 61.014	5.71 to 61.01
Index ranges	$-18 \leq h \leq 18$, $-17 \leq k \leq 17$, $-18 \leq l \leq 18$	$-9 \leq h \leq 9$, $-14 \leq k \leq 14$, $-15 \leq l \leq 15$
Reflections collected	44712	11396
Independent reflections	6266 [R_{int} = 0.0308, R_{sigma} = 0.0192]	2205 [R_{int} = 0.0467, R_{sigma} = 0.0322]
Data/restraints/parameters	6266/19/290	2205/0/102
Goodness-of-fit on F^2	1.040	1.037
Final R indexes [I>=2σ (I)]	R_1 = 0.0178, wR_2 = 0.0363	R_1 = 0.0191, wR_2 = 0.0416
Final R indexes [all data]	R_1 = 0.0228, wR_2 = 0.0382	R_1 = 0.0215, wR_2 = 0.0424
Largest diff. peak/hole / e Å^{-3}	0.67/-0.66	0.78/-0.66

Table S5: Crystallographic details for [**10**](PF_6) · CH_3CN and [**12**](PF_6).

Crystal Structure	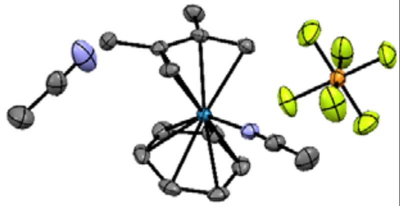	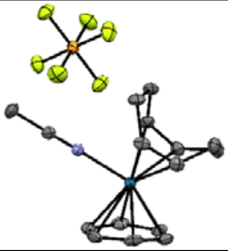
Compound	**[10](PF_6) · CH_3CN**	**[12](PF_6)**
Empirical formula	$C_{16}H_{22}F_6N_2PRe$	$C_{16}H_{21}F_6NPRe$
Formula weight	573.52	558.51
Temperature/K	160(1)	160(1)
Crystal system	monoclinic	orthorhombic
Space group	P21/n	Pna21
a/Å	7.98440(10)	15.53150(10)
b/Å	20.1302(2)	14.92870(10)
c/Å	11.85190(10)	7.44500(10)
α/°	90	90
β/°	92.7030(10)	90
γ/°	90	90
Volume/Å^3	1902.81(3)	1726.24(3)
Z	4	4
ρ_{calc}g/cm^3	2.002	2.149
μ/mm^{-1}	6.528	15.222
F(000)	1104.0	1072.0
Crystal size/mm^3	0.17 × 0.1 × 0.09	0.27 × 0.13 × 0.02
Radiation	Mo Kα (λ = 0.71073)	Cu Kα (λ = 1.54184)
2Θ range for data collection/°	3.992 to 61.012	8.214 to 136.406
Index ranges	-11 ≤ h ≤ 11, -28 ≤ k ≤ 28, -16 ≤ l ≤ 16	-18 ≤ h ≤ 17, -17 ≤ k ≤ 17, -7 ≤ l ≤ 8
Reflections collected	47185	34619
Independent reflections	5798 [R_{int} = 0.0304, R_{sigma} = 0.0168]	3075 [R_{int} = 0.0271, R_{sigma} = 0.0100]
Data/restraints/parameters	5798/18/252	3075/1/227
Goodness-of-fit on F^2	1.048	1.062
Final R indexes [I>=2σ (I)]	R_1 = 0.0153, wR_2 = 0.0318	R_1 = 0.0175, wR_2 = 0.0504
Final R indexes [all data]	R_1 = 0.0194, wR_2 = 0.0333	R_1 = 0.0177, wR_2 = 0.0506
Largest diff. peak/hole / e Å^{-3}	0.54/-0.53	0.29/-0.88
Flack parameters		0.033(13)

Table S6: Crystallographic details for [**13**] and [**14**](OTf).

Crystal Structure	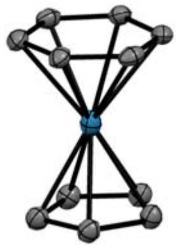	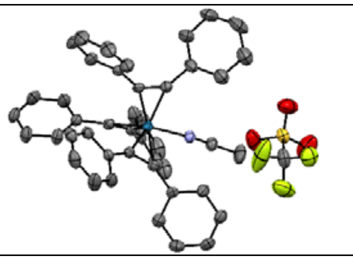
Compound	**[13]**	**[14](OTf)**
Empirical formula	$C_{11}H_{11}Re$	$C_{45}H_{33}F_3NO_3ReS$
Formula weight	329.40	910.98
Temperature/K	160(1)	160(1)
Crystal system	orthorhombic	monoclinic
Space group	Cmce	P21/c
a/Å	9.2959(2)	22.6667(1)
b/Å	7.3537(2)	17.8910(1)
c/Å	12.7093(4)	19.5686(1)
α/°	90	90
β/°	90	96.891(1)
γ/°	90	90
Volume/Å^3	868.80(4)	7878.33(7)
Z	4	8
ρ_{calc}g/cm^3	2.518	1.536
μ/mm^{-1}	13.920	6.988
F(000)	608.0	3616.0
Crystal size/mm^3	0.1 × 0.07 × 0.02	0.21 × 0.16 × 0.05
Radiation	Mo Kα (λ = 0.71073)	Cu Kα (λ = 1.54184)
2Θ range for data collection/°	6.412 to 55.754	3.926 to 148.986
Index ranges	-12 ≤ h ≤ 12, -9 ≤ k ≤ 9, -16 ≤ l ≤ 16	-28 ≤ h ≤ 23, -22 ≤ k ≤ 22, -24 ≤ l ≤ 24
Reflections collected	5958	82233
Independent reflections	552 [R_{int} = 0.0265, R_{sigma} = 0.0124]	16081 [R_{int} = 0.0246, R_{sigma} = 0.0168]
Data/restraints/parameters	552/172/82	16081/0/975
Goodness-of-fit on F^2	1.130	1.064
Final R indexes [I>=2σ (I)]	R_1 = 0.0130, wR_2 = 0.0279	R_1 = 0.0305, wR_2 = 0.0772
Final R indexes [all data]	R_1 = 0.0168, wR_2 = 0.0294	R_1 = 0.0323, wR_2 = 0.0782
Largest diff. peak/hole / e Å^{-3}	0.49/-0.97	1.99/-0.66

Special Crystallographic Features

[**2**](BF_4) · 0.5 CH_3CN: The F atoms of the BF_4^- ion are disordered over two sets of positions with site-occupancy factors of 0.159(3) and 0.841(3). The main ions co-crystallized with solvent molecules of acetonitrile in a ratio 2/2/1, respectively.

[**6**](BF_4): The dppe ligand is partially disordered over two sets of positions with site-occupancy factors of 0.433(4) and 0.567(4).

[**7**](BF_4): Two cations, two anions, and one solvent molecule of acetonitrile in the asymmetric unit.

[**8**](BF_4) · CH_2Cl_2: The solvent molecule of dichloromethane is disordered over two sets of positions around a center of inversion (site occupancy factor of 0.5).

[**9**](BF_4): The ions are located on mirror planes.

[**13**]: The model is highly disordered. The six-membered ring is disordered over two sets of positions (site-occupancy factors 0.5) while the five-membered ring is disordered over four sets of positions (site-occupancy factors 0.208(6) and 0.292(6)). The molecule lies on several symmetry elements: two-fold axis, center of inversion and mirror plane.

[**14**](OTf): The asymmetric unit contains two cationic rhenium complexes and two counterions.
